# Hybridization of Atlantic puffins in the Arctic coincides with 20th-century climate change

**DOI:** 10.1126/sciadv.adh1407

**Published:** 2023-10-06

**Authors:** Oliver Kersten, Bastiaan Star, Anders K. Krabberød, Lane M. Atmore, Ole K. Tørresen, Tycho Anker-Nilssen, Sébastien Descamps, Hallvard Strøm, Ulf S. Johansson, Paul R. Sweet, Kjetill S. Jakobsen, Sanne Boessenkool

**Affiliations:** ^1^ Centre for Ecological and Evolutionary Synthesis (CEES), Department of Biosciences, University of Oslo, Oslo, Norway.; ^2^ Section for Genetics and Evolutionary Biology (Evogene), Department of Biosciences, University of Oslo, Oslo, Norway.; ^3^ Norwegian Institute for Nature Research (NINA), Trondheim, Norway.; ^4^ Norwegian Polar Institute, Fram Centre, Langnes, Tromsø, Norway.; ^5^ Swedish Museum of Natural History, Stockholm, Sweden.; ^6^ American Museum of Natural History, New York, NY, USA.

## Abstract

The Arctic is experiencing the fastest rates of global warming, leading to shifts in the distribution of its biota and increasing the potential for hybridization. However, genomic evidence of recent hybridization events in the Arctic remains unexpectedly rare. Here, we use whole-genome sequencing of contemporary and 122-year-old historical specimens to investigate the origin of an Arctic hybrid population of Atlantic puffins (*Fratercula arctica*) on Bjørnøya, Norway. We show that the hybridization between the High Arctic, large-bodied subspecies *F. a. naumanni* and the temperate, smaller-sized subspecies *F. a. arctica* began as recently as six generations ago due to an unexpected southward range expansion of *F. a. naumanni.* Moreover, we find a significant temporal loss of genetic diversity across Arctic and temperate puffin populations. Our observations provide compelling genomic evidence of the impacts of recent distributional shifts and loss of diversity in Arctic communities during the 20th century.

## Teaser

Temporal genomics reveal that hybridization of Atlantic puffins coincides with rapid climate change of the 20th century.

## INTRODUCTION

Global warming is driving flora and fauna toward the poles in search of more favorable environmental conditions (*1*–*3*). Such poleward shifts in species’ distributions increase the potential for previously geographically isolated taxa or populations to hybridize. The evolutionary consequences of hybridization are complex, for instance, leading to decreased hybrid fitness, the extinction of parental and/or hybrid taxa, or to increased levels of genomic diversity promoting the resilience of a species (*4*–*6*). As the Arctic is experiencing the fastest rates of ecological and environmental change in the world (*1*, *3*, *7*), the potential for inter- and intraspecific hybridization is expected to be particularly high in this region (*8*, *9*). Hybridization can be detected through temporal, whole-genome sequencing of historical specimens (*10*–*13*). Such temporal genomic data are crucial for determining if any observations of hybridization can be directly associated with recent environmental change. Nonetheless, temporal genomic approaches have not yet widely been used in the Arctic region (*5*, *8*, *9*, *12*, *13*) and, despite a few high-profile cases [e.g., polar bear (*14*) and snow hare (*15*)], unambiguous examples of recent hybridization in the Arctic remain unexpectedly rare.

**Fig. 1.**
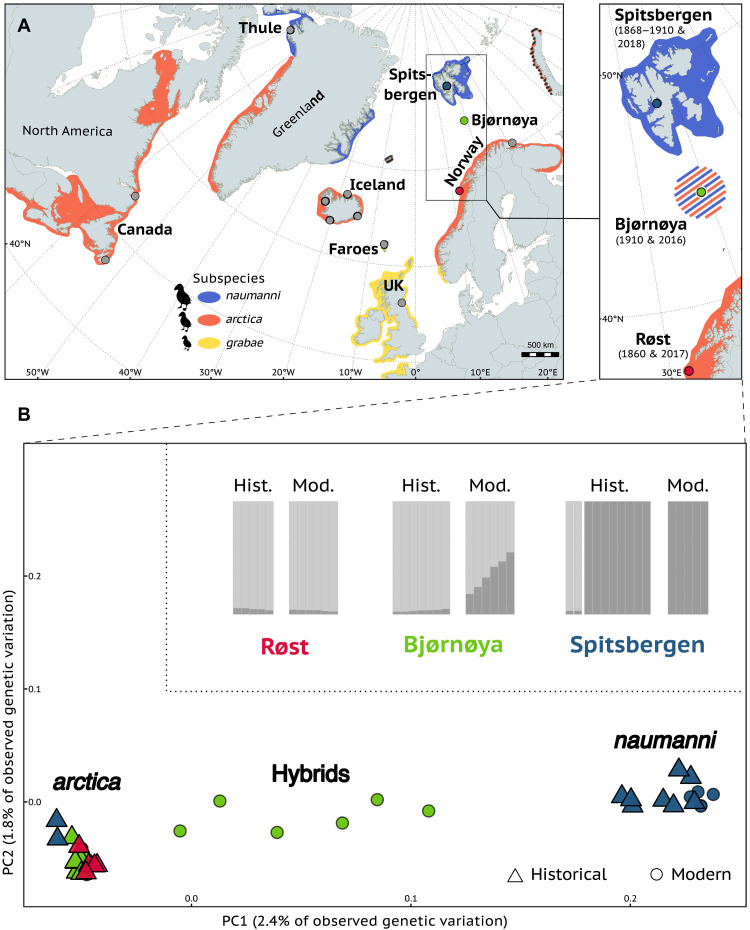
Genomic population structure of modern and historical Atlantic puffins across three breeding colonies. (**A**) Distribution of breeding colonies analyzed in this study projected over the range of the three currently recognized subspecies and Atlantic puffin colonies previously analyzed for population genomics (*19*). The zoomed-in window presents the main study area with the hybrid population on Bjørnøya and the two colonies representing the parental subspecies *F. a. naumanni* and *F. a. arctica* from which historical and modern individuals were analyzed in this study. Sample dates are indicated in parentheses. (**B**) Genomic population structure of modern and historical Atlantic puffins of Bjørnøya, Røst, and Spitsbergen reconstructed by principal components and admixture analyses, performed with *PCAngsd* and *ngsAdmix*, respectively. The other 10 colonies used for the PCA analysis were removed for visualization purposes (the PCA with all colonies is presented in fig. S5). One related individual was removed from the modern dataset (*N* = 17, Spitsbergen = 5, Bjørnøya = 6, Røst = 6) and the historical dataset consisted of 22 individuals (Røst = 5, Bjørnøya = 7, Spitsbergen = 10). The same color coding for colonies and subspecies is used throughout the figure. Hist., historical; Mod., modern.

Seabirds are a conspicuous component of Arctic biodiversity and are heavily affected by climate change leading to broad population declines and northward distributional shifts of temperate species (*16*–*18*). Here, we investigate the origin of a recently recognized Arctic hybrid population of Atlantic puffins [*Fratercula arctica*, Linnaeus, 1789; (*19*)], an iconic seabird that breeds throughout the North Atlantic and the Arctic (Fig. 1A) and that spends the nonbreeding season at sea (*20*, *21*). The Atlantic puffin is currently designated as “vulnerable” to extinction globally and “endangered” in Europe (*22*). Notably, the once world’s largest colony at Røst (Norway) has lost more than 80% of its breeding pairs during the past 40 years (*20*, *23*). Similarly, Icelandic and Faroese puffins have experienced low productivity and negative population growth since 2003 (*24*). These population declines have at least in part been ascribed to changes in prey availability as a result of climate change and overfishing (*20*, *22*, *25*, *26*).

Partially corroborated by whole-genome analyses (*19*), Atlantic puffins are broadly classified into three subspecies based on size, i.e., the largest subspecies *F. a. naumanni* (hereafter “*naumanni*,” found in the High Arctic, such as Spitsbergen and northwestern Greenland), the intermediate-sized subspecies *F. a. arctica* (hereafter “*arctica*,” present in Norway, Iceland, and Faroe Islands), and the smallest subspecies *F. a. grabae* [hereafter “*grabae*,” found in France, British Isles, and southern Norway (*20*); see Fig. 1A]. Recent genomic analyses also identified a distinct Western Atlantic cluster (*19*). This genetic structure has been attributed to barriers to gene flow on different spatial scales arising from a complex set of ecological drivers, including the interplay between overwintering grounds, natal dispersal, and ocean regimes (*19*). In addition, in one population in Greenland (Thule), three genetically distinct clusters representing both *naumanni* and *arctica* co-occur during the breeding season, so far without any evidence for hybridization (*27*). In contrast, a hybrid population of *naumanni* and *arctica* was found on the Arctic Island of Bjørnøya [Norway; (*19*)]. The evolutionary history of this hybrid population, including the timing and direction of gene flow, remains unknown.

Here, we analyze whole-genome data of modern and historic Atlantic puffin specimens to provide detailed insights into the origin of the hybrid population on Bjørnøya. First, to optimize the analyses of historic and contemporary data, we assembled and annotated a chromosome-level reference genome and performed admixture and demographic analyses using whole-genome data of 18 modern individuals across three breeding colonies that represent the parental and hybrid populations. Second, we used genomic data from modern and historical specimens (dated from 1868 to 1910) to reconstruct the demographic history of the different subspecies of Atlantic puffins and obtain an accurate estimate of the timing of hybridization (*10*, *11*, *13*). Third, we investigated evidence for recent genetic impacts, which are of value for the management of species of conservation concern (*13*, *28*–*30*). Demographic reconstructions show that the different subspecies started diverging approximately 100,000 years ago, most likely due to climatic oscillations in the Pleistocene (*31*, *32*). Our results show that the origin of the hybrid population on Bjørnøya lies within the past 100 years, coinciding with the rapid 20th-century climate change in the Arctic. Furthermore, we find evidence for losses of genetic variation over the past few decades, which is important for the conservation of the species due to the potential, associated decline in population viability (*13*, *30*). Given that ecosystems worldwide are changing at unprecedented rates, our work highlights the power and importance of temporal genomics to detect and link population-scale genetic changes with environmental and anthropogenic stressors of the past few decades.

## RESULTS

### A chromosome-level reference genome of the Atlantic puffin

Building on and enriching a previous, publicly available reference genome assembled with 10x Genomics data (table S1) (*19*), a high-quality, chromosome-level reference genome of the Atlantic puffin was assembled into 24 autosomes, 2 sex chromosomes, 1 mitochondrial genome, and 1 “unplaced scaffolds” sequence (229 unplaced scaffolds merged into a single sequence with a length of 6.1 Mb), using PacBio (CLR, Continuous Long Read), 10x Genomics, and Hi-C sequencing data (see Materials and Methods and Supplementary Text). The assembly is 1.215 Gb long, with an N50 contig/scaffold length of 16 Mb/82 Mb, holds 97.4% of protein-coding sequences from the avian set of the OrthoDB v10 database (BUSCO score), and displays substantial synteny with the razorbill (*Alca torda*; fig. S1 and table S2). The assembly has a quality value score of 37.63, an error rate of 0.017%, and a k-mer completeness rate of over 90% (fig. S2 and table S2). We observe a bimodal distribution of 10x Genomics k-mer read data (fig. S2), which may be driven by diploidy where some k-mers occur in both haplotypes and some occur in just haplotype one. As the 10x Genomics data were used for polishing, the impact on the completeness and correctness of the assembly is expected to be negligible. The annotation based on whole-genome alignments with other avian species includes 15,523 protein-coding genes, with an average coding DNA sequence length of 2316 base pairs (bp) and an average intron length of 4088 bp (table S2). The mitochondrial genome is 19,084 bp long, which is 2 kb longer than the currently published *F. a. arctica* mitogenome, now containing a large pseudo-duplicated region that was previously not assembled (fig. S3). The inclusion of this region emphasizes the impact of long reads in improving the assembly of mitogenomes that were previously obtained with short reads only, especially given that repeats and duplications are inherent to mitogenomes (*33*).

### Genomic population structure and diversity

Atlantic puffins on Spitsbergen and Røst belong to distinct genomic clusters and subspecies, as previously identified from low-coverage whole-genome data (Fig. 1 and figs. S4 and S5) (*19*). Bjørnøya, by contrast, represents an admixed population where *arctica* and *naumanni* interbreed (Fig. 1 and figs. S4 and S5). Principal components analysis (PCA) and ADMIXTURE (*K* = 2) based on high-coverage resequencing of Spitsbergen, Røst, and Bjørnøya individuals support two main clusters with Bjørnøya forming an intermediate group (Fig. 1 and fig. S4). The presence of a hybridization zone on Bjørnøya and gene flow between *naumanni* and *arctica* (table S3) is confirmed by significantly negative *f3*-statistics (*Z* < −3) for the (Spitsbergen, Røst; Bjørnøya) topology, significantly positive *D* statistics caused by an excess of ABBA sites in the {[(Røst, Bjørnøya), Spitsbergen], Razorbill} topology, and an *f4*-ratio of 0.297 in this topology (table S3). Spitsbergen is most distinct from the two other populations with significantly higher between-colony *F*_ST_ values (Dunn test with Holm correction, *P* < 0.05; fig. S6 and table S4), significantly lower nucleotide diversity and heterozygosity (Dunn test with Holm correction, *P* < 0.05; figs. S6 and S7 and table S4) and positive Tajima’s *D* values. Bjørnøya and Røst have the lowest between-colony *F*_ST_ values, similar levels of nucleotide diversity and heterozygosity, and negative Tajima’s *D* values (figs. S6 and S7 and table S4).

Historical Røst and Spitsbergen museum specimens cluster with their respective modern counterparts (Fig. 1B), apart from two individuals from Spitsbergen that cluster with Røst (Fig. 1B and figs. S5, S8, and S9). These individuals were substantially smaller in size compared to other Atlantic puffins in Spitsbergen (fig. S10). At *K* = 2, which was the optimal model fit for the ngsAdmix analyses (fig. S11), both Røst and Spitsbergen show very similar ancestry proportions throughout time (Fig. 1B and fig. S8). In contrast, none of the historical Bjørnøya puffins cluster with their modern counterparts (Fig. 1B and fig. S5). All seven individuals cluster with the Røst samples in the PCA and admixture analysis and do not display ancestry proportions indicative of admixture (Fig. 1B and figs. S8 and S9).

**Fig. 2.**
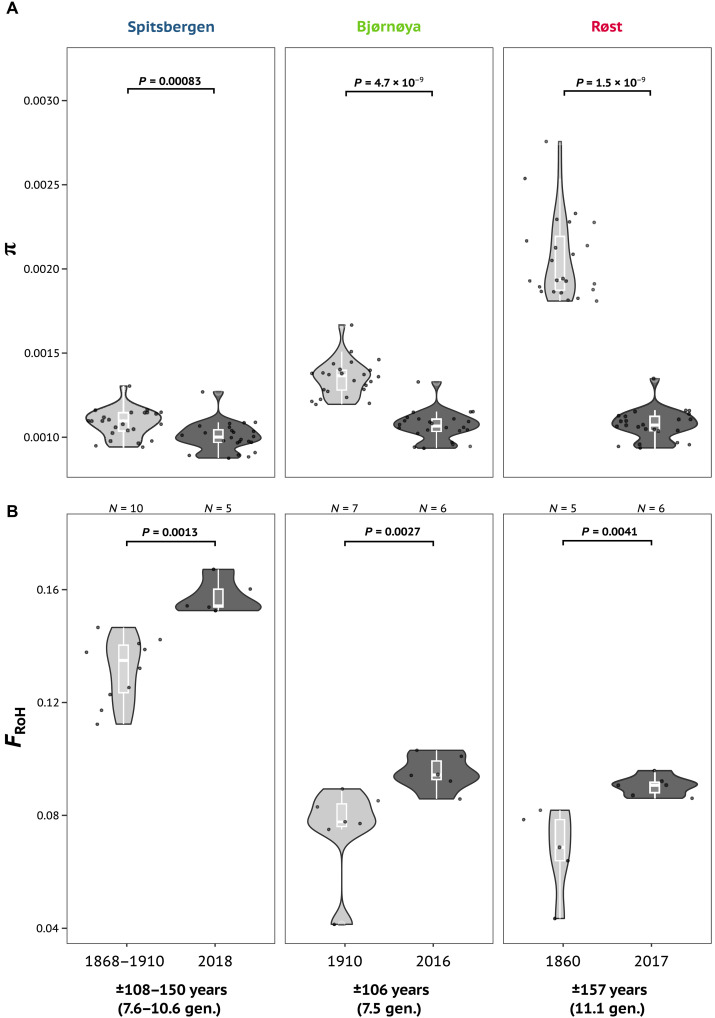
Temporal changes in genomic diversity across three Atlantic puffin populations. (**A**) Nucleotide diversity and (**B**) inbreeding (*F*_ROH_) were estimated for the historical and modern populations of Spitsbergen, Røst, and Bjørnøya representing the subspecies *F. a. naumanni*, *F. a. arctica*, and their hybrids, respectively. Nucleotide diversity for each population was calculated per chromosome (*N* = 24, black dots) with the program ANGSD using a site frequency spectrum. Individual inbreeding coefficients, *F*_RoH_, were defined as the fraction of the individual genomes falling into runs of homozygosity (RoHs) of a minimum length of 150 kbp (*19*). RoHs were declared as all regions with at least two subsequent 100-kbp windows harboring heterozygosity below 0.482 × 10^−3^. The *x* axes present sampling years and their differences in years and generations are given. The significance of differences is indicated by *P* values.

Throughout the past 108 to 157 years, Spitsbergen and Røst puffins have experienced a significant loss of genetic diversity (*W*_Spitsbergen_ = 135, *P =* 0.0008; *W*_Røst_ = 0, *P* = 1.5 × 10^−9^; Fig. 2A and tables S5 and S7) and increase in inbreeding (*W*_Spitsbergen_ = 41, *P* = 0.001; *W*_Røst_ = 30, *P* = 0.004; Fig. 2B and fig. S12). Historically, Røst had the highest genetic diversity characterized by a large number of rare alleles indicating a large population size (figs. S6 and S13 and table S6). Contemporary Spitsbergen and Røst individuals showed no significant differences in levels of heterozygosity or runs of homozygosity (RoH) lengths (figs. S14 and S15), yet significantly elevated Tajima’s *D* values (*W*_Spitsbergen_ = 576, *P* = 1.5 × 10^−9^; *W*_Røst_ = 576, *P* = 1.5 × 10^−9^; figs. S14 and S16 and table S5), compared to their historical conspecifics. Tajima’s *D* was positive in modern Spitsbergen individuals while negative in all other colonies independent of the sampling period (table S5). Concurrent with the changes in Spitsbergen and Røst, Bjørnøya shows a significant decrease in nucleotide diversity (*W*_Bjørnøya_ = 9, *P* = 4.7 × 10^−9^; Fig. 2A and table S5) and an increase in inbreeding (*W*_Bjørnøya_ = 41, *P* = 0.003; Fig. 2B and fig. S12) and Tajima’s *D* (*W*_Bjørnøya_ = 576, *P* = 1.5 × 10^−9^; figs. S14 and S16 and table S5) throughout 106 years. Moreover, the introgression of Spitsbergen ancestry into Bjørnøya has resulted in modern individuals on Bjørnøya having significantly longer RoHs than their historical conspecifics (*W*_Bjørnøya_ = 42, *P* = 0.0017; fig. S15).

### Reconstructing demographic history

**Fig. 3.**
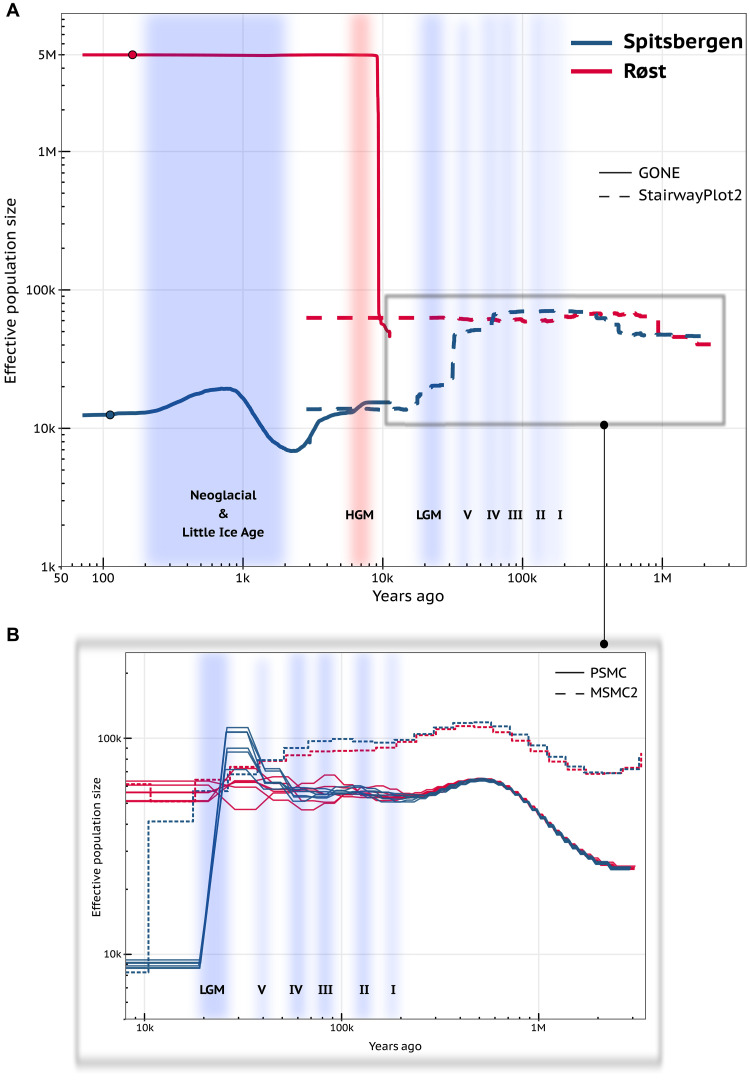
Temporal reconstruction of effective population size of two Atlantic puffin populations across the past 2 million years. (**A**) The demographic histories of the Spitsbergen (*F. a. naumanni*) and Røst (*F. a. arctica*) colonies throughout the past 2 million years were estimated with the program GONE (straight) and StairwayPlot2 (dashed). (**B**) Changes in the effective population size in the period spanning 10,000 to 2 million years ago were also determined with the programs PSMC and MSMC2. Red and blue periods represent climatic warm and cold periods, respectively. HGM, Arctic Holocene Glacial Minimum; LGM, Last Glacial Maximum. Periods I to V = regional and local cold periods on Spitsbergen reconstructed by multiple proxies (*31*).

We reconstructed demographic trajectories of effective population size (*N*_e_) for the Spitsbergen (*naumanni*) and Røst (*arctica*) colonies using a combination of methods that cover a range of temporal scales (Fig. 3). The common ancestral effective population size of *naumanni* and *arctica* was relatively stable between 100 thousand years (ka) and 500 ka ago at ~60,000 to 70,000 individuals (Fig. 3 and figs. S17 to S19) before both subspecies start to display independent trajectories between 40 ka and 100 ka ago (Fig. 3 and figs. S17 to S19). From around 15 ka to 40 ka ago, the demographic trajectories for *naumanni* and *arctica* diverged substantially, coinciding with the Last Glacial Maximum (LGM) and High Arctic, pre-LGM, Weichselian glacial cycles (Fig. 3 and figs. S17 to S19). The Røst population remained relatively constant in effective size with 50,000 to 60,000 individuals until 10 ka ago, whereas Spitsbergen decreased to about 8000 to 13,000 individuals (Fig. 3 and figs. S17 to S19). The timing of the split of *naumanni* from the ancestral *arctica* effective population is corroborated by the relative cross-coalescence rate, which falls below 1.0 100 ka ago and below 0.5 (populations separated) 15 ka ago (fig. S20). While trends in effective population size before 10 ka ago were consistently reconstructed across all methods, multiple sequentially Markovian coalescent (MSMC2) displayed substantially higher *N*_e_ of the ancestral (*naumanni*/*arctica*) population compared to the other methods (Fig. 3B and fig. S19), while pairwise sequentially Markovian coalescent (PSMC) showed a spike in effective population size of Spitsbergen 30 ka ago followed by a drop in the subsequent 10 ka, supporting the independence of the colony from the *arctica* population (Fig. 3B and fig. S18).

Within the past 10 ka, the Spitsbergen population remained relatively constant in effective size (10,000 to 11,000 individuals) for approximately 7 ka before dropping to 7000 individuals during the onset of the Neoglacial period followed by a threefold increase and subsequent decrease to ca. 12,000 individuals (Fig. 3A and fig. S21). Simultaneously, the Røst population increased in effective size from 50,000 to 5 million individuals at the onset of the Arctic Holocene Glacial Minimum (HGM; 6 ka to 8 ka ago) and has remained at this level ever since (Fig. 3A and fig. S21).

### Admixture timing of arctica and naumanni at Bjørnøya

**Fig. 4.**
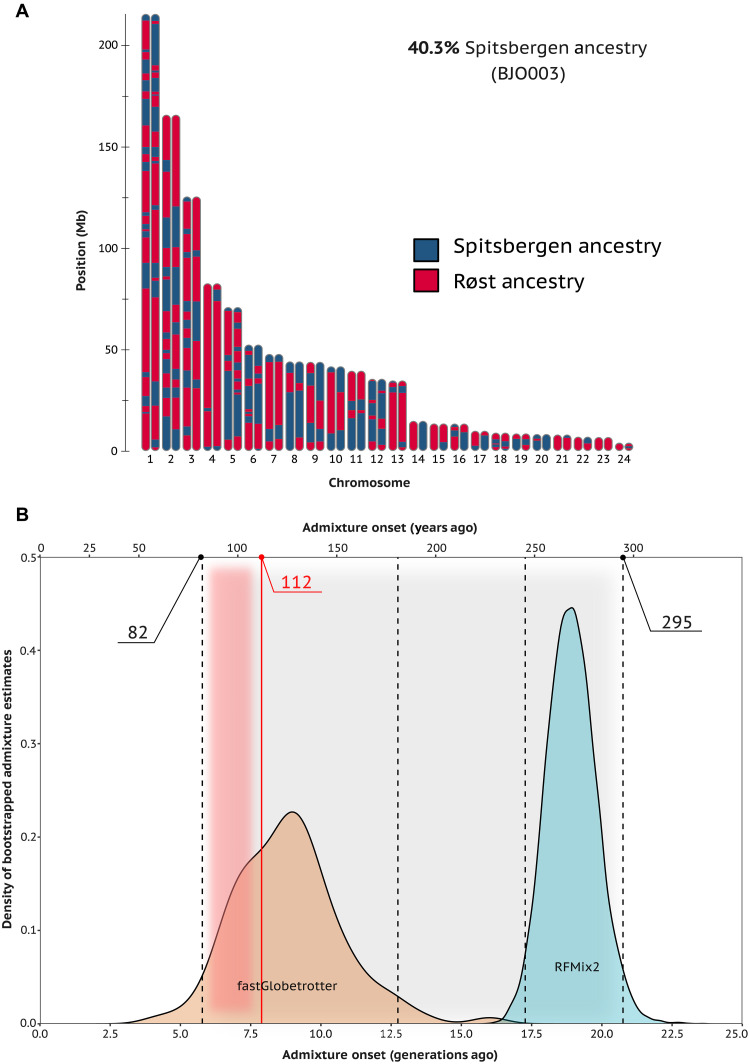
Estimation of the onset of admixture between two Atlantic puffin subspecies using genomic data. Length and distribution of genomic regions assigned with Spitsbergen (*F. a. naumanni*) ancestry across all 24 autosomes of six hybrid genomes from Bjørnøya, as depicted in (**A**), were used to determine the 95% confidence intervals of admixture onset estimates, as shown in (**B**). (A) The ideogram shows 48 haplotypes of one example hybrid individual reconstructed by the program RFMix2 (the ideograms of all hybrids are presented in fig. S22). (B) The onset of admixture in years ago (top axis) and the number of generations ago (bottom axis) were estimated using fastGlobetrotter and RFMix2. FastGlobetrotter uses ancestry probabilities determined by Chromopainter2. Numbers in black at the top (82 and 295) represent the lower and upper limit of the 95% confidence intervals of the two approaches. The number in red (112) presents the sampling year of the historical specimens on Bjørnøya. The shift of the distribution of admixture timing estimates by RFMix2 further to the past is likely due to an underestimation of the length of the introgressed tracts, which is caused by switch errors during phasing chunking up the tracts.

The onset of admixture between *arctica* and *naumanni* on Bjørnøya was timed using two different approaches that use local ancestry probabilities along the hybrid genomes (Fig. 4). *Naumanni* tracts that introgressed into *arctica* genomes on Bjørnøya, as determined by RFMix2, account for 7.2 to 48.9% of the hybrid genomes, which is similar to levels identified by the ADMIXTURE analysis (Fig. 4A and fig. S22). The length distribution of introgressed *naumanni* tracts is dominated by tracts with a length of 2 to 3 Mb and contains segments up to 60 Mb (fig. S23A). Combining all Bjørnøya individuals, and using the length and proportion of introgressed *naumanni* tracts per chromosome (fig. S23) and a chromosome-specific recombination rate (table S7), the most probable time of admixture onset on Bjørnøya was estimated to be 18.1 generations (272 years) ago (CI_95%_ = [17.3 to 20.8]; Fig. 4B). Similarly, individual-based admixture timings applying the same method range between 16.4 and 23.2 generations ago (fig. S23B). FastGlobetrotter uses local ancestry paintings of hybrid individuals generated by ChromopainterV2 and suggests a one-pulse over a two-pulse admixture scenario due to the better model fit (fig. S24 and table S8). The bootstrapped estimates of admixture timings from fastGlobetrotter predicted an onset of admixture 8.9 generations (126 years) ago (CI_95%_ = [5.8 to 12.8]; Fig. 4B and table S8). The 95% confidence intervals of the two approaches (RFMix2 versus fastGlobetrotter) span a combined period between 82 and 295 years ago overlapping with the first 30 years following the collection of our historical specimens (112 years ago; Fig. 4B).

## DISCUSSION

We provide genomic insights into the demographic history of the Atlantic puffin throughout the past 500,000 years, which is a period of marked phylogeographical separation between avian species (*34*). We show that the currently recognized subspecies *F. a. arctica* and *F. a. naumanni* diverged from a common ancestor at least 40,000 years ago. Despite this evolutionary divergence, a hybrid population on the Arctic island of Bjørnøya originated only very recently. The absence of historical Bjørnøya hybrids combined with the estimated recent onset of admixture (Fig. 4) implies that hybridization between *arctica* and *naumanni* must have been initiated at least after 1910. Although hybridized individuals have been observed in several Arctic species [e.g., polar bear (*14*) and beluga whale (*35*)], we provide the first genomic evidence for the recent establishment of an entire hybrid population driven by a southward distributional shift. Our findings present a rare observation of a population-scale response to the rapid environmental changes that the Arctic ecosystem has started experiencing within the past century (*1*, *7*, *36*, *37*).

Although the predominant distributional shift of flora and fauna in response to global warming is toward the poles (*1*–*3*), we here instead observe a southwards expansion of an Arctic subspecies. There may be two reasons for such an unexpected direction. First, *naumanni* colonies on Spitsbergen could have experienced favorable conditions in the past 100 years and the increase in population sizes could have led to the emigration of Atlantic puffins as a response to density-dependent factors (*20*). However, while there is no data on population trends of Atlantic puffin colonies on Spitsbergen within the past 100 years (*38*), the significant reduction in genetic diversity and increase in inbreeding over time (Fig. 2) do not support such an increase in population size. Alternatively, migration away from Spitsbergen could be attributed to detrimental environmental conditions at the natal colony, leading to Atlantic puffins establishing alternative colonies further south that may have been characterized by more favorable conditions (*20*, *39*). While fishery-induced food shortages or pollution are harmful factors that cannot be ruled out, the estimated onset of hybridization notably coincides with the beginning of the 20th-century Arctic warming, where sea-surface temperature increased by up to 1.5°C (*36*, *37*). This period is associated with large changes in plankton and fish communities and/or abundance (*40*) that are thought to have had detrimental effects on seabirds in the North Atlantic and the Arctic (*18*, *25*, *41*). Also, in part, driven by the multidecadal, cyclic pattern of ocean temperatures (*37*), the warming of the Arctic in the early 20th century likely played a substantial role in affecting the breeding success of puffins, as changes in sea temperatures can alter the food web and with that abundance and distribution of key prey for Atlantic puffins (*25*, *42*). For example, the distribution of age-1 herring, an important prey for puffins during chick rearing, shifted eastward from the western Barents Sea to the Murman Coast in 1920 (*26*). Regardless of the ecological driver, our observations show that the responses and distributional shifts of Arctic fauna to global warming are not unidirectional, and attempts to understand or predict faunal responses to the rapidly changing climate need to incorporate such ecological complexity.

One common mechanism explaining phylogeographic patterns in Arctic seabirds, such as the European shag [*Gulosus aristotelis*; (*43*)] or common murre [*Uria aalge*; (*44*)], is allopatric divergence through isolation into different refugia [reviewed in (*42*)]driven by multiple regional and local glacial periods with adaptation to different habitat and climatic conditions. Nevertheless, evidence for such allopatric divergence has been difficult to obtain, given that many seabirds have colonized the Arctic within the past 100,000 years, leading to shallow evolutionary genomic divergence (*19*, *45*, *46*). High-resolution genomic data are therefore essential to detect such recent divergence, yet such data remain scarce for seabirds. This lack of data greatly limits our ability to infer basic population structure, gene flow or demographic history, and the mechanisms that drive these patterns. Here, we observe that *naumanni* and *arctica* start diversifying from a common ancestor ~100 ka ago, representing the minimum age of divergence. This gradual separation of these subspecies throughout several Pleistocene glacial cycles (*31*) culminates in a complete differentiation during the LGM when *naumanni* may have inhabited a cryptic glacial refugium in the Arctic (*47*, *48*). At the same time, *arctica* was likely confined to lower latitudes, such as Iceland or the Faroes (*49*). The high genetic diversity observed among Atlantic puffins in this area suggests that these populations remained larger than the more confined *naumann*i (*19*, *47*). *Arctica* subsequently colonized the North Atlantic following the melting of the ice sheets during the Arctic HGM (*32*). The post-LGM radiation within the North Atlantic allowed this subspecies with colonies throughout Norway, Iceland, and the Faroe Islands—here represented by the colony on Røst—to reach its recent population size, while the Spitsbergen population remained multiple orders of magnitude smaller (Fig. 3). Such a process of divergence has been proposed for other Arctic seabirds (*43*, *45*, *50*, *51*), which, for puffins, is now supported by a detailed genomic reconstruction of such recent divergence based on high-quality genomic data. Herewith, we provide evidence for the defining influence environmental changes have had in shaping the demographic histories of an Arctic seabird.

Analyses of historical and ancient DNA enable a direct assessment of changes in genetic diversity over time and allow the quantification of genetic impacts that are highly relevant for conservation efforts (*11*, *13*, *28*–*30*). While small populations are generally thought to be at higher risk of extinction (*30*, *52*), it has been shown that the correlation between modern genome-wide diversity and population sizes of endangered species, for example, the tiger (*53*) or various apes (*54*), can be weak (*30*). Moreover, the relationship between genetic estimates of effective population size (*N*_e_) and census size (*N*) is complex and influenced by factors, such as gene flow and genomic population structure, which renders relative comparisons of *N*_e_ within a species more useful. We find that modern genomic data identified a large effective population size for the Atlantic puffin colony of Røst (effective population size, 5 million), likely reflecting high gene flow between several colonies across Norway, Iceland, and the Faroe Islands, which, together with Røst, comprise a single genomic cluster (*19*). Similarly, the census size at Røst (226,000 breeding pairs in 2022) is orders of magnitude larger than the total Spitsbergen population [<10,000 breeding pairs (*20*)]. Yet, comparing historical to modern specimens, we nonetheless detected a significant loss of genetic diversity and an increase in inbreeding across the Atlantic puffin colonies of Spitsbergen and Røst within the past 100 years. Such genomic erosion can lead to a loss of adaptive potential and reduced population viability and is concerning from a conservation standpoint (*13*, *30*, *55*). Our observations therefore underline the importance of temporal genomic sampling together with a high-quality reference genome (*56*) to evaluate the status of species that are subject to rapid ecological changes and are of conservation concern.

To date, concrete genetic evidence that inter- or intraspecies hybridization is occurring on a population scale as a result of recent climatic changes in the Arctic is rare. By using modern and historical genomic data, we show that the onset of admixture between two Atlantic puffin subspecies has occurred within the past 100 years providing an unprecedented scale, resolution, and accuracy for the timing of population-scale hybridization. Moreover, the emergence of this hybrid population coincides precisely with the anthropogenic warming of the Arctic. The unexpected southward range shift of a High Arctic subspecies thereby highlights how anthropogenic stressors can potentially force distributional changes resulting in hybridizing species or subspecies, essentially displacing genomic variation of a species whose populations have co-evolved over thousands of years (*4*, *57*). The hybridization of Atlantic puffins in the East Atlantic Arctic may be a forecast of future scenarios throughout other parts of the Arctic illustrated by the sympatry of genetically distinct, but non-admixing, Atlantic puffin subspecies within a single High Arctic colony on the west coast of Greenland (*27*). Both the timing of admixture, as well as genomic erosion across multiple Atlantic puffin colonies and subspecies, present another line of compelling evidence that the past 100 years have been tremendously impactful on Arctic communities and highlight the importance and power of analyzing and linking contemporary and historical genomic data.

## MATERIALS AND METHODS

### Reference genome assembly and annotation

For the construction of an improved and highly contiguous and complete de novo Atlantic puffin genome assembly, we used a combination of PacBio, 10x Genomics Chromium, and Hi-C sequencing data. In total, six SMRT cells, sequenced on the PacBio Sequel II platform in CLR mode, generated 3.98 million reads with an average length of 13 kbp (N50: 24 kbp) and a coverage of 43.7X. A single 10x Genomics Chromium library was sequenced on three Illumina HiSeqX lanes generating approximately 2 billion reads (500.4X coverage). A cross-linked chromatin interaction (Hi-C) library was built with the Arima Genomic Hi-C kit and sequenced on an Illumina NovaSeq sequencer generating ca. 120 million unique Hi-C reads (157.1X coverage) of which ca. 50 million fragments were longer than 20 kb. The initial assembly was performed following the recommendations from the Vertebrate Genomes Project (*58*) with some adjustments. We used Flye (*59*), Falcon-Unzip (*60*), and Canu (*61*) for the initial assemblies to find the strategy with the optimal performance for the Atlantic puffin genome (table S9). Subsequent refinement and manual curation of the initial assemblies followed published recommendations and pipelines (see, e.g., https://github.com/VGP/vgp-assembly/tree/master/pipeline, accessed December 2022), as performed for previous genome assemblies (*58*). We chose the best (highest % of complete BUSCO score, longest N50, fewest *N*’s per 100 kb) assembly for further refinement (table S9). First, using mitoVGP (*33*) in combination with the PacBio and 10x Genomics reads, the mitogenome was identified among all scaffolds and subsequently separated from the assembly. Subsequently, the nuclear scaffolding was improved with HiGlass (*62*) and PretextView (https://github.com/wtsi-hpag/PretextView, v0.2.5, accessed December 2022). The resulting 26 largest super-scaffolds were visually distinct in their length distribution (fig. S2) and designated as “chromosomes.” The remaining sequences were labeled as “unplaced scaffolds” and merged into a single sequence with 200 *N'*s in between. The final assembly consisted of 24 autosomes, 2 sex chromosomes, 1 mitogenome, and 1 merged-unplaced scaffold sequence. The mitochondrial genome was annotated with the MITOS2 (*63*) web server. The nuclear genome was annotated as in (*64*). Further details on the reference genome assembly and annotation steps can be found in Supplementary Text.

### Sequencing and single-nucleotide polymorphism identification of modern individuals

Samples from a total of 18 adult Atlantic puffin individuals collected across three breeding colonies (Spitsbergen, Bjørnøya, and Røst; Fig. 1) between 2012 and 2018 were sequenced for the present study to a genome-wide average depth of coverage of 21.6 (high-coverage dataset, >10x; data S1). These 18 individuals were sequenced and analyzed at a lower coverage for a previous whole-genome study of Atlantic puffins (*19*). These samples were collected and made available by SEAPOP (www.seapop.no/en), SEATRACK (www.seapop.no/en/seatrack/), and ARCTOX (www.arctox.cnrs.fr/en/home) using the appropriate permits for collection and approved by the appropriate ethics committees. Spitsbergen and Røst were selected as representative colonies of the subspecies *naumanni* and *arctica*, respectively, and Bjørnøya was previously identified as a hybrid population between the two (*19*). We processed sequencing reads and mapped them to the Atlantic puffin assembly in PALEOMIX v1.2.14 (*65*).

Genotypes at autosomal single-nucleotide polymorphisms (SNPs) were jointly called with GATK v4.2.0 (*66*) and subsequently filtered (see Supplementary Text). Removal of one (of two) related individuals from the SNP dataset and pruning of SNPs to account for linkage disequilibrium resulted in a total of three different SNP datasets (table S10). Two additional SNP panels containing nonvariant sites were produced by applying the flag “*--include-non-variant-sites*” in GATK *GenotypeGVCFs* (table S10). Last, three SNP datasets with an outgroup were built by mapping sequencing data of the razorbill (*A. torda*, GCA_008658365.1) to the Atlantic puffin reference and either calling genotypes with GATK followed by combining SNPs with the Atlantic puffin genotypes or sampling the consensus base for each individual (Atlantic puffins and razorbill) at each site with ANGSD v.0.935 (table S10) (*67*). A detailed description of laboratory methods and SNP calling can be found in Supplementary Text.

### Sequencing and genotype likelihood estimation of historical individuals

Samples from a total of 22 adult Atlantic puffins from the three breeding areas [Spitsbergen (*N* = 10), Bjørnøya (*N* = 7), Røst/Lofoten archipelago (*N* = 5)] dating from 1860 to 1910 were sequenced for this study to a genome-wide average depth of coverage of 5.7 (data S1). These areas were chosen to represent the historical counterparts to the analyzed modern individuals of the subspecies *naumanni*, *arctica*, and of the hybrid population. Two of the Spitsbergen and one of the Røst individuals were substantially smaller in size and had therefore been assigned to be “mutants” or “migrants” by the original authors and collectors of the specimens (fig. S10) (*68*). All historical samples were made available by natural history museums using the appropriate permits for collection and import and export, and approved by the appropriate ethics committees. Before sequencing, DNA was extracted following protocols customized for historical avian specimens, and libraries were built with the single-stranded Santa Cruz Reaction library protocol (*69*). Sequencing reads from the 22 historical specimens together with all 77 modern Atlantic puffins sequenced to date (data S1) (*19*, *27*) were processed in PALEOMIX.

After removing transitions and one related individual, an ANGSD pre-run determined filter settings (fig. S25), which were subsequently applied to calculate genotype likelihoods for SNPs covered in at least 95 of 98 individuals. The dataset was pruned to account for linkage disequilibrium (table S10). A detailed description of laboratory methods applied to historical specimens and genotype likelihood estimation can be found in Supplementary Text.

### Genomic population structure

The population structure identified in (*19*) was validated with the 18 high-coverage individuals. A PCA was conducted with SmartPCA (*70*). Individual ancestry proportions were estimated with ADMIXTURE v1.3.0 (*71*).

Investigation of the genomic population structure was expanded with the genotype likelihood panel including 76 medium-coverage (5× to 10×) modern and 22 historical samples (table S10). A PCA was performed with PCAngsd v0.982 (*72*) and individual ancestry proportions were estimated in ngsAdmix v32 (*73*). The fit of the number of ancestral populations to the dataset was evaluated with evalAdmix v0.95 (*74*). A thorough description of the genomic population structure methodology can be found in Supplementary Text.

### Genomic differentiation, diversity, and inbreeding

Genomic differentiation and diversity between and within the three Atlantic puffin breeding colonies at Spitsbergen, Bjørnøya, and Røst were assessed from the high-coverage dataset. Heterozygosity was quantified with VCFtools v.0.1.16 (*75*); Tajima’s *D*, π, and *F*_ST_ were calculated as the average of all 50-kb sliding window values calculated with the *tajima* function of the utility program VCF-kit (https://vcf-kit.readthedocs.io/en/latest/, accessed December 2022) and the script *popgenWindows.py* (https://github.com/simonhmartin/genomics_general, accessed December 2022). Levels of heterozygosity in modern Bjørnøya individuals were validated using the theoretical relationship between heterozygosity, *F*_ST_, ancestry fraction, and heterozygosity of the parental populations (fig. S26) (*76*).

Genomic differentiation, diversity, and inbreeding across the entire Atlantic puffin distribution and between modern and historical specimens were analyzed from the medium-coverage dataset. Using an unpruned set of sites without transitions, covered in at least 95 individuals and passing several additional quality filters without removing rare alleles, we generated folded one-dimensional (1D) site frequency spectra (SFS) for each population and genomic cluster with ANGSD and winsfs v0.6.0 (*77*). These were used to compute Tajima’s *D* and π per chromosome for each entity. We estimated per-individual, folded, 1D-SFS with ANGSD and winsfs with the same set of sites. Heterozygosity was calculated by dividing the number of polymorphic sites by the number of total sites present in the SFS. The proportion of runs of homozygosity (RoH) within each individual Atlantic puffin genome was computed as in (*19*). The cutoff for a “low heterozygosity region” was set to 0.482 × 10^−3^ (fig. S27). Individual inbreeding coefficients, *F*_RoH_, were also calculated as in (*19*). More details on the assessment of genomic differentiation, diversity, and inbreeding can be found in Supplementary Text.

### Demographic history

Before reconstructing the demographic history of the two Atlantic puffin subspecies, we generated estimates of the mutation rate of the Atlantic puffin genome. The mutation rate was calculated by (i) counting the number of polymorphic sites versus total sites after pseudo-haploidization (*78*) and (ii) transforming pairwise genetic distances obtained via identity-by-state (IBS) sampling [see, e.g., (*79*)]. IBS sampling identified the consensus base at each of the 343,369,191 filtered sites (table S10) to convert each sample including the outgroup to a pseudo-haploid genome. For both approaches, the razorbill genome was used as an outgroup combined with an approximated divergence time between the razorbill and Atlantic puffin of 34 million years ago (*80*) and a generation time of the Atlantic puffin of 14.2 years (*81*). We used a final generational mutation rate of 1.7125 × 10^−8^ for all subsequent analyses, which was the mean of the mutation rates estimated by both approaches.

The demographic histories of the Spitsbergen and Røst populations were reconstructed using four programs with different temporal resolutions and different utilization of genomic signals. For the PSMC (*82*), a consensus fastq file was generated for each of the 12 Atlantic puffins, filtering out sites with a depth of less than 10 or more than double the mean coverage of that Atlantic puffin and with a root mean square mapping quality of reads covering the site under 25 (*82*, *83*). After converting the fastq file to a PSMC input file and confirming the suitability of parameters and settings for the data (*83*), PSMC was run with “-N30 -t5 -r5 -p 4+30*2+4+6+10” for all individuals, including 100 subsequent bootstrap runs for each individual (*82*).

Before performing the StairwayPlot2 (*84*) analyses, a folded population-based SFS was generated by splitting the “NonVariant” dataset into populations and running the *easySFS.py* script (https://github.com/isaacovercast/easySFS, v0.0.1, accessed December 2022). StairwayPlot2 was run on each SFS with default settings, a mutation rate of 1.7125 × 10^−8^ and a generation time of 14.2 years (*81*).

MSMC2 (*85*) analyses were performed with phased SNP data. To phase the genomes, phase sets were identified in each individual Atlantic puffin genome by WhatsHap v0.18 (*86*) and genomes were subsequently statistically phased with Shapeit4 v4.1 (table S11) (*87*). After producing mappability files for each chromosome, individual per-chromosome calling mask files and individual MSMC2-specific vcf files were created. All samples were combined into specific MSMC2 per-chromosome input files. MSMC2 was run with “-s -p 1*2+22*1+1*2+1*6” for all unique combinations of haplotypes within each population as well as for unique combinations of two (out of five) different individuals from Spitsbergen versus two (out of six) different individuals from Røst, estimating within and across population coalescence rates (*85*).

As opposed to the other three tools, the software package GONE (*88*) is able to estimate fluctuations in *N*_e_ in the very recent past. Before running GONE, all chromosomes were randomly downsampled to a maximum of 500,000 SNPs per chromosome for computational purposes. GONE was run per population with default settings (*88*) and a mean genome-wide recombination rate of 1.63 centimorgan (cM)/Mb (table S7). The results of 100 bootstrap runs provided a median estimate of *N*_e_ and 95% confidence intervals.

Fluctuations in *N*_e_ reconstructed by either method were plotted using the mutation rate of 1.7125 × 10^−8^ and a generation time of 14.2 years (*81*). Each population trajectory was offset by the year the sampling took place (2016–2018; data S1). Last, major past warming and cooling periods on Svalbard and in the North Atlantic [see, e.g., (*31*, *32*)] were added to the resulting plots. A more detailed description of the analyses used for the reconstruction of demographic history can be found in Supplementary Text.

### Admixture validation and timing estimate

Corroborating the detection of admixture on Bjørnøya by previous research (*19*), *f3*-statistics, *D* statistics, and the *f4*-ratio were calculated. *f3*-statistics were determined with *threepop* in Treemix v1.13 (*89*) investigating the (P1 and P2; Admixed Pop) topology. The *D* statistics and the *f4*-ratio were calculated for the most significant (the highest proportion ABBA over BABA sites) combination of Spitsbergen, Bjørnøya, and Røst in the topology {[(P1, P2), P3], Outgroup} with the program Dsuite (*90*).

Two different approaches requiring phased genomes (table S10) were applied to estimate the time or onset of admixture between the two subspecies on Bjørnøya. The first approach used RFmix v2 (*91*) and the theoretical relationship between the length of introgressed tracts, time of admixture, recombination rate, and admixture fractions (*92*). The second approach combined Chromopainter2 (*93*) and fastGlobetrotter (*94*), which exploits the fact that the probability of inheriting two DNA segments from the same ancestral source along the genome of an admixed individual decays exponentially with a rate proportional to the date of admixture and genetic distance between the segments (*94*).

RFMix v2 was run on all Bjørnøya individuals per chromosome accounting for the fact that reference haplotypes (Spitsbergen and Røst) may not be of “pure” ancestry. Following previous research [see, e.g., (*95*)], the length of introgressed Spitsbergen tracts (*L*) and global diploid Spitsbergen ancestry estimates (*f*) in Bjørnøya individuals generated by RFMix, together with the chromosome-specific recombination rate (*r* in morgans per base pair; table S7) were supplied to the equation *L* = [(1 − *f*)*r*(*T*_ADMIX_ − 1)]^−1^ (*92*) to estimate the individual-based and chromosome-specific age of admixture. The distribution of *T*_ADMIX_ across all 24 chromosomes and six individuals was bootstrapped 10,000 times to identify the average time of admixture and the 95% confidence intervals [see, e.g., (*95*)]. For the second approach, the genomes of the Bjørnøya individuals were “painted” (i.e., local ancestry probabilities for each SNP per haplotype were determined) with Chromopainter2. FastGlobetrotter was run with the painted genomes, default settings, and chromosome-specific recombination rates (table S7). One hundred bootstrap replicates of the proposed admixture scenario (here: one-pulse) were combined to determine 95% confidence intervals for the estimated admixture timings. More details on the validation and timing of admixture can be found in Supplementary Text.

### Ethical statement

Feather, blood, and toepad samples of puffins included in this study were collected and handled under the following permits.

1. Gåsøyane, Røst, Hornøya, Bjørnøya (Norway): FOTS ID #15602 and #15603 from the Norwegian Food Safety Authority for SEATRACK and SEAPOP; Permit 2018/607 from Miljødirektoratet (Norwegian Environment Agency), dated 4 May 2018.

2. Gannet and Gull Island (Canada): Canadian Wildlife Service Migratory Bird Banding Permit 10559 G, approved Animal Use Protocol (AUP) by Eastern Wildlife Animal Care Committee (17GR01 and 18GR01), Newfoundland and Labrador Wilderness and Ecological Reserves Permit - Scientific Research (DOC/2017/02003), Canadian Wildlife Service Scientific Permit ST2785 (to M.L.M.), Canadian Wildlife Service Banding Permit 10694, and Acadia University Animal Care Committee Permits ACC 02-15 and 06-15 (to M.L.M.).

3. Isle of May (Scotland): Scottish Natural Heritage licence 2014/MON/RP/156 and Ringing Permit A400 (to M.P.H.).

4. Vestmannaeyjar, Papey, Breiðafjörður, Grímsey (Iceland): Icelandic puffins were legally hunted during the hunting period of 1 July to 15 August.

5. Faroe: Feathers came from predated birds collected in the field after the predator was finished with them.

6. Thule: All research in Greenland was conducted after ethical approval and issuance of permits by the Government of Greenland, Department of Fisheries, Hunting and Agriculture (High Arctic Institute permit numbers: Sags nr. 2012–065141, Dok. nr. 888887, Sags nr. 2013–083369, Dok. nr. 1204884, Sags nr. 2014–099682, Dok nr. 1594176, and Sags nr. 2015–115204, Dok. nr. 1975643).

7. Historical specimens: All specimens were sampled by the respective museums following their ethical guidelines and loan policies for tissue samples.

## Supplementary Materials

### This PDF file includes:

Supplementary Text Figs. S1 to S27 Tables S1 to S11 References
