# Supplementary material for: Hybridization of Atlantic puffins in the Arctic coincides with 20th-century climate change
Source: Sci Adv. 2023 Oct 6;9(40):eadh1407. doi: 10.1126/sciadv.adh1407 (PMC10558128; doi:10.1126/sciadv.adh1407)
Supplement: Supplementary file 1 — Supplementary Text Figs. S1 to S27 Tables S1 to S11 References [file sciadv.adh1407_sm.pdf]

Science Advances
AAAS

Supplementary Materials for

Hybridization of Atlantic puffins in the Arctic coincides with 20th-century climate change

Oliver Kersten *et al.*

Corresponding author: Oliver Kersten, oliver.kersten@ibv.uio.no; Sanne Boessenkool, sanne.boessenkool@ibv.uio.no

Sci. Adv. **9**, eadh1407 (2023)
DOI: 10.1126/sciadv.adh1407

The PDF file includes:

Supplementary Text
Figs. S1 to S27
Tables S1 to S11
References

Other Supplementary Material for this manuscript includes the following:

Data S1

Science Advances

AAAS

Supplementary Materials for

Hybridization of Atlantic puffins in the Arctic coincides with 20th-century climate change

Oliver Kersten *et al.*

* *Corresponding authors. Email: oliver.kersten@ibv.uio.no, sanne.boessenkool@ibv.uio.no*

This PDF file includes:

Supplementary Text
Figs. S1 to S27
Tables S1 to S11

Other Supplementary Materials for this manuscript include the following:

Data S1

Supplementary Text

1. Reference Genome Assembly & Annotation

1.1 DNA Extraction and Sequencing

For the construction of an improved and highly contiguous and complete *de novo* Atlantic puffin genome assembly, we used a combination of PacBio, 10x Genomics Chromium and Hi-C sequencing data. The DNA required for each technology was extracted from a fresh blood sample, which was collected on 13 June 2018 from a female Atlantic puffin (ring no.: MA28445, Zool. Museum Oslo) that has been breeding on Hernyken (67°25'33''N 11°52'50''E), Røst, northern Norway, each year since 2014 and until at least 2021.

High molecular weight (HMW) DNA for PacBio sequencing libraries was extracted from 10 µl of whole blood using the Circulomics Nanobind CBB Big DNA kit following the manufacturer's protocol for "Whole Blood - HMW". The library was prepared using the Pacific Biosciences Express library preparation protocol without any fragmentation of the sample prior to library preparation. Size selection of the final library was performed with BluePippin using a 15 kb cut-off. In total, six SMRT cells were sequenced on the PacBio Sequel II platform in CLR mode (Continuous Long Read) using Sequel Polymerase v3.0 and Sequencing chemistry v3.0. The sequencing generated 3.98 million reads with an average length of 13kbp (N50: 24kbp) and a maximum subread length of 180kpb. Assuming a genome size of 1.2Gbp the calculated coverage of the CLR reads was 43.7X. The sequencing service was provided by the Norwegian Sequencing Centre (www.sequencing.uio.no), Oslo, Norway.

HMW DNA for 10x Genomics Chromium was extracted from 15 µl of blood using the Kingfisher Cell and Tissue DNA Kit following the manufacturer's protocol. Subsequently, a single 10x Genomics Chromium library was built, which was sequenced on three Illumina HiSeqX lanes (150 bp insert size) at the SciLifeLab in Stockholm, Sweden. Each lane generated ~600-760 million paired-end reads for a total of approximately two billion reads (500.4X coverage).

A cross-linked chromatin interaction (Hi-C) library was built with the Arima Genomic Hi-C kit using 25-50 µl of blood preserved in ethanol. The library was pooled with others on a quarter S4 flow cell utilizing the 2x150 bp paired-end mode on an Illumina Novaseq sequencer at the Norwegian Sequencing Centre (www.sequencing.uio.no), Oslo, Norway. This generated ca. 120 million unique Hi-C reads (157.1X coverage) of which ca. 50 million were longer than 20 kb.

1.2 Initial Assembly & Polishing

Assembly was performed following the recommendations from the Vertebrate Genomes Project (*58*) with some adjustments. We used three different programs for the initial assemblies to find the strategy with the optimal performance for the puffin genome. The assembly programs used were Falcon-Unzip (*60*), Canu (*61*), and Flye (*59*). When possible, a combination of data was used as input (i.e. PacBio CLR was used in combination with Hi-C data in Canu and Falcon-Unzip). A combination of total assembly size and N50/L50 values was used to assess the initial assemblies (see Table S9). All three assemblies were very similar in total length (1.23 Gbp – 1.36 Gbp). The Flye assembly had the longest single contig (122 Mbp, compared to 68 Mbp from Falcon-Unzip and 60 Mbp from Canu), as well as the highest N50 (35 Mbp).

Continuing only with the Flye- and Falcon-based assemblies (Table S10), the 10X Genomics linked reads were aligned to the contigs of the initial assemblies and an adjacency matrix was computed from the barcodes using Scaff10X v2.1 (https://github.com/wtsi-hpag/Scaff10X, accessed December 2022). A gap of 100 N’s was inserted between linked contigs. Hi-C reads were mapped to the refined assemblies with BWA-mem (*96*) and filtered with Matlock (https://github.com/phasegenomics/matlock, accessed December 2022), which removes low-quality mapped reads and filters potential chimeric mapped reads. The cleaned and filtered Hi-C mapped reads were used for scaffolding with either ALLHiC (*97*) or Salsa2 (*98)* (Table S10). The final scaffolds were separated into haplotigs with Purge_dups (*99*). Subsequently, the assemblies went through manual curation to minimize the impact of errors produced by the assembly and scaffolding procedures. To perform the manual curation, Hi-C data was mapped to the primary haplotigs of the final assembly and visualized in Juicebox (*100*). Any mis-joined contigs were corrected either by rearranging or breaking contigs. Finally, the consensus sequence was improved and gaps were closed with Arrow (SMRT Analysis Software v7) which aligns PacBio CLR reads to the scaffolds using pbalign. The closed scaffolds were then polished with the 10X linked reads by two rounds of Longranger 2.2.2 (*101*), and Freebayes v1.2.0 (*102*), thereby polishing base-pairs and correcting homozygous mismatches (variants).

1.3 Assembly Refinement & Curation

Further refinement and curation of the four assemblies (Table S10) consisted of several downstream steps, following published recommendations (*103*) and pipelines (see e.g.

https://github.com/VGP/vgp-assembly/tree/master/pipeline, accessed December 2022) as performed in previous genome assembly curations (*58*). The curation primarily involved identifying and correcting regions of excess or low coverage indicative of a misassembly and detecting regions that are not supported by all three sequencing data types, and was done separately for each assembly.

First, using mitoVGP (*33*) in combination with the PacBio and 10x Genomics reads, the mitogenome was identified among all scaffolds and subsequently separated from the assembly. Leading and trailing N's were removed from each scaffold using custom scripts and falsely duplicated scaffolds were purged with purge_dups (*99*). Subsequently, Kraken2 (*104*) and the BlobToolKit (*105*) pipeline, including the program blobtools v2.6.1 (*105*), were used to flag potential contaminant sequences. Scaffolds flagged as "non-eukaryotic" by both programs were discarded to improve the signal to noise ratio for subsequent refinement steps. Telomeric ends were identified on each scaffold (https://github.com/VGP/vgp-assembly/tree/master/pipeline/telomere/telomere_analysis.sh, accessed December 2022) and repeats were masked across the assembly with RepeatMasker v.4.0.9 (developed by A.F.A. Smit, R. Hubley, and P. Green; see http://www.repeatmasker.org/, accessed December 2022) using the chicken as reference species. Finally, correctness of the refined assembly was examined and missassemblies were identified using the program Asset (*106*), which uses sequencing data from the three platforms (PacBio, 10X, Hi-C) to accumulate support evidence for a *de novo* assembly. Neither of the two available approaches of using Asset (Approach 1: https://github.com/dfguan/asset - accessed December 2022 - & Approach 2: https://github.com/VGP/vgp-assembly/tree/master/pipeline/asset - accessed December 2022) detected any low support (supported by only 1 of 3 sequencing platforms) for regions > 100kb in any of the assemblies. Completeness and continuity of the assemblies were assessed with BUSCO v5.0.0 (*107*) using the avian set of the OrthoDB v10 database (8338 gene groups) and with QUAST v5.0.2 (*108*), and the best (highest % of complete BUSCO score, longest N50, fewest N's per 100kb) assembly was chosen for further refinement (Table S10).

Scaffolds that were not joined into super-scaffolds in the best assembly but part of super-scaffolds in the other three assemblies were identified with SyRI (*109*). Subsequently, they were super-scaffolded manually with HiGlass (*62*) and PretextView (https://github.com/wtsi-hpag/PretextView, v0.2.5, accessed December 2022). The resulting 26 largest super-scaffolds were visually distinct in their length distribution and designated as "chromosomes", while the remaining sequences were labeled as "unplaced scaffolds" (Fig. S2). Additionally, all but one unplaced scaffold that harbored a complete BUSCO gene was placed and scaffolded into the 26 chromosomes using HiGlass and PretextView. The remaining unplaced scaffolds were merged with 200 'Ns' in-between to enable and streamline mapping with pipelines such as PALEOMIX (*65*), as they perform poorly upon provision of 100s-1000s of scaffolds (here 229). Sex chromosomes were identified with D-GENIES (*110*) via synteny with the razorbill (*Alca torda* - NCBI: bAlcTor1 primary, GCA_008658365.1), common tern (*Sterna hirundo* - NCBI: bSteHir1 primary, GCA_009819605.1) and European golden plover (*Pluvialis apricaria* - NCBI: bPluApr1 primary, GCA_017639485.1). The final assembly consisted of 24 autosomes, 2 sex chromosomes, 1 mitogenome and 1 unplaced scaffolds sequence. Completeness and correctness of the final assembly was further assessed using Merqury (*111*) and gfastats (*112*).

1.4 Assembly Annotation

The mitochondrial genome was annotated with the MITOS2 (*63*) web server using the protein prediction method of Al Arab (*113*).

The nuclear genome was annotated as in Sætre et al. (*64*). Using a repeat library provided by Alexander Suh called bird_library_25Oct2020 and described in Peona et al. (*114*), repeats were soft-masked in the puffin genome assembly and additional soft-masked genome assemblies for golden eagle (*Aquila chrysaetos*), chicken (*Gallus gallus*), great tit (*Parus major*), Anna's hummingbird (*Calypte anna*), zebra finch (*Taeniopygia guttata*), hooded crow (*Corvus cornix cornix*), lesser kestrel (*Falco naumanni*), mallard (*Anas platyrhynchos*), New Caledonian crow (*Corvus moneduloides*) and kākāpō (*Strigops habroptila*) were downloaded from NCBI. The triangle subcommand from Mash v. 2.3 (*115*) was used to estimate a lower-triangular distance matrix, and a Python script (https://github.com/marbl/Mash/issues/9#issuecomment-509837201 , last accessed September 21, 2022) was used to convert the distance matrix into a full matrix. The full matrix was used as input to RapidNJ v. 2.3.2 (*116*) to create a guide tree based on the neighbor-joining method. Cactus v. 2.0.4 (*117*) was run with the guide tree and the soft-masked genome assemblies (puffin and other species from NCBI) as input.

The gene annotation for the chicken was downloaded from NCBI and, together with the hierarchical alignment format file from Cactus, supplied as input to the Comparative Annotation Toolkit (CAT) v. 2.0 (*118*). The chicken was used as reference genome, the puffin as the target genome and the AUGUSTUS (*119*) species parameter was set to "chicken." InterProScan v. 5.47-82 (*120*) was run on the predicted proteins as found by CAT to find functional annotations. DIAMOND v. 2.0.7 (*121*) was used to compare the predicted proteins against the UniProtKB/Swiss-Prot release 2021_03 (*122*). AGAT v. 0.5.1 (*123*) added functional annotations from InterProScan and gene names from UniProtKB/Swiss-Prot. BUSCO v. 5.0.0 was run to assess the completeness of the annotation.

2. Modern Genomic Analyses

2.1 Sampling, Sequencing and Data Processing

Samples from a total of 18 puffins collected across three breeding colonies were made available for the present study by SEAPOP (http://www.seapop.no/en, accessed December 2022) and SEATRACK (http://www.seapop.no/en/seatrack/, accessed December 2022) (Supplementary Data File 1). Spitsbergen was selected as representative colony of the Atlantic puffin subspecies *Fratercula arctica naumanni*, Røst as representative colony of the subspecies *F. a. arctica*, and Bjørnøya was previously identified as a hybrid population between the two subspecies (*19*). The samples had been collected between 2012-2018 and consisted of blood preserved in EtOH or lysis buffer, or feathers. DNA from these samples was extracted as described in Kersten et al. (*19*).

The 18 genomic libraries, which were initially built for and part of the analysis of Kersten et al. (*19*), were resequenced across four lanes on an Illumina HiSeq4000 (Supplementary Data S1). Sequencing reads were processed in PALEOMIX v1.2.14 (*65*). Specifically, after removing adapters from forward and reverse reads with AdapterRemoval v2.3.1 (*124*) (*--mm3 --minlength25 --collapse yes --trimns yes --trimqualities yes*), reads were mapped to the new Atlantic puffin assembly using BWA *mem* v0.7.17 (*96*). Reads that aligned with a quality score (MapQ) of $\geq$ 25 were kept for duplicate removal with

PicardTools v2.18.27 (*125*) and indel realignment using GATKs *IndelRealigner (66)*. Finally, bam files were split into nuclear and mitochondrial bam files using SAMtools v1.9 (*126*).

2.2 SNP-based analyses

2.2.1 SNP calling and filtering

Genotypes at autosomal single nucleotide polymorphisms (SNPs) were jointly called with GATK v4.2.0 (*66*) by using the *HaplotypeCaller*, *GenomicsDBImport* and *GenotypeGVCFs* tool. Genotypes were filtered with BCFtools v1.9 (*126*) by applying *"--SnpGap 10 -e 'QD < 2.0 || MQ < 40 || FS > 60.0 || SOR > 3 || 12.5 > MQRankSum < -12.5 || 8> ReadPosRankSum < -8.0'"* according to GATKs Best Practices (*127*) and genotypes with a read depth less than 3 or a quality less than 15 were set as missing. Using BCFtools v1.9 and VCFtools v.0.1.16 (*75*), indels and non-biallelic SNPs were removed and only sites with a minimum quality of 30, global depth of 250-500X and a mean depth of 10.8-43.2X (0.5 and 2x of overall mean depth) across the 18 samples were kept. Finally, only SNPs present in all individuals were retained for subsequent analyses.

2.2.2 Linkage disequilibrium (LD) pruning & Relatedness

The SNP dataset was further pruned to account for linkage disequilibrium. Linkage expressed as the r^2 value was calculated for pairs of sites within 50 kb sliding windows with PLINK v1.9. Subsequently, linked sites ($r^2 > 0.2$) within 50kb of each other were greedily pruned with PLINK v1.9 (*-indep-pairwise 50 1 0.2*) until the remaining sites were in linkage equilibrium with each other. Within the resulting SNP dataset, relatedness between individuals was investigated with VCFtools v.0.1.16 (*--relatedness2*), which uses the KING inference (*128*) to find probabilities of relatedness. An estimated kinship coefficient range >0.354, 0.177-0.354, 0.0884-0.177 and 0.0442-0.0884 corresponds to duplicate/MZ twin, 1st-degree, 2nd-degree, and 3rd-degree relationships, respectively (*128*).

After detecting two related (2nd-degree) individuals on Spitsbergen (SPI002 and SPI015), SPI002 was removed from the raw, unfiltered and unpruned SNP dataset as it had the lower overall depth of coverage across the genome (20.04X vs. 21.59X, Supplementary Data S1). Subsequently, all SNP filtering and LD pruning was repeated as detailed above (note: some filter values changed, e.g. mean depth of 10.9-43.3X), resulting in a total of 4 SNP datasets, i.e. "LD-pruned" (18 individuals, 854,322 sites), "LD-Pruned/NoRelatedInd" (17 ind., 795,275 sites), "NonLD-pruned" (18 ind., 10,031,605 sites), and "NonLD-pruned/NoRelatedInd" (17 ind., 9,907,905 sites) (Table S10).

2.2.3 Non-variant sites and outgroup

In addition to the four SNP datasets generated above, two SNP panels containing non-variant sites and three with an outgroup were produced. Applying the flag "*--include-non-variant-sites*" in GATK *GenotypeGVCFs* and the same filters outlined above (see: SNP calling and filtering), "NonVariant/NoRelatedInd" (17 ind., 1,000,327,483 sites) and "NonVariant" (18 ind., 998,750,827 sites) datasets were generated (Table S10).

In order to be able to add an outgroup to several downstream analyses in this study, raw 10xGenomics sequencing data used for the assembly of the razorbill genome (*Alca torda*, GCA_008658365.1) was mapped to the new Atlantic Puffin reference assembly. Subsequently, razorbill SNPs were called with GATK v4.2.0 by using the *HaplotypeCaller* and *GenotypeGVCFs* tool. Only SNPs at sites present in the "NonLD-pruned/NoRelatedInd"

SNP dataset were retained and combined with this dataset while only keeping biallelic sites and positions with no missing genotype ("NonLD-pruned/NoRelatedInd/Outgroup" dataset). Similarly, SNPs at positions found in the "NonVariant" dataset were kept and combined in the same manner ("NonVariant/Outgroup" dataset). Finally, the consensus base (*-doIBS 2)* at 343,369,191 filtered sites (*"-uniqueOnly 1 -remove_bads 1 -minMapQ 30 -minQ 30 -C 50 -baq 2 -checkBamHeaders 1 -minInd 19 -setMinDepthInd 3 -setMaxDepthInd 80"*) was identified with ANGSD v.0.935 (*67*) and used to convert each sample including the outgroup to a pseudo-haploid genome ("IBS/Outgroup" dataset)(Table S10).

2.2.4 Genomic population structure

Population structure was investigated with the "LD-Pruned/NoRelatedInd" dataset. *SmartPCA* (*70*) of the Eigensoft package was run using "*outliermode: 2, altnormstyle: yes, lsqproject: yes*", followed by plotting the eigenvectors and eigenvalues of the significant principal components as revealed by the Tracy-Widom statistics.

Individual ancestry proportions were estimated using a maximum likelihood (ML) approach implemented in ADMIXTURE v1.3.0 (*71*) by setting the number of ancestral populations, K, from 1 to 6 and conducting 50 replicate runs for each K. The runs were clustered after similarity for each K and ancestry proportions were averaged within the major cluster using CLUMPAK (*129*) with default settings. The optimal value of K was chosen based on the method of Evanno (*130*) and biological validity.

2.2.5 Genomic differentiation, diversity and inbreeding

Estimates of F_{ST}, individual heterozygosity, Tajima's D and nucleotide diversity (**π**) were calculated with the "NonVariant/NoRelatedInd" dataset. Heterozygosity was quantified by using the *--het* flag with VCFtools. Tajima's D was calculated in 50 kb sliding windows (25 kb slide) with the *tajima* function (vk tajima 50,000 25,000) of the utility program VCF-kit (https://vcf-kit.readthedocs.io/en/latest/, accessed December 2022). F_{ST} and nucleotide diversity (**π**) were calculated in 50 kb sliding windows (25 kb slide) with the script *popgenWindows.py* (-w 50000 -m 10 -s 25000; written by Simon Martin - https://github.com/simonhmartin/genomics_general, accessed December 2022). Levels of heterozygosity in modern Bjørnøya individuals were validated using the theoretical relationship between heterozygosity, F_{ST}, ancestry fraction, and heterozygosity of the parental populations (Fig. S26) (*76*).

2.2.6 Mutation rate estimation

The mutation rate of the Atlantic puffin genome needed (as raw input or for plotting purposes) for the programs used to reconstruct the demographic history of the three puffin populations (see below) was estimated by two different approaches. It was estimated by a) counting the number of polymorphic sites vs. total sites (*78*) and b) transforming pairwise genetic distances obtained via identity-by-state (IBS) sampling (*79*, *131*). For both approaches, the razorbill genome was used as an outgroup combined with an approximated divergence time between razorbill and puffin.

For the first approach, pairwise genetic distances between individuals were generated in ANGSD (*-doIBS 2 -makeMatrix 1*) using the "IBS/Outgroup" dataset. The mean genetic distance between the razorbill and all other puffin individuals was then converted into a generational mutation rate using the formula *2 x MeanGeneticDistance /*

DivergenceTimePuffinRazorbill x PuffinGenerationTime (*131*). The divergence time between the razorbill and the puffin was set at 34 Mya (*90*), while the generation time of the puffin has been estimated to be 14.2 years (*81*).

For the second approach, a polymorphic-site-ratio was calculated for each individual in the "NonVariant/Outgroup" dataset (except the razorbill) by dividing the number of individual polymorphic sites by the number of total sites (*78*). Alleles at heterozygous positions within each puffin individual and the razorbill were randomly sampled and polymorphic sites in the puffins needed to be non-polymorphic in the razorbill. The mean polymorphic-site-ratio across all individuals was then converted to a generational mutation rate using the formula *MeanPolymorphicSiteRatio / DivergenceTimePuffinRazorbill x PuffinGenerationTime (78)* with the divergence time and generation time from above. The final generational mutation rate of 1.7125×10^{-8} used for all subsequent analyses was the mean between the mutation rates estimated via the "genetic distance" and "polymorphism" approaches.

2.2.7 Recombination map estimation

A recombination map used for several downstream applications was approximated using previously published research on the flycatcher genome (*132*). An assumption was made that the chromosomes of the puffin have a uniform recombination rate based on chromosome sizes and the recombination rate provided in Table 5 in Kawakami et al. (*132*) (Table S7).

2.2.8 Demographic history reconstruction

The demographic history, i.e. temporal fluctuations in effective population size (N_e), of the Spitsbergen and Røst populations were reconstructed using a variety of programs with different temporal resolution that each utilize different underlying genomic signals. These included the pairwise sequentially Markovian coalescent (PSMC; (*82*)), StairwayPlot2 (*84*), the multiple sequentially Markovian coalescent (MSMC2; (*85*)), and GONE (*88*). Finally, major past warming and cooling periods in the North Atlantic (*31*, *32*, *133–135*) were added to the resulting plots.

For PSCM, a consensus fastq file was generated with samtools v1.9 (*123*) and BCFtools v1.9 (*126*) for each of the 12 puffins, filtering out sites with a depth of less than 10 or more than double the mean coverage of that puffin, and with a root-mean-square mapping quality of reads covering the site under 25 (*82*, *83*). The fastq file was converted to a PSMC input file with the *fq2psmcfa* command removing sites with an inferred consensus quality below 20 (*82*, *83*). Using the settings "*-N30 -t5 -r5 -p 4+30*2+4+6+10*" as done for 38 other bird species by Nadachowska-Brzyska et al. (*83*), a pilot run was conducted to ensure that at least 10 recombination events occurred in each atomic interval after 20 iterations (*136*, *137*). Upon confirmation, parameters for the final PSMC runs were set to "*-N30 -t5 -r5 -p 4+30*2+4+6+10*" for all individuals, which were also used for 100 bootstrap runs for each individual using the *-b* flag (*82*). Fluctuations in N_e were plotted using the mutation rate of 1.7125×10^{-8} and a generation time of 14.2 years (*81*).

Prior to running StairwayPlot2, a folded population-based site frequency spectrum (SFS) was generated by splitting the "NonVariant" SNP dataset into populations and supplying it to the *easySFS.py* script (https://github.com/isaacovercast/easySFS, v0.0.1, accessed December 2022) without "down projection" for any population. StairwayPlot2 was run with 67% of sites for training, four different breakpoints (3,5,8,10), 200 bootstrapping

rounds, the mutation rate of 1.7125×10^{-8} and a generation time of 14.2 years (*81*), as suggested by the developers (*84*).

The MSMC2 analysis was performed with phased SNP data. In order to phase the 12 individual genomes, phase sets were identified in each individual puffin genome by WhatsHap v0.18 (--tag=PS; (*86*)) using the "NonLD-pruned/NoRelatedInd" dataset. The phase sets were then used as input for Shapeit4 v4.1 (*87*) to statistically phase (--use-PS 0.0001) all puffin genomes. After combining the phased SNP data with the "NonVariant" dataset using BCFtools v1.9, mappability files for each chromosome were produced with GenMap v1.3.0 (*138*) as in Deng et al. (*139*). Individual per-chromosome calling mask files and individual MSMC2-specific vcf files were created with the *script vcfAllSiteParser.py*, which is part of the MSMC2 tools. These were used as input for the *generate_multihetsep.py* script (MSMC2 tools) to combine all samples (11 - one related individual removed) into specific MSMC2 per-chromosome input files. Coalescence rates were estimated for all unique combinations of 2 (out of 5) different individuals from Spitsbergen versus 2 (out of 6) different individuals from Røst. For each combination, this included an estimation of coalescence rate within the 2 individuals (4 haplotypes) of the same population as well as across all 4 individuals (8 haplotypes) combined using the settings "*-t 24 -s -p 1*2+22*1+1*2+1*6*". The first and last time intervals were grouped into larger segments to prevent overfitting of the data (*85*). The estimated coalescence rates and scaled times were converted to N_e and years using the mutation rate of 1.7125×10^{-8} and a generation time of 14.2 years (*81*). Relative cross-coalescence rates, calculated as 2 * (across population coalescence rate)/(sum of within population coalescence rates), were used to evaluate when the Spitsbergen and Røst populations began to separate.

As opposed to the other three tools, the software package GONE is able to estimate fluctuations in N_e in the very recent past by assessing linkage disequilibrium decay patterns. For this analysis we used the "NonLD-pruned" dataset. Prior to running GONE, all chromosomes were randomly downsampled to a maximum of 500k SNPs per chromosome for computational purposes. GONE was then run per population with default settings (*88*; e.g. 40 bootstrapping iterations that randomly sample 50,000 SNPs) and with a mean genome-wide recombination rate of 1.63 cM/Mb (Table S7). The results of 100 replicate runs with randomly subsampled SNPs were subsequently scaled by years with a generation time of 14.2 years (*81*) and provided a median estimate of N_e as well as 95% confidence intervals. Each population trajectory was offset by the year the sampling took place (2016-2018, Supplementary Data S1).

2.2.9 Detection of admixture

Corroborating the detection of admixture on Bjørnøya by previous research (*19*), *f*3-statistics, D statistics and the f4-ratio were calculated. *f*3-statistics were determined with *threepop* in Treemix v1.13 (*89*) for each unique combination ((A,B),C)) of the three puffin populations via resampling the data in blocks of 500 SNPs (*-k 500*). Significantly negative values of the *f*3 statistic (Z-score < -3) are evidence of admixture between population A and B in population C.

The D statistic (also called Patterson's D or ABBABABA statistic) and the f4-ratio, which is a proxy for the proportion of admixed genome, were calculated with the program Dsuite (*90*) using the "NonLD-pruned/NoRelatedInd/Outgroup" dataset. Running the subcommands *Dtrios* and *DtriosCombine*, different combinations of Spitsbergen, Bjørnøya,

and Røst in the format (((P1,P2),P3),Outgroup) were tested and the D statistic and f4-ratio were calculated for the most significant (the highest proportion ABBA over BABA sites) combination. The potentially significant pattern of excess of ABBA sites over BABA sites leads to positive D statistics and indicates gene flow or admixture between P2 and P3 (*90*).

2.2.10 Introgressed local ancestry tracts and haplotype-based admixture timing

Two different approaches were applied to estimate the time or onset of admixture between the two subspecies (Spitsbergen/*naumanni* and Røst/*arctica*) on Bjørnøya. The first approach used RFmix v2 (*91*), which applies discriminative modeling using random forests trained on reference panels and a window and conditional random field to infer local ancestry tracts (*91*). The approach then utilized the theoretical relationship between length of introgressed tracts, time of admixture, recombination rate and admixture fractions (*92*). The second approach combined Chromopainter2 (*93*), and fastGlobetrotter (*94*). Chromopainter2 compares the genotypes of the admixed individuals to genotypes from ancestry reference populations to infer local ancestry. FastGlobetrotter exploits the fact that the probability of inheriting two DNA segments from the same ancestral source along the genome of an admixed individual decays exponentially with a rate proportional to the date of admixture and genetic distance between the segments (*94*). As both approaches require phased genomes, the phased SNP dataset (see the MSMC2 analysis in *Demographic history reconstruction)* was used.

For the first approach, genomic coordinates of SNPs needed to be converted to centiMorgans with chromosome-specific recombination rates (Table S7). RFMix v2 was subsequently run on all Bjørnøya individuals per chromosome with 50 EM iterations and the *"--reanalyze-reference*" flag to account for the fact that reference haplotypes (Spitsbergen and Røst) may not be of "pure" ancestry. Following previous research (*95*, *140*), length of introgressed Spitsbergen tracts (L) and global diploid Spitsbergen ancestry estimates (f) in Bjørnøya individuals generated by RFMix, together with the chromosome-specific recombination rate (r in Morgans per bp) were subsequently supplied to the equation $L=[(1-f)*r*(T_{ADMIX}-1)]^{-1}$ (*92*) to estimate the individual-based and chromosome-specific age of admixture between Spitsbergen and Røst on Bjørnøya. The distribution of T_{ADMIX} across all 24 chromosomes and six individuals was bootstrapped 10,000 times to identify the average maximum density value of each bootstrap replicate distribution and the 95% confidence intervals (0.025 and 0.975 quantiles of maximum density values; (*95*, *140*)).

For the second approach, the published guidelines (https://github.com/hellenthal-group-UCL/fastGLOBETROTTER/blob/master/FastGLOBETROTTER_Tutorial.pdf, accessed December 2022) were followed and the genomes of the Bjørnøya individuals were "painted" (i.e. local ancestry probabilities for each SNP per haplotype were determined) with Chromopainter2 with chromosome-specific recombination rates (Table S7) and by estimating switch and emission rates with chromosomes 2, 7, 12, 17, 22 and 24. A final Chromopainter2 run was conducted with parameters "0 0 -s 10 -n 16885.113 -M 0.017381". Subsequently, "painted" genomes were supplied to fastGlobetrotter, which was run with default settings and chromosome-specific recombination rates (Table S7). FastGlobetrotter suggested a 1-pulse over a 2-pulse admixture scenario due to the better model fit. A total of 100 bootstrap replicates of the proposed admixture scenario were generated and analyzed to determine 95% confidence intervals for the estimated admixture timings.

3. Historical Genomic Analyses

3.1 Sampling, Sequencing and Data Processing

Dried skin samples from a total of 22 puffins from three breeding areas (Spitsbergen, Bjørnøya, Røst Archipelago) were made available by the Natural History Museum Stockholm and the American Museum of Natural History. These samples date from 1860-1910 and consisted of small slices of toe pads (Supplementary Data S1). The areas were chosen to represent the historical counterparts to the analyzed modern individuals of the subspecies *F. a. naumanni* on Spitsbergen, *F. a. arctica* at Røst and of the hybrid population on Bjørnøya.

All individuals were adults and were originally collected between the breeding months of June-August. The specimens from the Vesterålen/Røst archipelago were collected by Alfred Edmund Brehm during his expedition to Norway in 1860. The individuals from Bjørnøya were collected during the "Zeppelin-Studienfahrt" in the summer of 1910. On the 12th of August 1910, a team led by the ornithologist Otto Graf von Zedlitz arrived on Bjørnøya and shot ca. 20 puffins at their breeding spot (*141*). Due to their substantially smaller size, two of the 10 analyzed individuals from Spitsbergen and one of the individuals from Røst were assumed to be members of the smaller-bodied subspecies *F. a. arctica* and *F. a. grabae*, respectively, and were therefore called "mutants" or "migrants" by the original authors ((*68*); Figure S10).

DNA was extracted following slightly customized protocols from previous genetic studies using historical avian specimens (e.g. (*142*, *143*)). Samples were first washed and rehydrated by consecutive rinses in 100% EtOH, 10mM TrisHCl, and nuclease-free water. Rehydrated skins were digested at 52°C overnight in 1.5 ml of lysis buffer containing 0.5M EDTA, 10% SDS, 1M DTT and 75µl of proteinase K. Lysates were centrifuged for 5 min at 13,000 rpm and the supernatant transferred to 50 ml falcon tubes. After adding 2ml of PB buffer (Qiagen), DNA was filtered and purified with MinElute columns (Qiagen) using the QIAvac 24 Plus vacuum manifold system (Qiagen). DNA was eluted in 2 x 35 µl of preheated (55°C) EB Buffer (Qiagen) after a 10 min incubation at 37°C.

After measuring isolated DNA concentrations with a Qubit fluorometer (Invitrogen®), extracts were diluted with EB buffer to provide 5-10 ng of DNA in 5 ul of extract as input for genomic library preparations. The single-stranded Santa Cruz Reaction (SCR) library protocol with tier 4 adapter dilutions (*69*) was used to build single-indexed sequencing libraries. Library quality and concentration was inspected with a High Sensitivity NGS Fragment Analysis Kit on the Fragment AnalyzerTM (Advanced Analytical). Libraries were sequenced at the Norwegian Sequencing Centre on a Novaseq S4 in 150 bp paired-end mode.

Negative controls were included for all extraction and library preparation sessions. All laboratory protocols up to indexing of sequencing libraries were carried out in the dedicated ancient DNA clean laboratory at the University of Oslo following standard protocols to minimize contamination (*144*, *145*).

Sequencing reads from the 22 historical specimens as well as the 77 modern puffins from previous genomic puffin research (*19*, *27*) were processed in PALEOMIX v1.2.14 (*65*). Specifically, after removing adapters from forward and reverse reads with AdapterRemoval v2.3.1 (*124*) (*--mm3 --minlength25 --collapse yes --trimns yes --trimqualities yes*), reads were mapped to the new Atlantic puffin reference genome using BWA *mem* v0.7.17 (*96*).

Reads that aligned with a quality score (MapQ) of ≥ 25 were kept for duplicate removal with PicardTools v2.18.27 (*125*) and indel realignment using GATKs *IndelRealigner (66)*.

The sex that was assigned to each historical individual was checked by determining the proportion of sequencing reads mapping to the Z and W chromosome. Males (ZZ) were characterized by having a Z-specific depth of coverage of ca. 2 x avg. autosomal depth of coverage and a W-specific depth of coverage of ca. 0 x avg. autosomal depth of coverage. Females (ZW) had a Z- and W-specific depth of coverage of ca. 1 x avg. autosomal depth of coverage.

3.2 Population genomic analyses

3.2.1 Genotype Likelihoods

All population genomic analyses were based on genotype likelihoods as implemented in ANGSD v.0.935 (*67*). Prior to calculating genotype likelihoods, the quality of the mapped sequencing data (historical + modern) after removing transitions and one related individual (see *3.2.2 Linkage disequilibrium pruning & Relatedness*) was assessed in an ANGSD pre-run using *"-uniqueOnly 1 -remove_bads 1 -minMapQ 25 -maxDepth 1100 -checkBamHeaders 1 -C 50 -baq 2 -rmTrans 1 -doQsDist 1 -doDepth 1 -doCounts 1 -dumpCounts 2 -GL 1 -doMajorMinor 1"*. The statistics "Global Depth vs. No. of sites", "Genotyping Rate Cutoff vs. No. of remaining sites", and "Depth of coverage per individual versus the proportion of sites" was calculated and visualized in R v3.6 (*146*) to determine the appropriate cutoffs for depth and genotyping rate (Figure S25).

Genotype likelihoods for SNPs covered in at least 95 of 98 (22+76) individuals were calculated and filtered in ANGSD with *"-uniqueOnly 1 -remove_bads 1 -minMapQ 30 -minQ 30 -C 50 -baq 2 -checkBamHeaders 1 -HWE_pval 1e-2 -sb_pval 1e-5 -hetbias_pval 1e-5 -skipTriallelic 1 -minInd 95 -snp_pval 1e-6 -minMaf 0.05 -setMaxDepth 875 -setMinDepth 379 -rmTrans 1 -doMajorMinor 1 -doMaf 1 -doCounts 1 -doGlf 2 -doHWE 1 -dosnpstat 1"* and stored in *beagle* format. Hardy-Weinberg-disequilibrium filters were applied to remove genotyping errors and loci under selection, which is required for analyses that assume the neutrality of loci.

The dataset was pruned to account for linkage disequilibrium. R^2 was calculated for pairs of sites within 50 kb windows along all chromosomes using ngsLD (*147*). Linked sites (r^2>0.2) were clustered into larger groups using *mcl (148)* in the software OrthoMCL v2.0.92 (*149*), and the most central site was selected as representative of each block for subsequent analyses. Additionally, all variants located on the Z-chromosome were excluded from the analyses.

3.2.2 Principal Component Analysis (PCA) and ancestry proportions

The genomic population structure was investigated by running PCAngsd v0.982 (*72*) with default settings and plotting the eigenvectors and eigenvalues of the two principal components explaining most observed genetic variation. Individual ancestry proportions were estimated using a maximum likelihood (ML) approach implemented in ngsAdmix v32 (*73*) by setting the number of ancestral populations, K, from 1 to 10 and conducting 50 replicate runs for each K. The optimal value of K was chosen based on the method of Evanno (*130*) using Clumpak (*129*) with default settings. The fit of K to the dataset was evaluated with evalAdmix v0.95 (*74*) by using the allele frequency estimates and q-matrix from NGSadmix with the highest log-likelihood.

3.2.3 Tajima's D and Nucleotide Diversity

A set of neutrality tests and population statistics were calculated using colony- and genomic cluster based one-dimensional (1D) folded Site-Frequency-Spectra (SFS). A SNP panel without transitions, covered in at least 95 individuals and passing several quality filters without removing rare alleles was selected in ANGSD with *"-uniqueOnly 1 -remove_bads 1 -minMapQ 30 -minQ 30 -dosnpstat 1 -C 50 -baq 2 -checkBamHeaders 1 -doHWE 1 -sb_pval 1e-5 -hetbias_pval 1e-5 -skipTriallelic 1 -minInd 95 -setmaxDepth 875 -setminDepth 379 -rmTrans 1 -doMajorMinor 1 -doMaf 1 -doCounts 1"*. The dataset was further pruned by filtering out potentially lumped paralogs (*150*). As described in detail in Kersten et al. (*19*), site allele frequency (SAF) likelihoods (*saf.* files) were estimated for each population and genomic cluster in ANGSD and folded 1D-SFS generated by *winsfs* v0.6.0 (*77*) were used to compute Tajima's D and nucleotide diversity (**π**) per chromosome. Significance of differences in nucleotide diversity and Tajima's D between historical populations and their modern counterparts was determined with a one-sided two-sample Wilcoxon rank sum test (*151*).

3.2.4 Heterozygosity, Runs-of-Homozygosity, and Inbreeding

Individual genome-wide heterozygosity was calculated in ANGSD using the puffin reference genome as ancestral reference. The individual, folded, 1D-SFS (see *Tajima's D and Nucleotide Diversity*) was estimated with ANGSD (-doSaf) and *winsfs*, and heterozygosity was calculated by dividing the number of polymorphic sites by the number of total sites present in the SFS. Statistical significance of differences in heterozygosity between historical populations and their modern counterparts was assessed with a one-sided two-sample Wilcoxon rank sum test (*151*).

The proportion of runs of homozygosity (RoH) within each individual puffin genome was computed as in Kersten et al. (*19*). In short, local estimates of heterozygosity were calculated in sliding windows using window-based SFS. The 10% quantile of the average local heterozygosity across all modern samples was defined as the cutoff for a "low heterozygosity region" and was set to 0.482×10^{-3} (Figure S27). RoH were declared as all regions with at least two subsequent windows of low heterozygosity. The minimum RoH length was 150 kbp and increased in steps of 50 kbp. An individual inbreeding coefficient based on the RoH, F_{RoH} , was calculated as in Kersten et al. (*19*). Statistical significance of differences in average RoH length and F_{RoH} between historical populations and their modern counterparts was assessed with a one-sided two-sample Wilcoxon rank sum test (*151*).

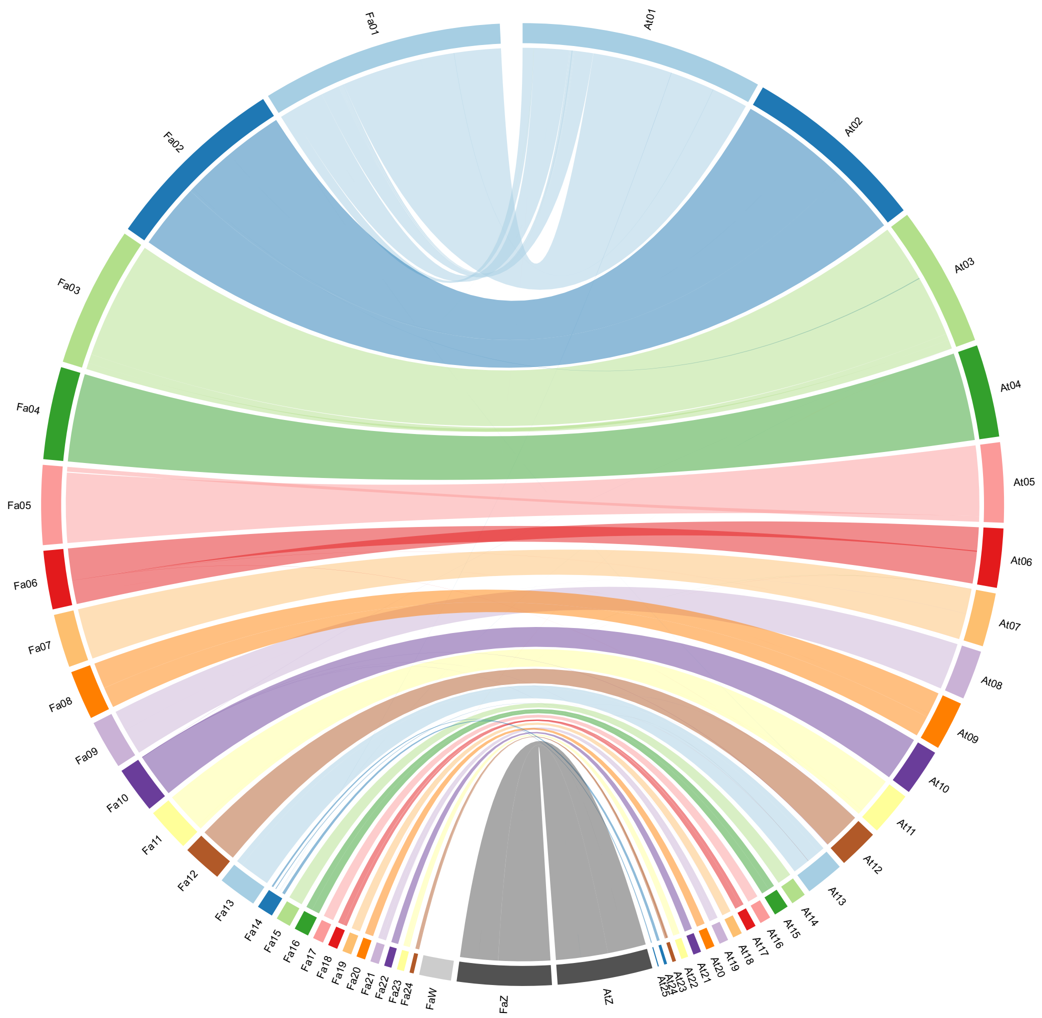

Fig. S1: Chromosomal synteny between the Atlantic puffin (*Fratercula arctica*; left side, denoted with the prefix Fa) and the razorbill (*Alca torda*; right side, denoted with the prefix At). The W-chromosome is missing for the razorbill as the sequenced individual used for the assembly of its reference genome was a male.

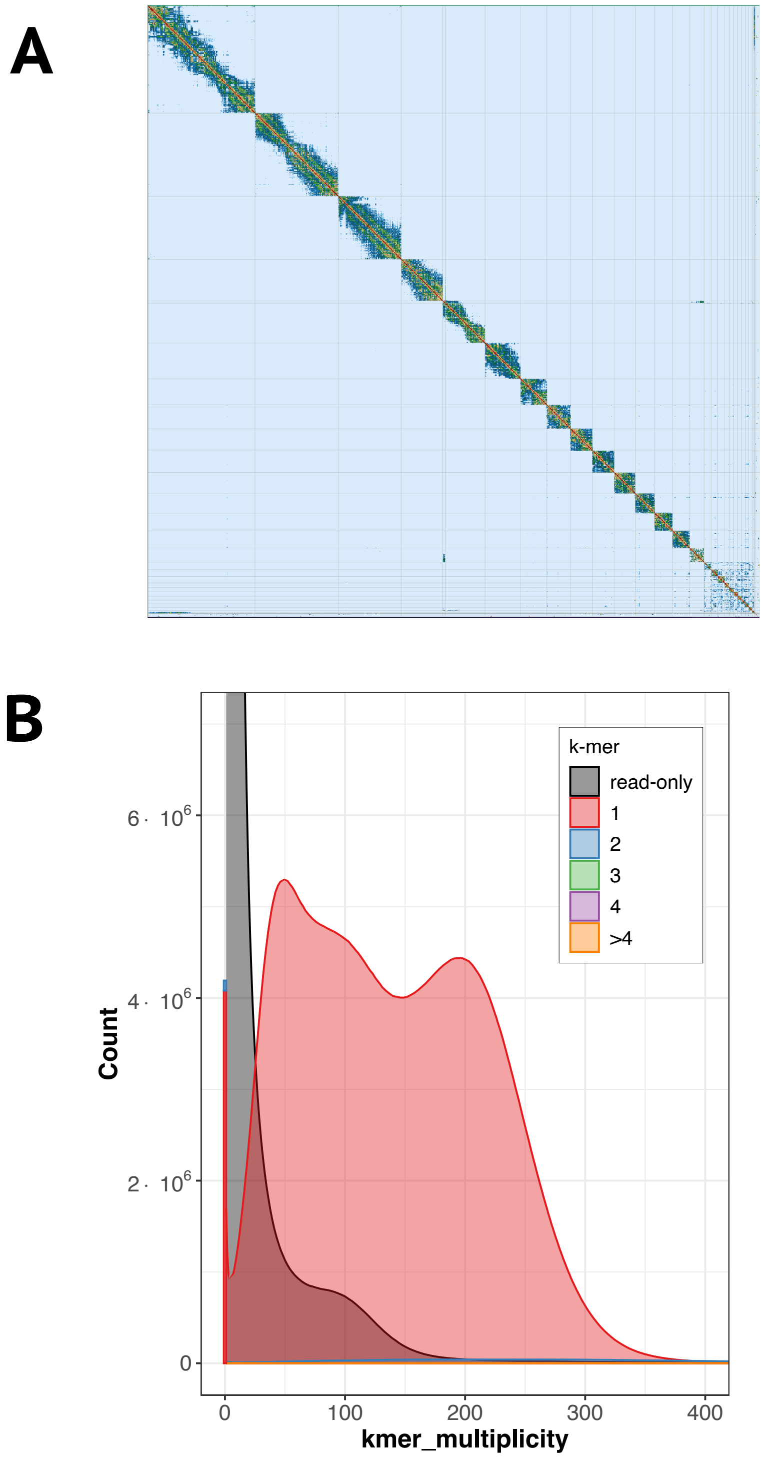

Fig. S2: Heatmap of Hi-C data and copy number spectrum (spectra-cn) plot for the newly assembled Atlantic puffin reference genome using PacBio, Hi-C and 10X Genomics data. A) Hi-C interactions among all chromosomes and scaffolds of the reference genome, as plotted by Pretext. B) Histogram of k-mer multiplicity collected from 10X Genomics reads, as plotted with Merqury. The first peak represents 1-copy (heterozygous) k-mers in the genome, and the second peak represents 2-copy k-mers originating from homozygous sequence or haplotype-specific duplications. Depth of sequencing coverage determines where these peaks appear. The assembly k-mers absent from the read set (likely to be base errors in the assembly) are plotted as a bar at zero multiplicity, colored by the copy numbers found in the assembly.

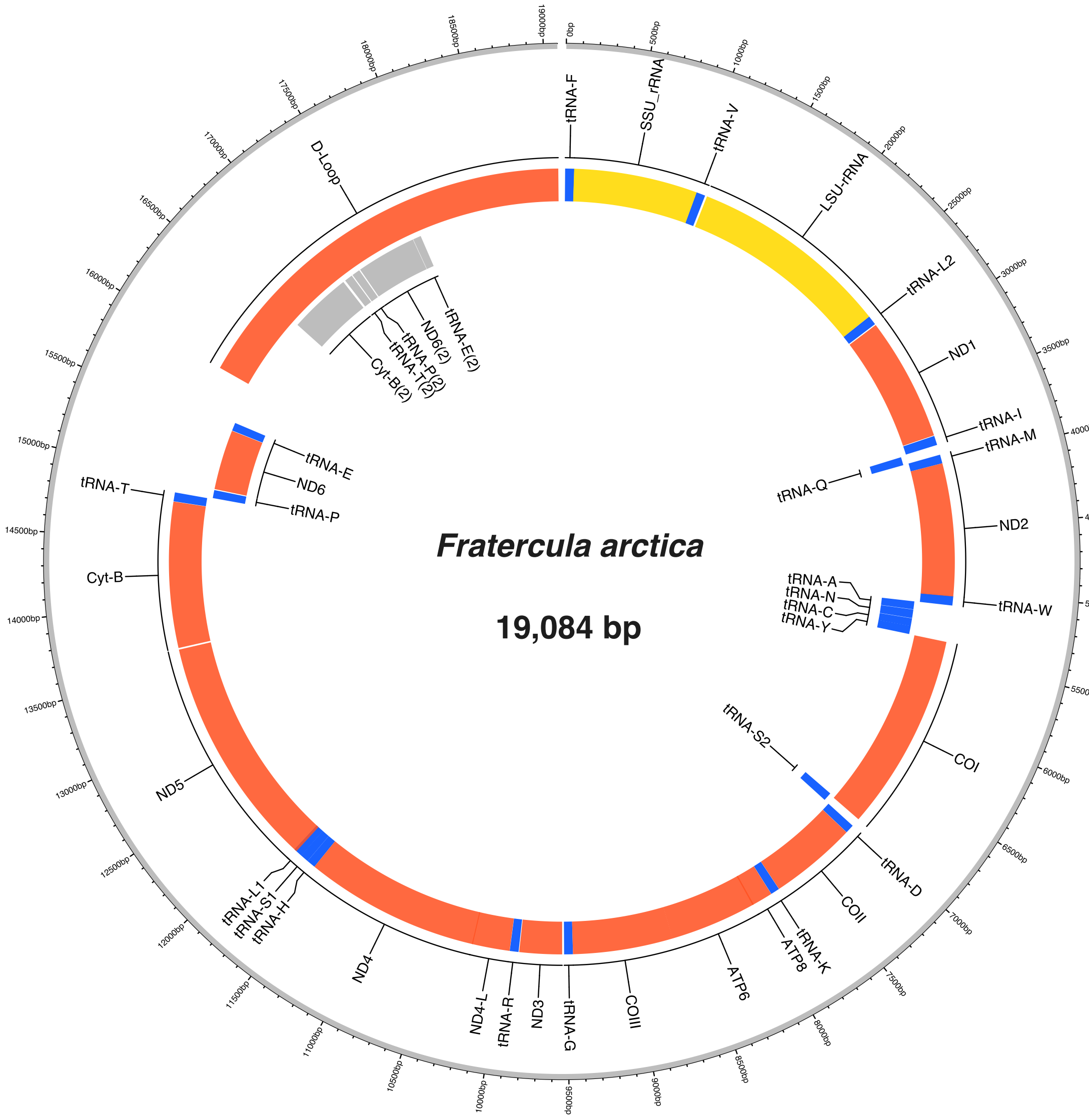

Fig. S3: Circular representation of the new mitogenome of the Atlantic puffin. Protein coding regions are highlighted in red, tRNAs in blue, and rRNAs in yellow. Outer ring depicts position in bp. Annotation was performed with the MITOS web server and the plot was produced with *shinyCircos*. The grey protein coding regions and tRNAs in the D-Loop represent a ca. 2kb long pseudo-duplicated region missing in the previously published assembly.

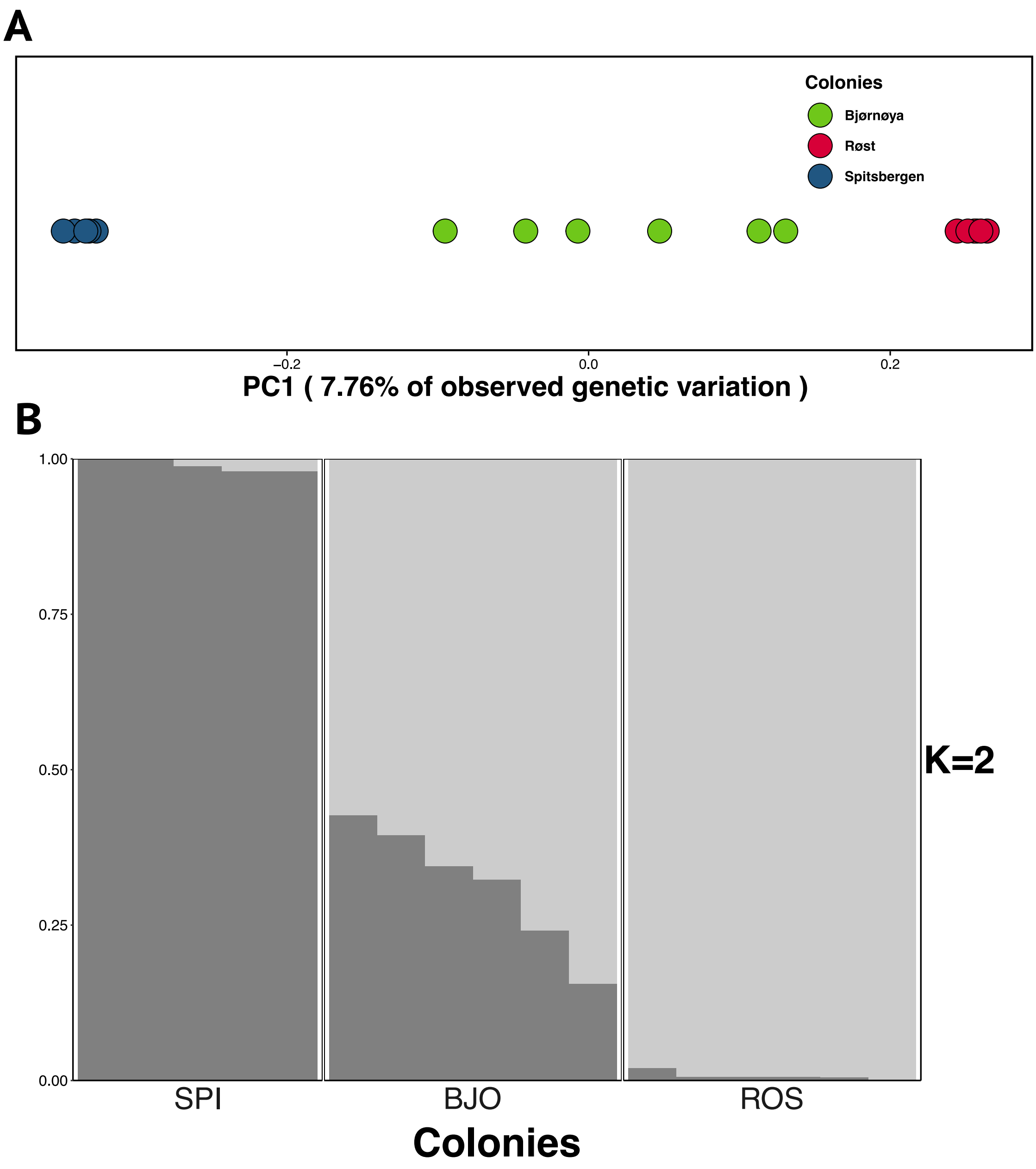

Fig. S4: Genomic population structure of 18 Atlantic puffin individuals across three breeding colonies. A) The Principal Component Analysis (PCA) was performed with smartPCA and projects each deep-sequenced (>20X coverage), modern individual onto PC axes 1 (PC axis 2 was not significant). The color coding of the colonies is consistently used throughout the manuscript. B) CLUMPAK-averaged admixture plots of the best K using the same SNPs as in A). Each column represents a sample and colonies are separated by solid white lines. The optimal value of K was determined by the method of Evanno et al. (*130*). SPI=Spitsbergen. BJO=Bjørnøya. ROS=Røst.

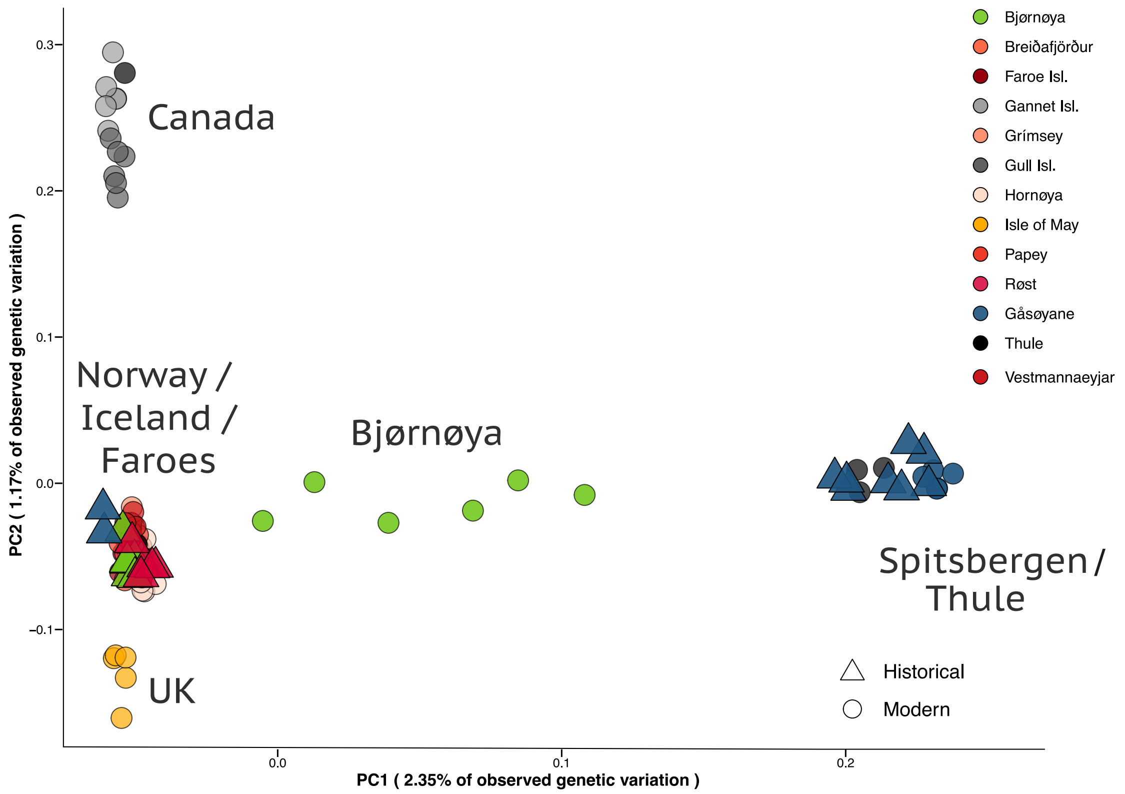

Fig. S5: Genomic population structure of modern and historical Atlantic puffins across 13 breeding colonies. The principal component analysis was performed with *PCAngsd* using medium-coverage (5-10X coverage) data for 98 individuals. Colors represent the locations samples were collected from (see legend), while labels in the figure represent the names of the identified genomic clusters. Historical samples are shown as triangles and modern samples as circles.

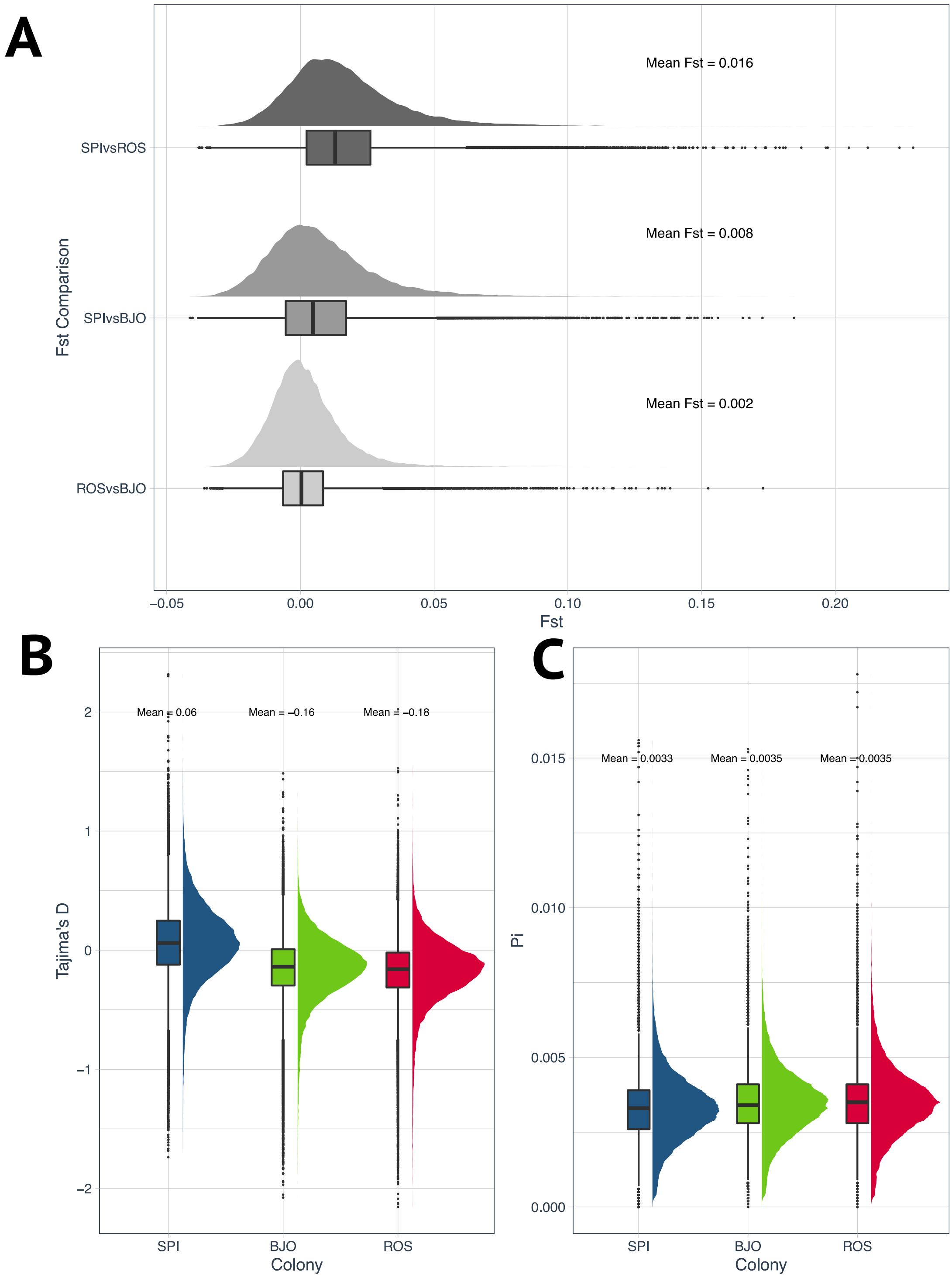

Fig. S6: Genomic differentiation (F_{ST}), nucleotide diversity (Pi), and Tajima's D across three Atlantic puffin colonies using 18 contemporary individuals. Parameters were calculated using high-coverage (>20X) data and assessed genome-wide in 50kb sliding windows (25kb slide). SPI=Spitsbergen. BJO=Bjørnøya. ROS=Røst.

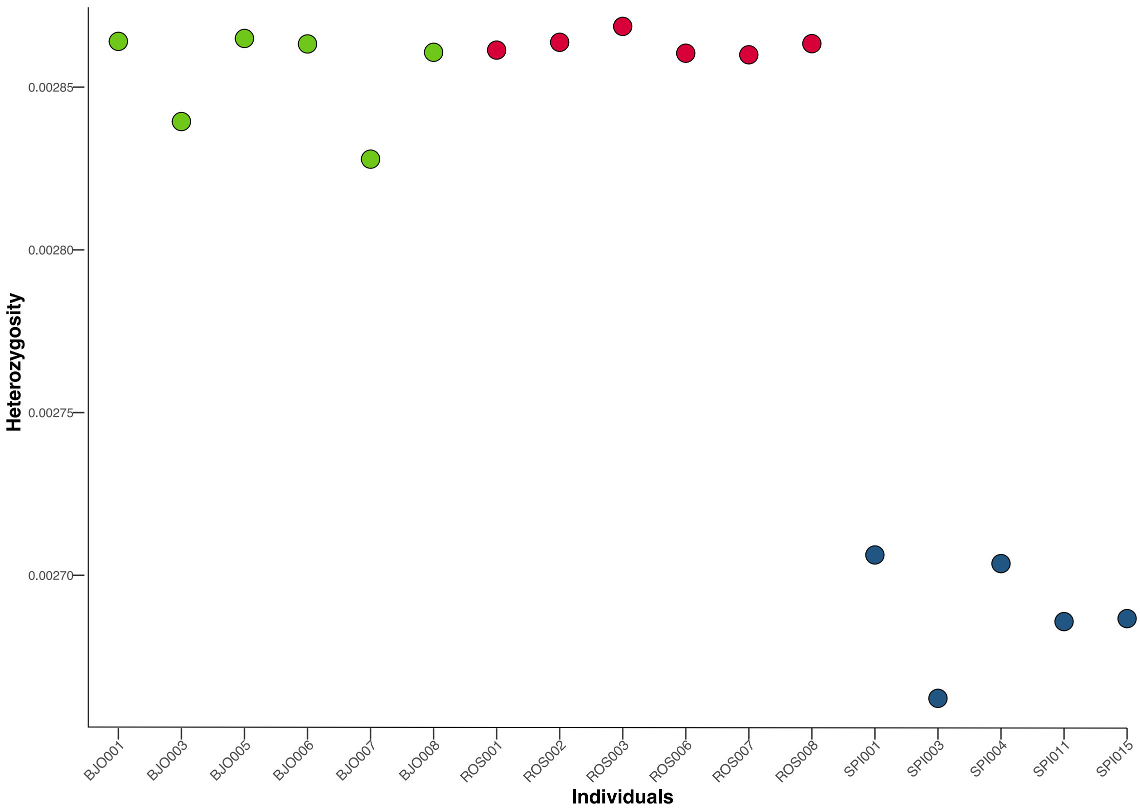

Fig. S7: Individual, genome-wide heterozygosity of 18 Atlantic puffins belonging to three different colonies. Heterozygosity was calculated using modern, high-coverage (>20X) sequencing data. SPI=Spitsbergen. BJO=Bjørnøya. ROS=Røst.

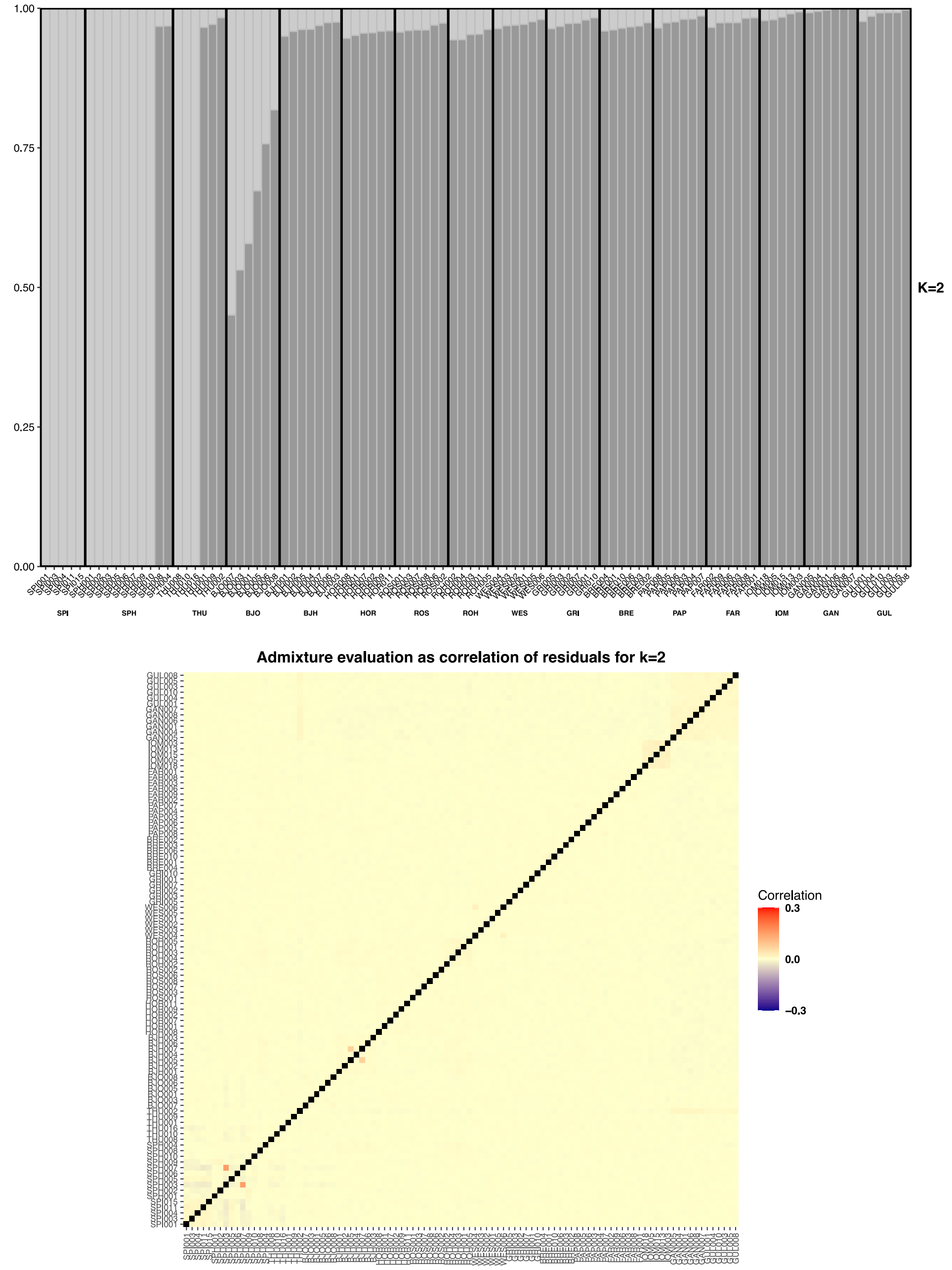

Fig. S8: Ancestry proportions among 22 historical and 76 modern Atlantic puffins for the optimal number of ancestral populations (K). The optimal K was determined by the method of Evanno et al. (*130*). The admixture plot (top) for K = 2 was generated with the program *ngsAdmix* using medium-coverage (5-10X) sequencing data and evaluated (bottom) with *EvalAdmix*. Each column (top) represents a sample and colonies are separated by solid white lines. The pairwise correlation of residuals matrix between individuals (bottom) highlights the goodness of fit of the data to the admixture model. When individuals do not fit the model, individuals with similar demographic histories (i.e. usually individuals from the same population) will be positively correlated. Individuals with different histories but that are modelled as sharing one or more ancestral populations as admixture sources will have a negative correlation. Positive correlation between a pair of individuals may also be due to relatedness. SPH=Spitsbergen (Historical), SPI=Spitsbergen (Modern), THU=Thule, BJO=Bjørnøya (Modern), BJH=Bjørnøya (Historical), HOR=Hornøya, ROS=Røst (Modern), ROH=Røst (Historical), WES=Vestmannaeyjar, PAP=Papey, BRE=Breiðafjörður, GRI=Grímsey, FAR=Faroe Isl., IOM=Isle of May, GAN=Gannet Isl., GUL=Gull Isl.

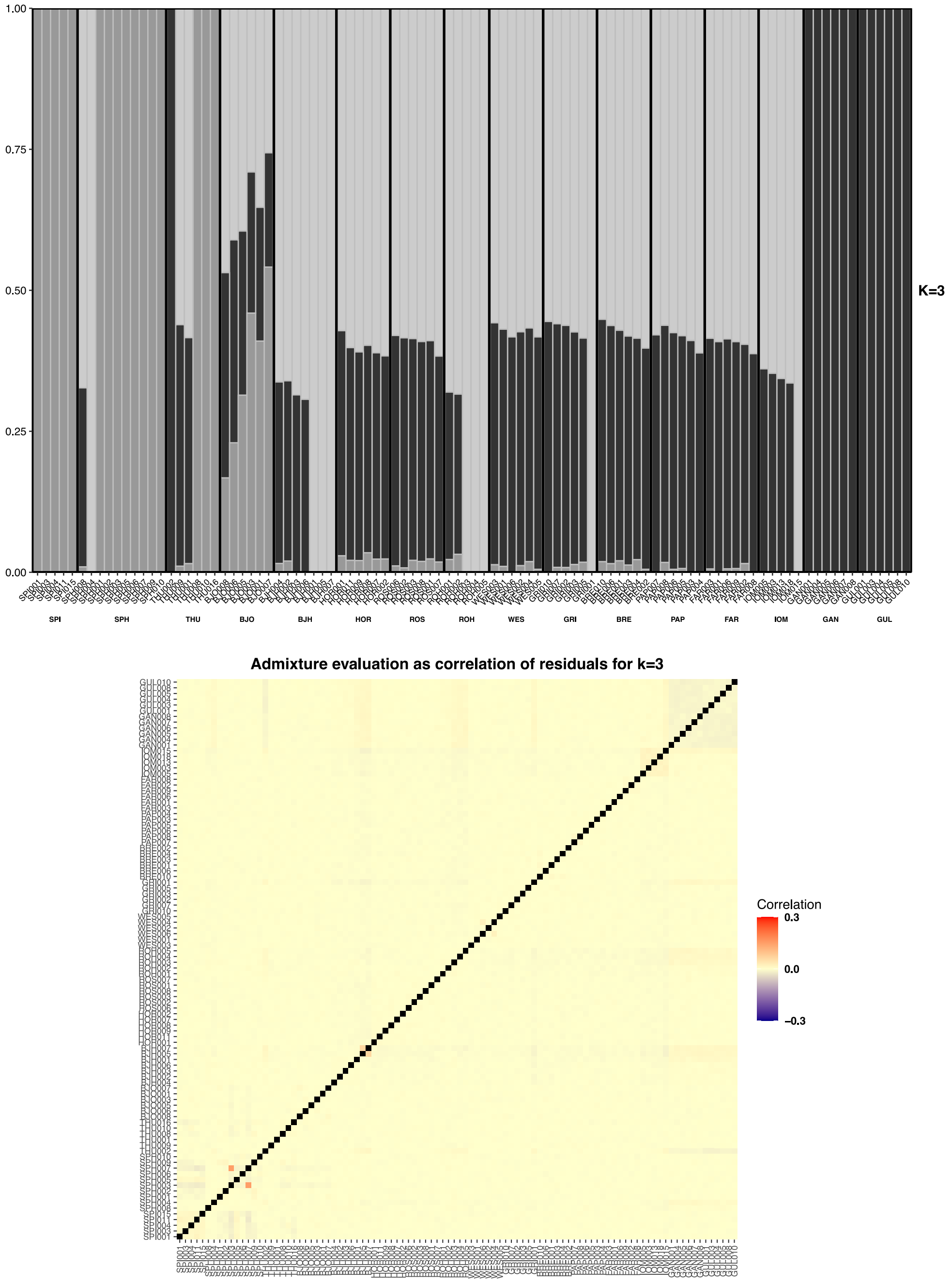

Fig. S9: Ancestry proportions among 22 historical and 76 modern Atlantic puffins for three ancestral populations. The admixture plot (top) for K = 3 was generated with the program *ngsAdmix* using medium-coverage (5-10X) sequencing data and evaluated (bottom) with *EvalAdmix*. Each column (top) represents a sample and colonies are separated by solid white lines. The pairwise correlation of residuals matrix between individuals (bottom) highlights the goodness of fit of the data to the admixture model. When individuals do not fit the model, individuals with similar demographic histories (i.e. usually individuals from the same population) will be positively correlated. Individuals with different histories but that are modelled as sharing one or more ancestral populations as admixture sources will have a negative correlation. Positive correlation between a pair of individuals may also be due to relatedness. SPH=Spitsbergen (Historical), SPI=Spitsbergen (Modern), THU=Thule, BJO=Bjørnøya (Modern), BJH=Bjørnøya (Historical), HOR=Hornøya, ROS=Røst (Modern), ROH=Røst (Historical), WES=Vestmannaeyjar, PAP=Papey, BRE=Breiðafjörður, GRI=Grímsey, FAR=Faroe Isl., IOM=Isle of May, GAN=Gannet Isl., GUL=Gull Isl.

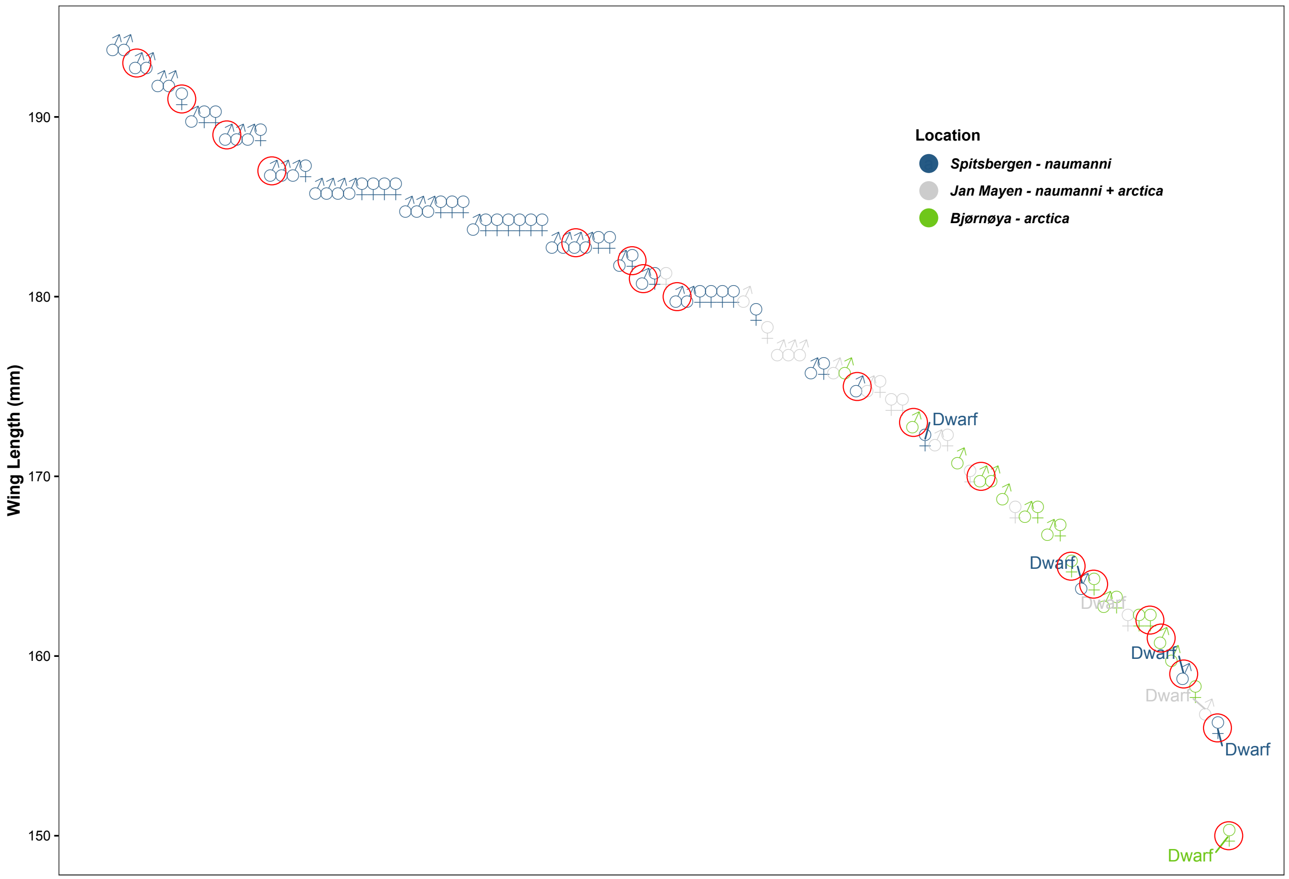

Fig. S10: Wing length (in mm) of historical Atlantic puffins sampled across Bjørnøya, Jan Mayen and Spitsbergen. Historical individuals were measured and sampled by Salomonsen (*68*) (p. 119**).** Individuals from Bjørnøya and Spitsbergen analyzed in this study are circled in red. Note the three individuals marked as "Dwarf" that were analyzed in this study.

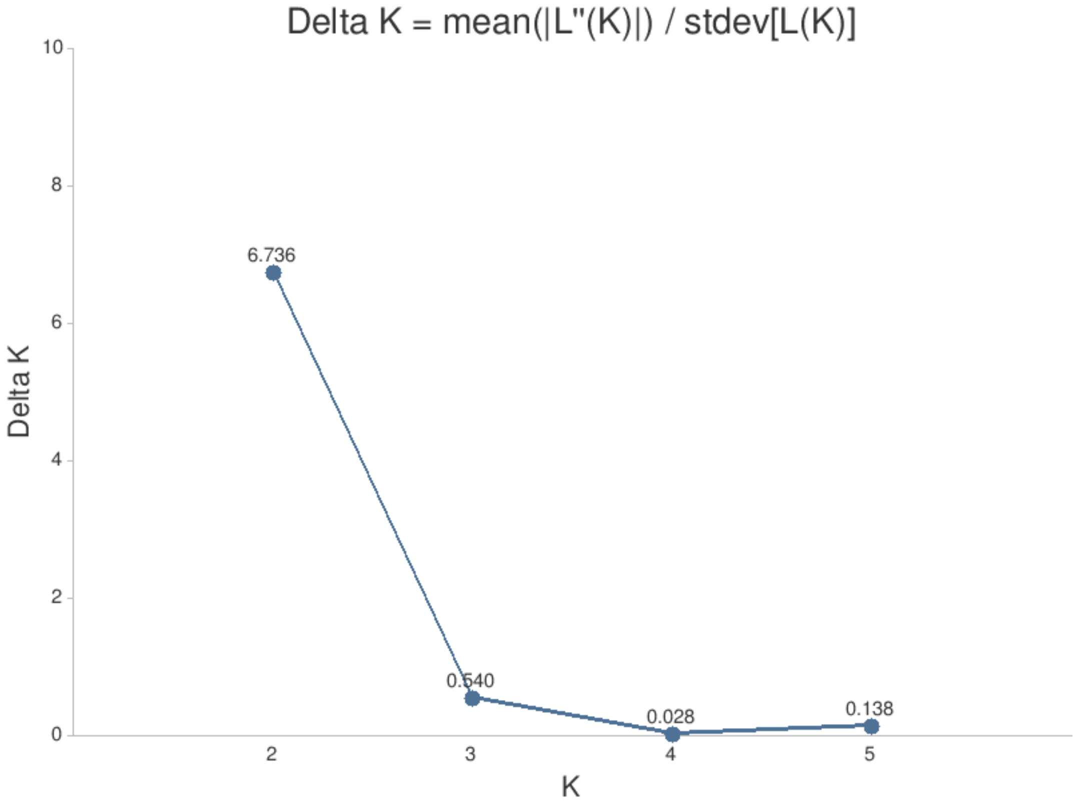

Fig. S11: Delta K as a function of the number of ancestral clusters (K) as calculated by the method of Evanno et al. (*129*) for K = 1-5. The optimal K for the subsequent admixture analysis is determined by the largest delta K(s).

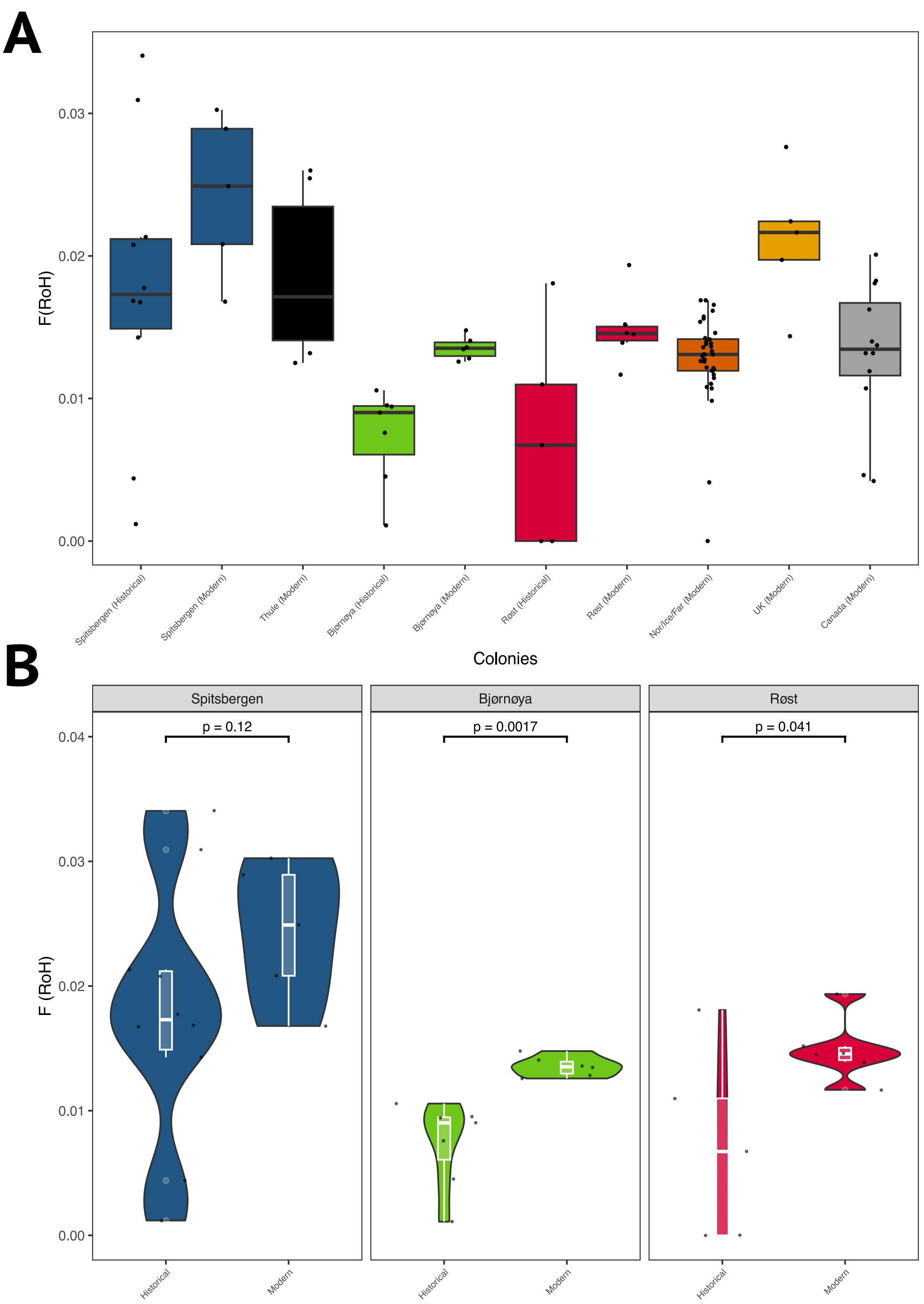

Fig. S12: Inbreeding of modern and historical Atlantic puffin colonies and genomic clusters. The dataset consisted of medium-coverage (5-10X) sequencing data from 98 individuals. Individual inbreeding coefficients, F_{RoH} , were compared between A) genomic clusters and B) modern populations and historical counterparts. F_{RoH} was defined as the fraction of the individual genomes falling into RoHs of a minimum length of 1 Mb (*19*). RoHs were declared as all regions with at least two subsequent 100 kbp windows harboring a heterozygosity below 0.482 x 10^{-3}. Black dots indicate individual sample estimates and black/white lines the median per population. Different populations in all plots are indicated using different colors consistent throughout the manuscript. Error bars in top and bottom plot show range of values within 1.5 times the interquartile range.

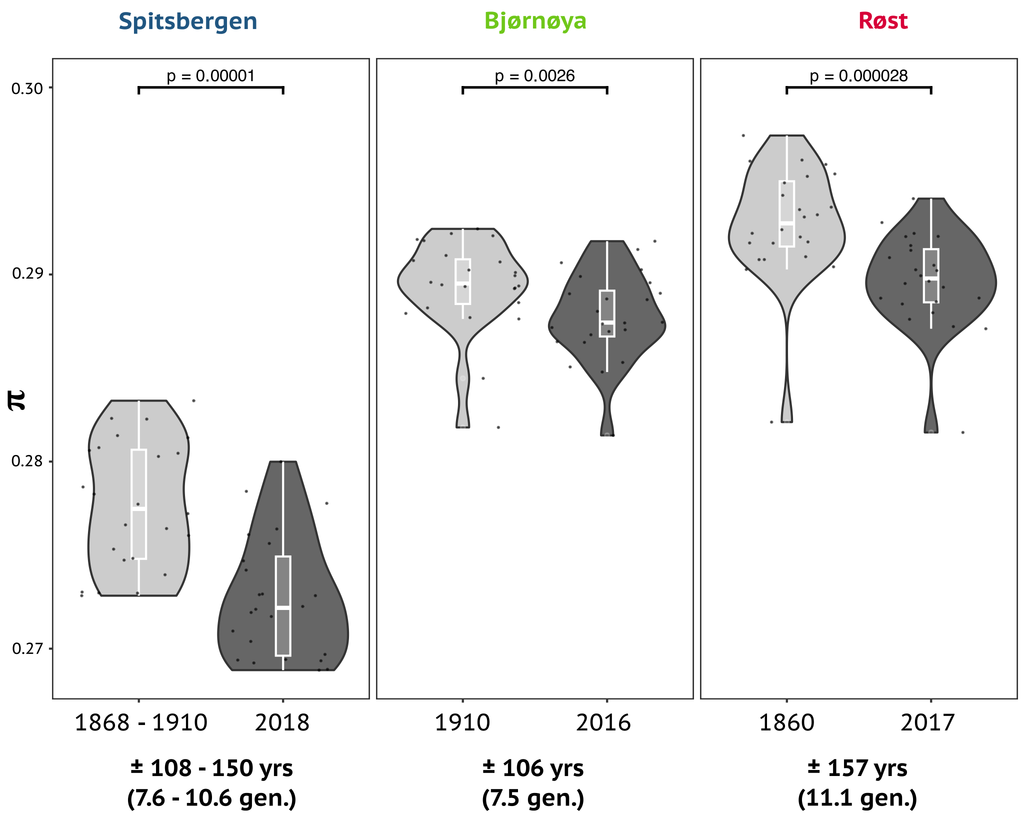

Fig. S13. Temporal changes in nucleotide diversity across three Atlantic puffin populations. Nucleotide diversity was estimated for the historical and modern populations of Spitsbergen, Røst and Bjørnøya representing the subspecies *F. a. naumanni, F. a. arctica,* and their hybrids, respectively. Nucleotide diversity for each population was calculated per chromosome (N=24, black dots) with the program ANGSD using a site-frequency-spectrum (SFS). The SFS included only sites that were thoroughly filtered and pruned for linkage disequilibrium, and used for the PCA and Admixture analyses in this manuscript. The x axes present sampling years and their differences in years and generations are given. Significance of differences is indicated by p-values.

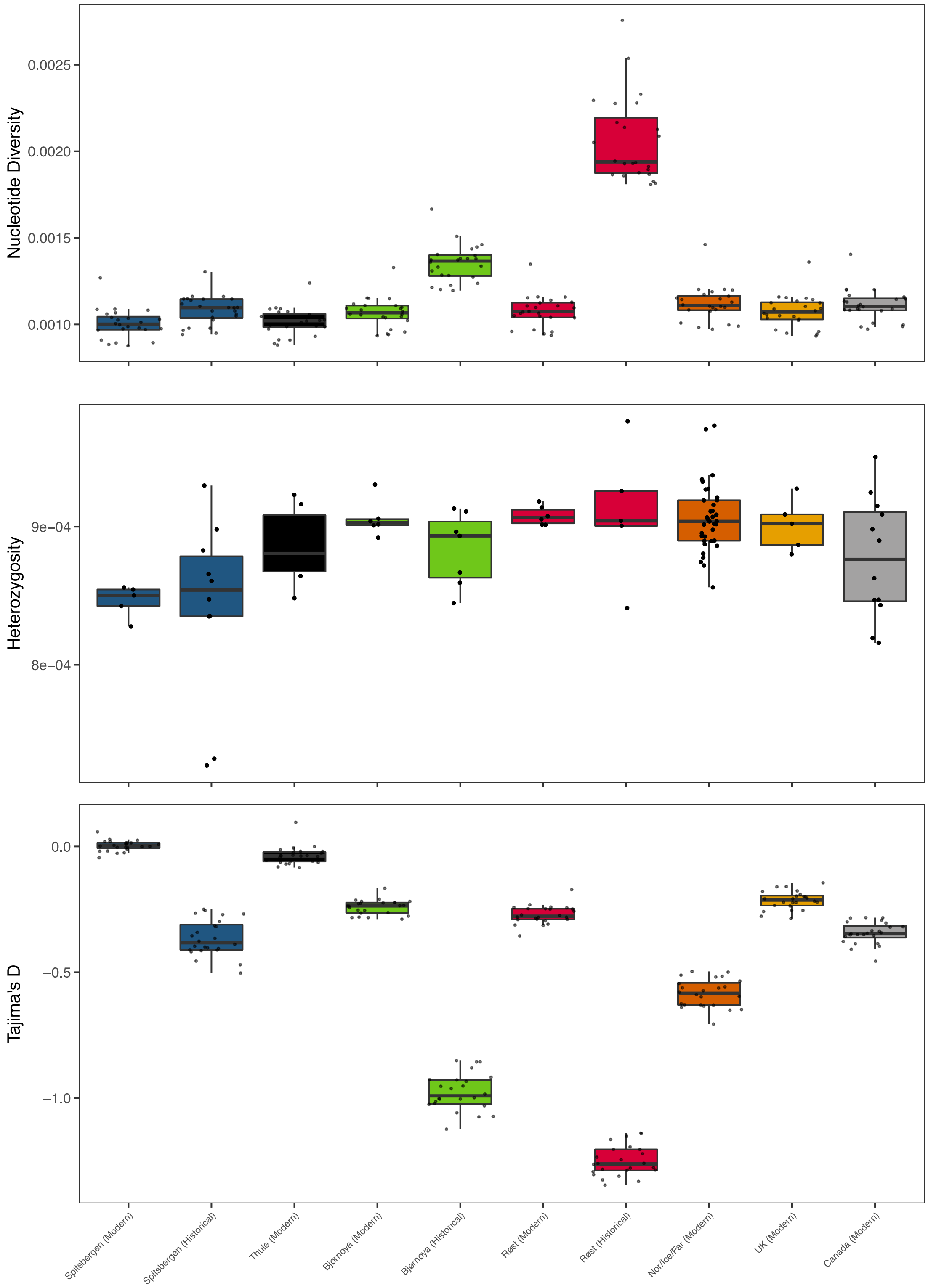

Fig. S14: Genome-wide nucleotide diversity, heterozygosity and Tajima's D of modern and historical Atlantic puffin colonies and genomic clusters. Estimates of heterozygosity were based on the per-sample one-dimensional Site Frequency Spectrum calculated in ANGSD using medium-coverage (5-10X) sequencing data from 98 individuals. Nucleotide diversity and Tajima's D were calculated with the per-population chromosome-based one-dimensional Site Frequency Spectra. Different populations in all plots are indicated using different colors consistent throughout the manuscript. Error bars show range of values within 1.5 times the interquartile range and black lines the median per population.

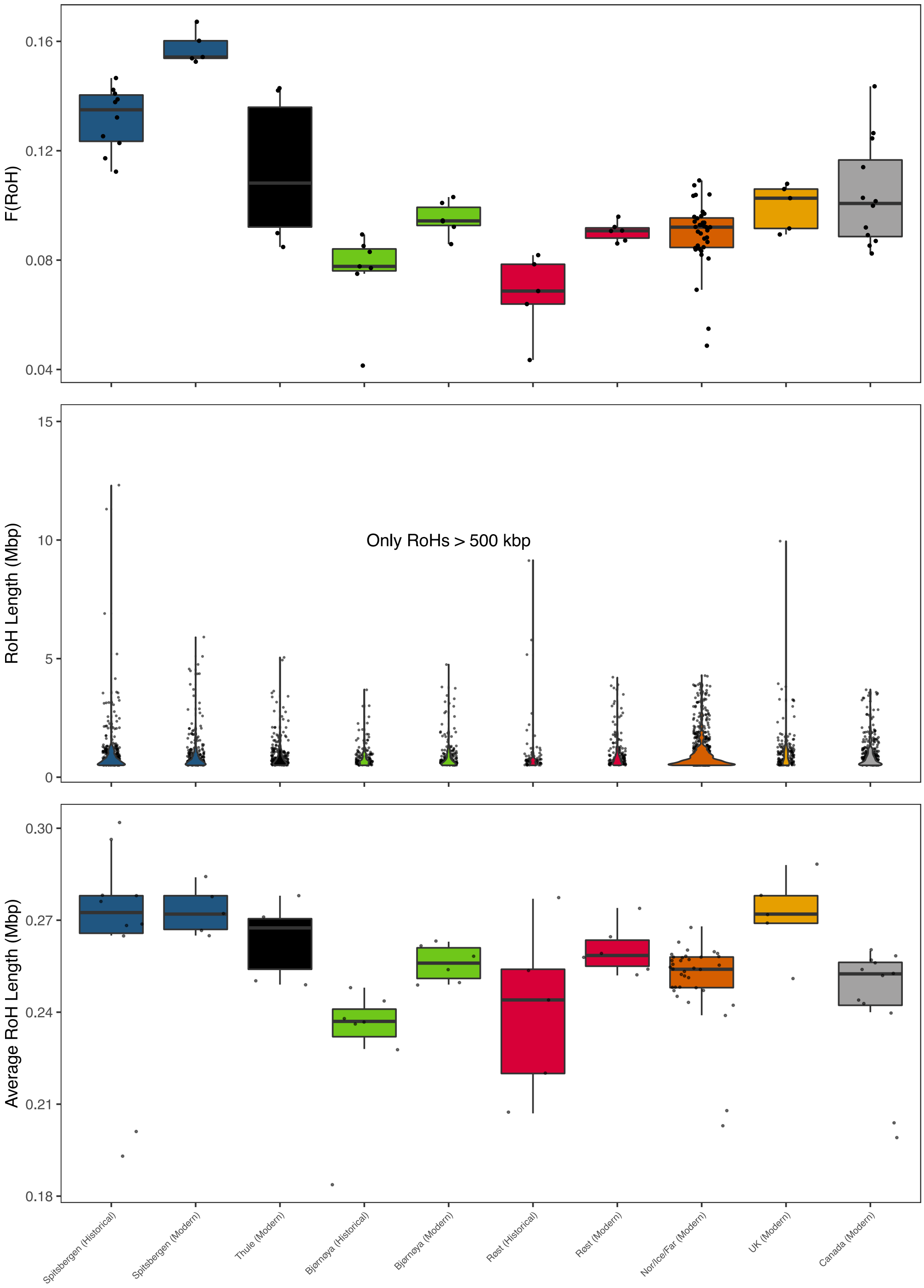

Fig. S15: Inbreeding and Runs of Homozygosity (RoH) of modern and historical Atlantic puffin colonies and genomic clusters. The dataset consisted of medium-coverage (5-10X) sequencing data from 98 individuals. Individual inbreeding coefficients, F_{RoH} , were defined as the fraction of the individual genomes falling into RoHs of a minimum length of 150 kbp (*19*). RoHs were declared as all regions with at least two subsequent 100 kbp windows harboring a heterozygosity below 0.482 x 10^{-3}. For F_{RoH} and the average RoH length, black dots indicate individual sample estimates and black lines the median per population. In the panel showing RoH Length, black dots represent individual RoHs across all samples of that population. Different populations in all plots are indicated using different colors consistent throughout the manuscript. Error bars in top and bottom plot show range of values within 1.5 times the interquartile range.

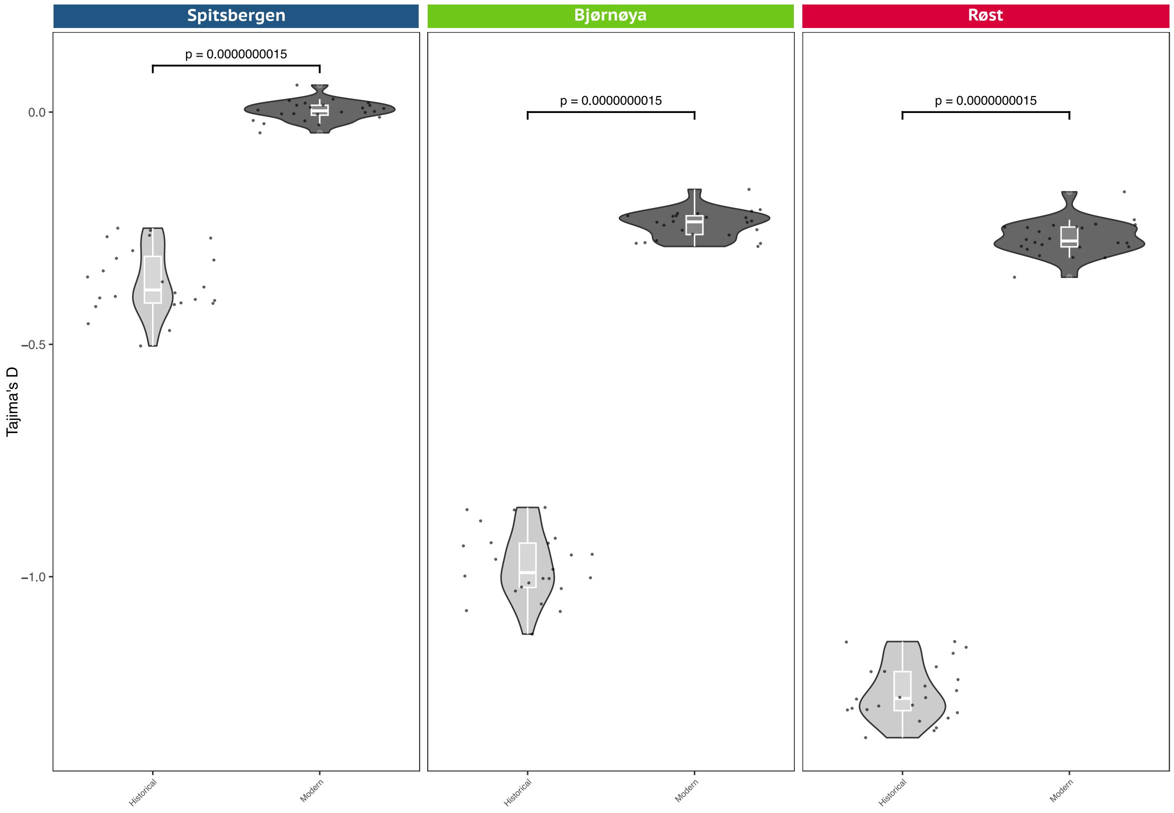

Fig. S16: Genome-wide Tajima's D compared between modern and historical Atlantic puffin colonies on Spitsbergen, Bjørnøya and Røst. The dataset consisted of medium-coverage (5-10X) sequencing data. Estimates of Tajima's D were calculated in ANGSD with the per-population chromosome-based one-dimensional site frequency spectra. Error bars show the range of values within 1.5 times the interquartile range and white horizontal lines the median per population.

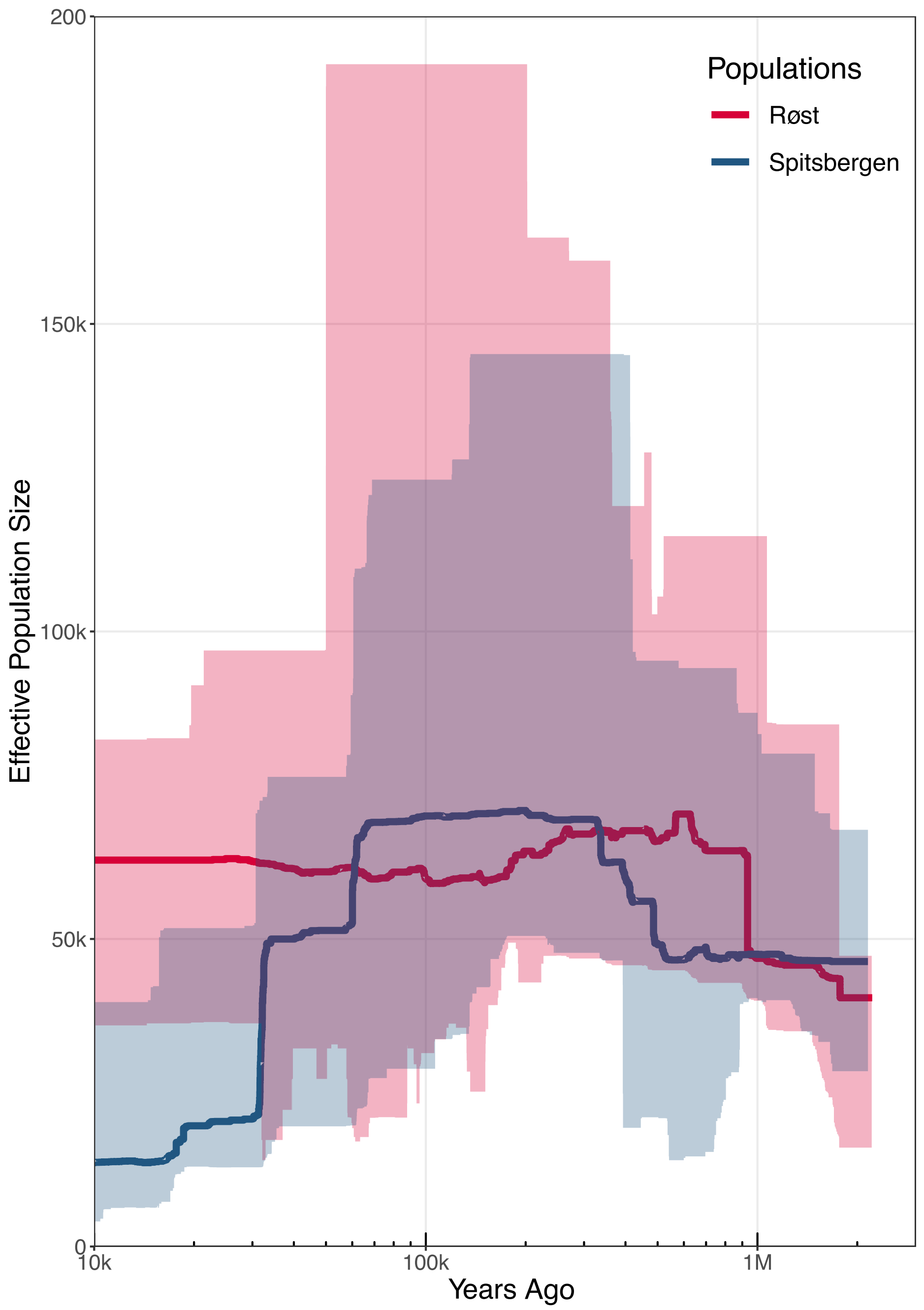

Fig. S17: Reconstruction of effective population size of two Atlantic puffin populations with *StairwayPlot2*. The demographic history was estimated using modern, high-coverage (>20X) sequencing data and spans the period of 10,000 years to 2 million years ago. Shaded area represents the 95% confidence interval.

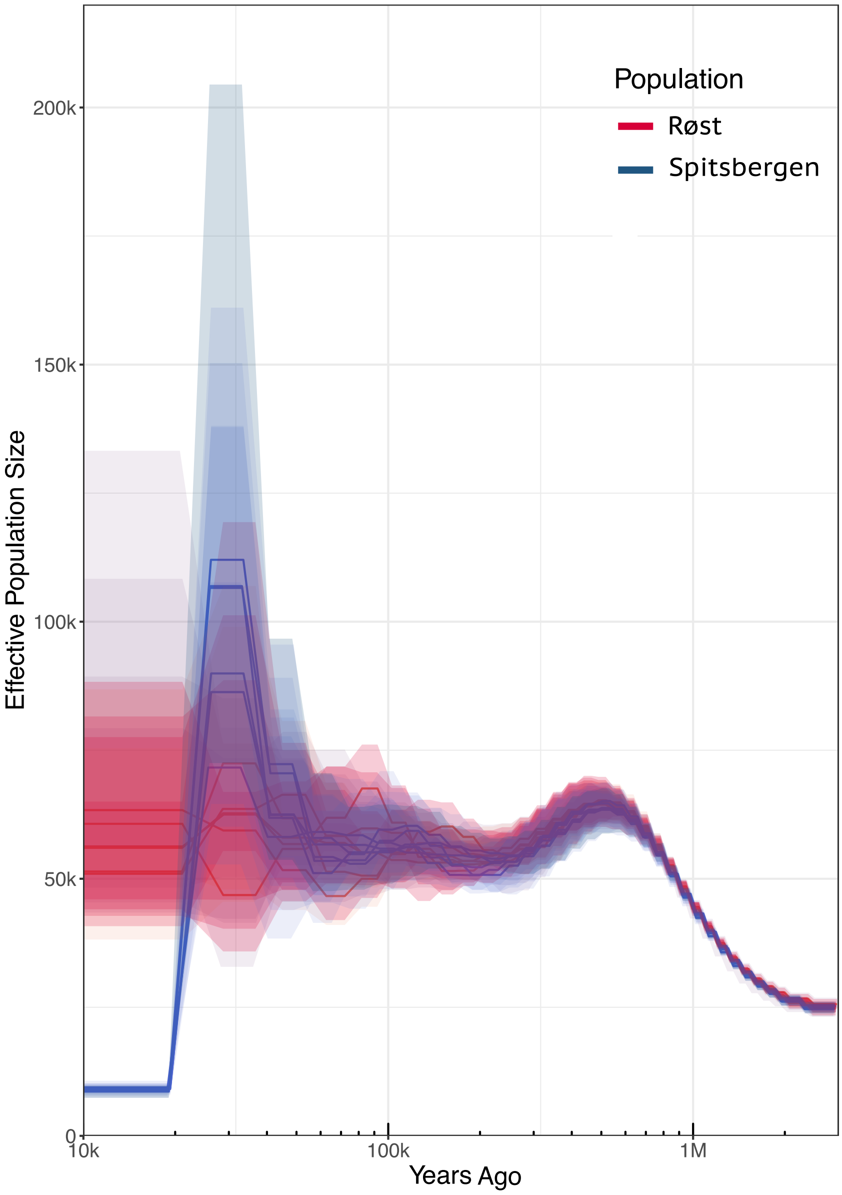

Fig. S18: Reconstruction of effective population size of two Atlantic puffin populations with the pairwise sequentially Markovian coalescent (*PSMC*). The demographic history was estimated using modern, high-coverage (>20X) sequencing data and spans the period of 10,000 years to 2 million years ago. Shaded area represents the 95% confidence interval of each individual per population.

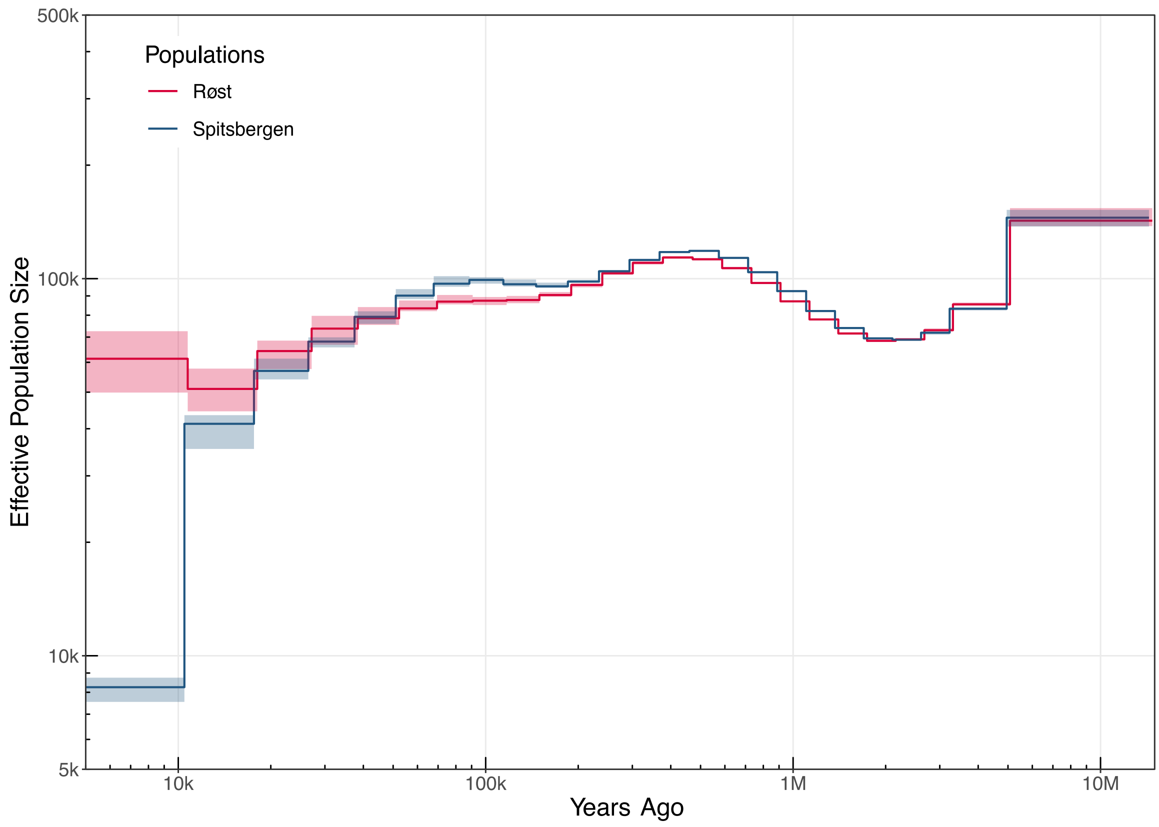

Fig. S19: Reconstruction of effective population size of two Atlantic puffin populations with the Markovian coalescent (*MSMC2*). The demographic history was estimate using modern, high-coverage (>20X) sequencing data and spans the period of 5,000 years to 2 million years ago. Shaded area represents total range of unique combinations of 2 haplotypes of 12 haplotypes within each population.

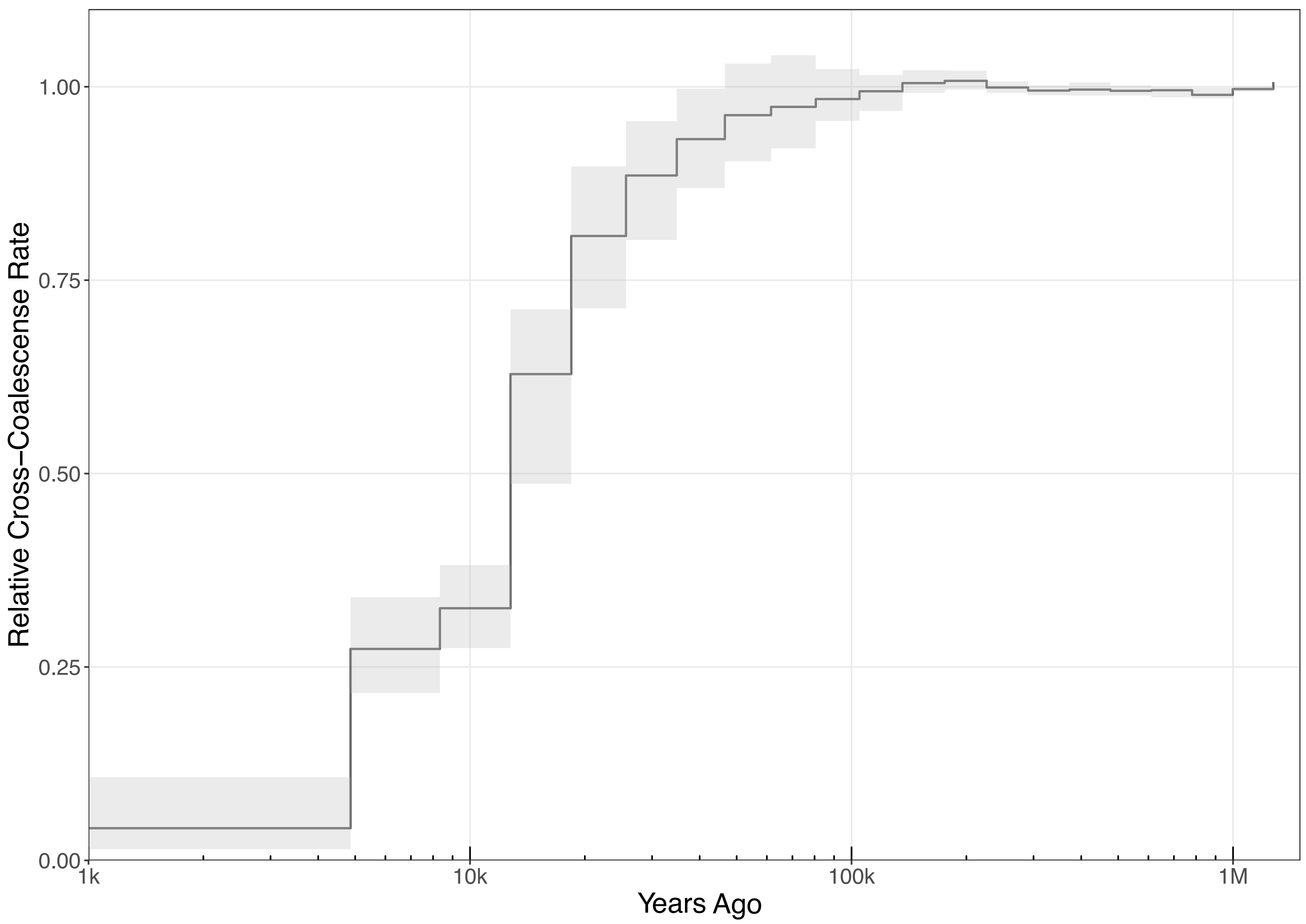

Fig. S20: Estimation of splitting time between two Atlantic puffin populations. The relative cross-coalescence rate as approximated by MSMC2 using modern, high-coverage (>20X) sequencing data and is used to evaluate the split time between Spitsbergen (*F. a. naumanni*) and Røst (*F. a. arctica*). Confidence intervals are represented by gray shading.

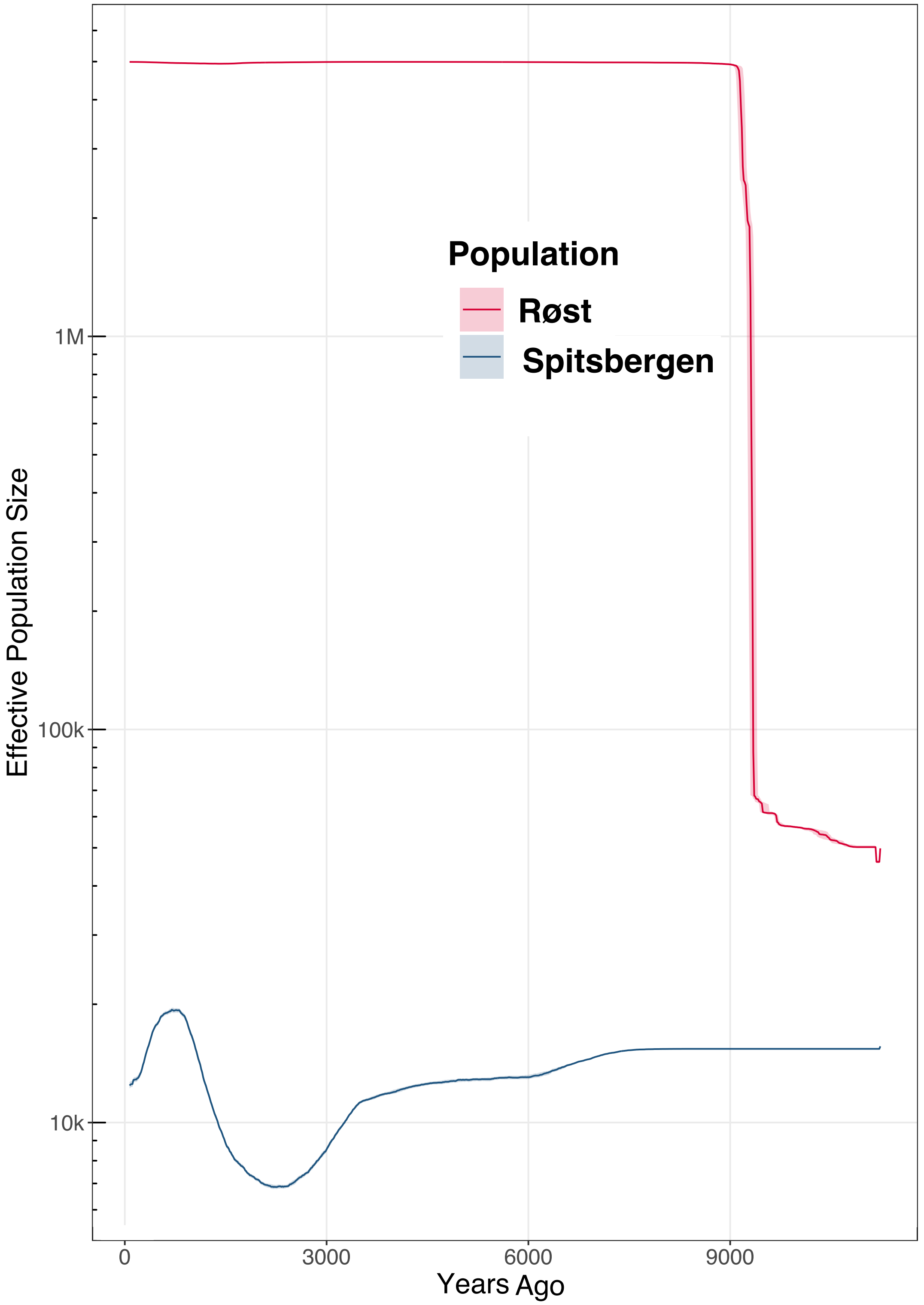

Fig. S21: Reconstruction of effective population size of two Atlantic puffin populations with *GONE*. The demographic history was estimated using contemporary, high-coverage (>20X) sequencing data and spans the period of 50 to 10,000 years ago. Shaded area represents 95% confidence intervals of 4000 bootstrap replicates.

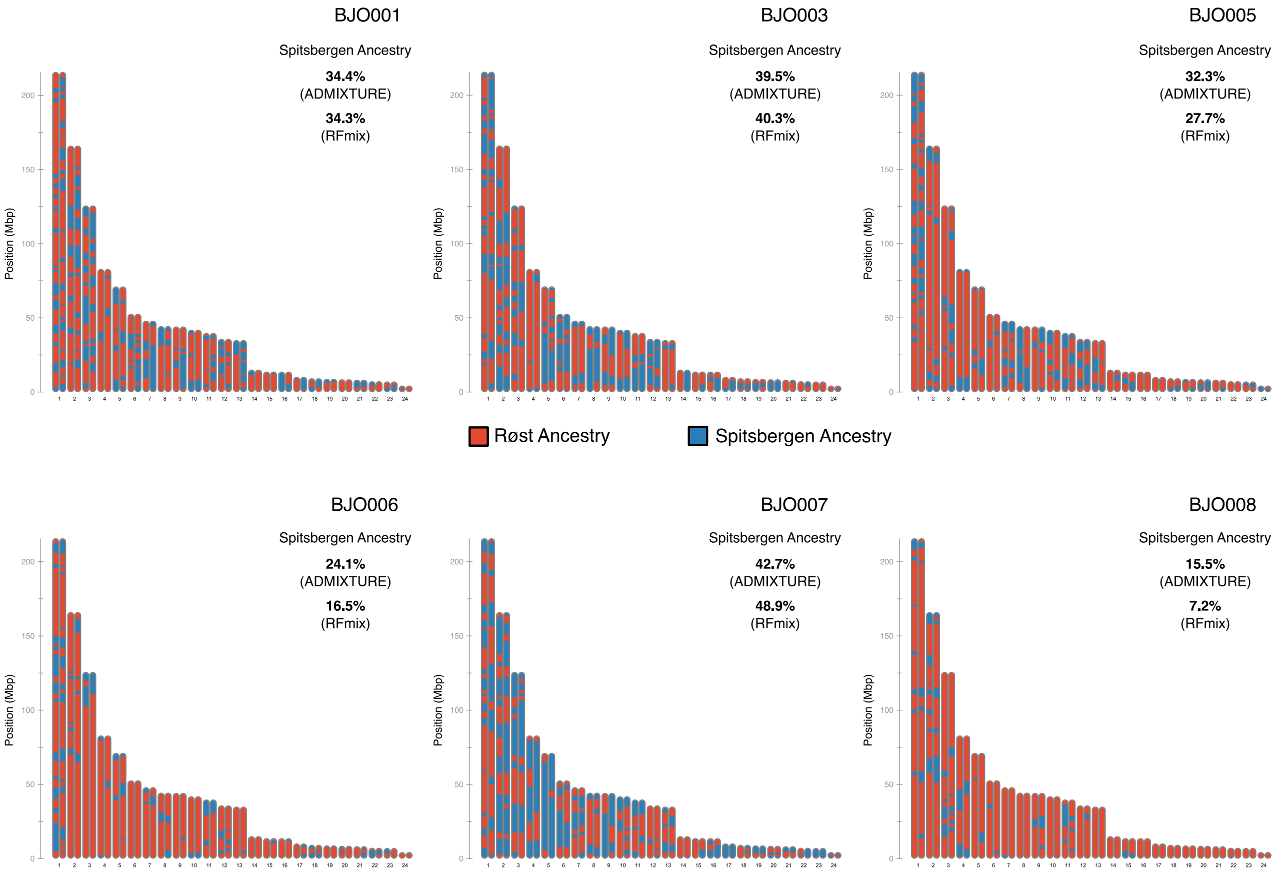

Figure S22: Ideograms of six Atlantic puffin hybrids from Bjørnøya. Plots were generated using high-coverage (>20X) sequencing data from modern individuals. Genomic regions were assigned to either Spitsbergen (*F. a. naumanni*) or Røst (*F. a.* arctica) ancestry across all 24 autosomes / 48 haplotypes using the program RFMix2. Global ancestry proportions were estimated with the programs ADMIXTURE and RFMix2.

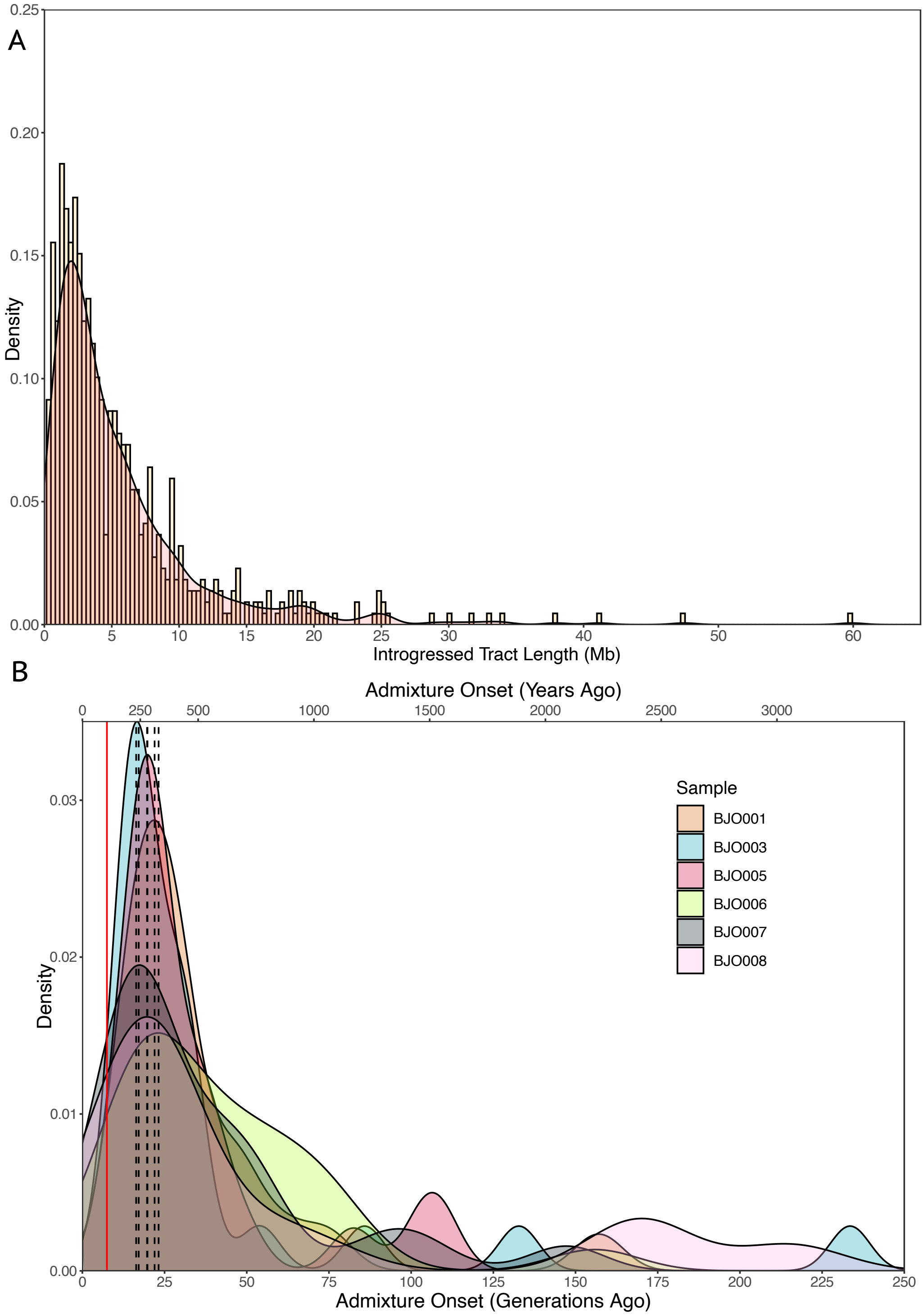

Figure S23: Distribution of length of *Fratercula arctica arctica* ancestry tracts and estimated onset of admixture for six Atlantic puffin hybrids from Bjørnøya. Analyses were conducted with high-coverage (>20X) sequencing data from modern individuals. A) Length distribution of genomic regions assigned to Spitsbergen (*F. a. naumanni*) ancestry across the 48 haplotypes of all 6 individuals using the program RFMix2. B) Distribution of estimated onset of admixture based on length of introgressed tracts, ancestry fraction, and recombination rate determined for each of the 6 individuals. Dashed lines represent introgression time with the maximum density distribution value. Red line depicts the time of sampling of historical Bjørnøya individuals which don't show any signs of hybridization.

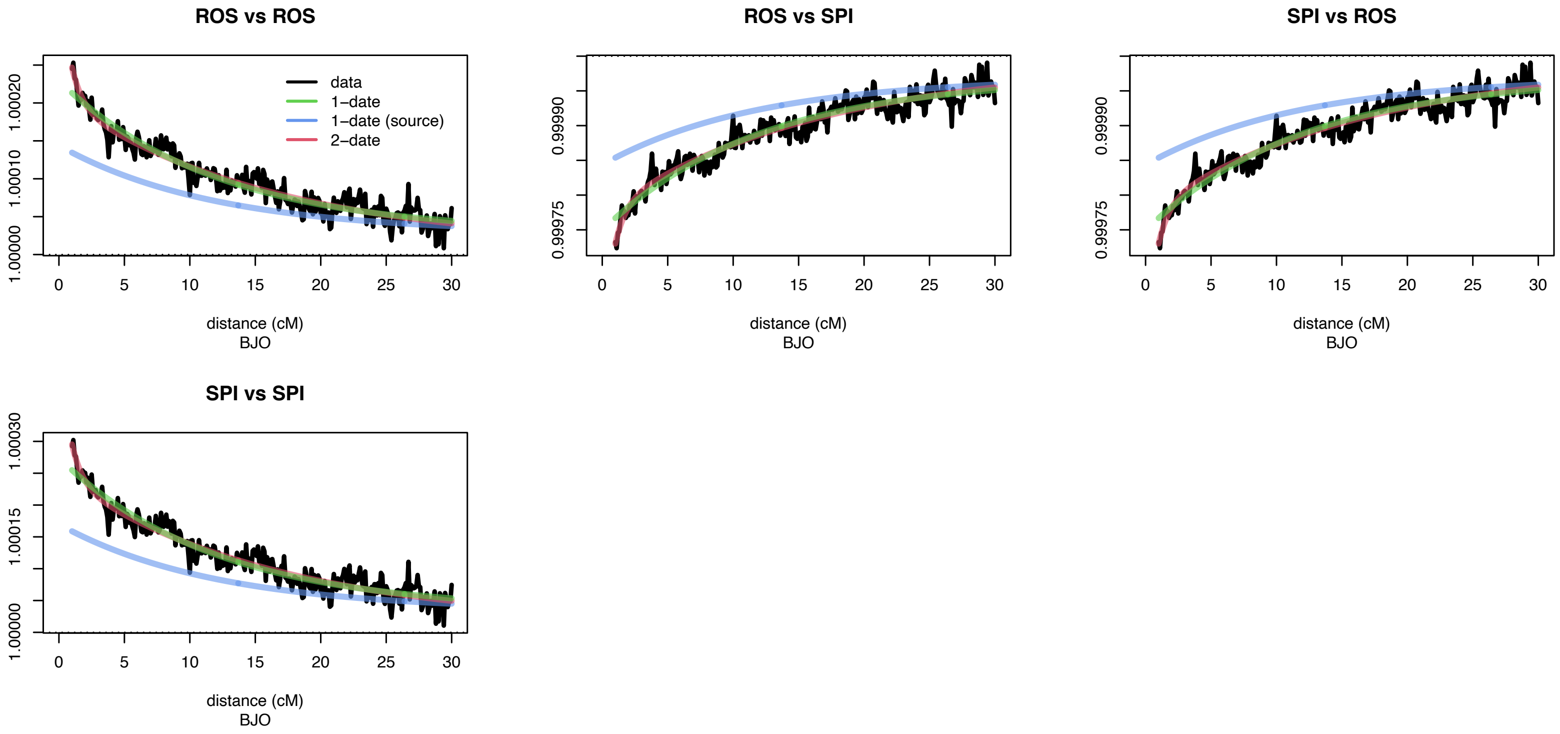

Figure S24: Model fit of different admixture scenarios between two Atlantic puffin subspecies as determined by fastGlobetrotter. Coancestry curves show the scaled probability (black lines) that two DNA segments in a target individual match to e.g. Røst (ROS) and Spitsbergen (SPI) surrogates (ROS vs SPI) for different lengths of separation (in cM). Decreasing curves suggest the two surrogates represent the same admixing source, while increasing curves suggest that the two surrogates represent different admixing sources. The green line provides the fit of a single date of admixture, while the red line gives the fit of two dates of admixture. The cyan line shows the fit of a single date of admixture between two sources. If the cyan line is not opposite to the black line (both increasing or decreasing), a single date of admixture is likely a good fit to the data.

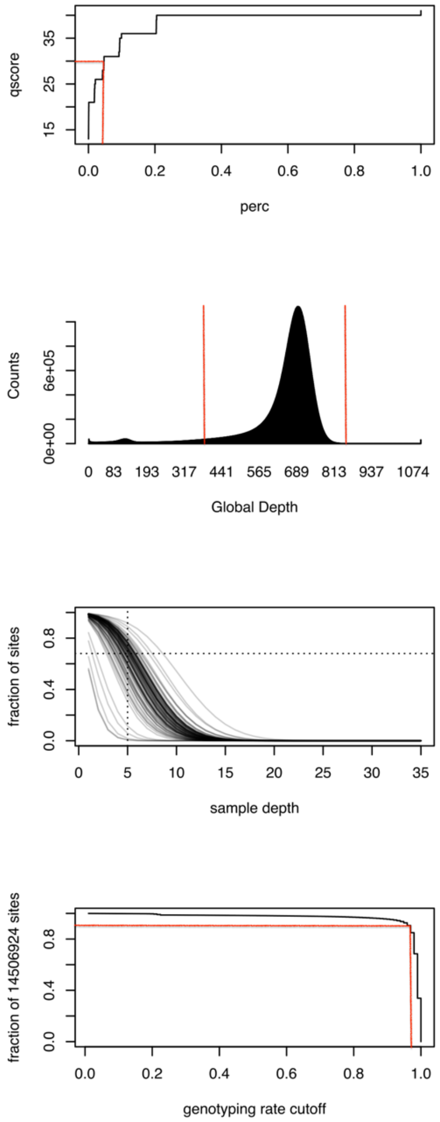

Figure S25: Mapping quality control of 98 Atlantic puffin samples aligned to the new reference genome. Samples consisted of medium-coverage (5-10X) sequencing data. Cutoffs for genotyping rate, global depth and QScore (red lines) were chosen for downstream genotype likelihood calculations.

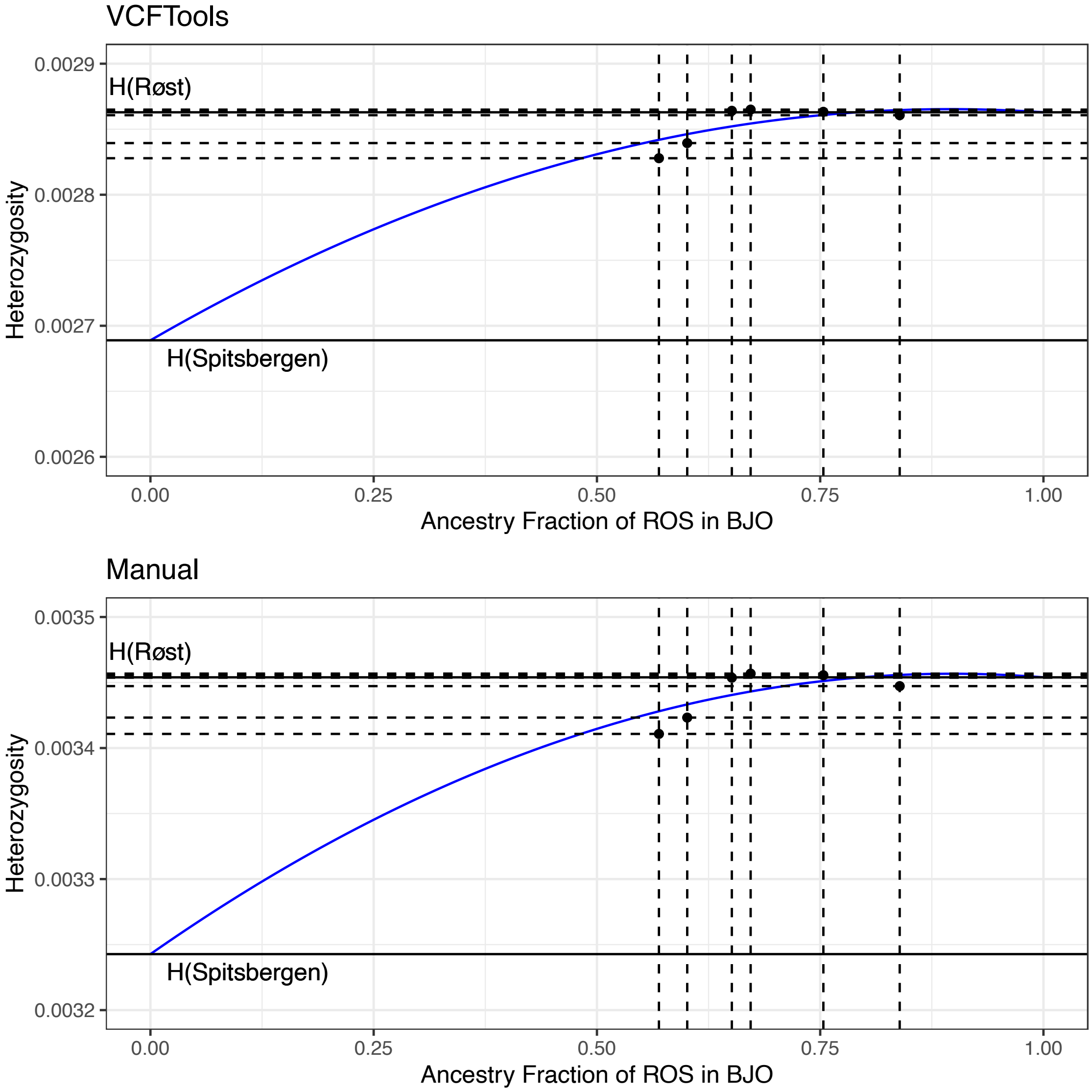

Figure S26: Expected and empirical heterozygosity of modern Bjørnøya individuals. Expected levels of heterozygosity (blue line) were reconstructed using the theoretical framework presented in Boca et al. (*76*) combined with empirical values of F_{ST}, ancestry fraction and parental heterozygosity. The top panel uses levels of heterozygosity generated by VCFtools. In the bottom panel, heterozygosity was calculated as the proportion of heterozygous sites divided by total sites. H(Røst) and H(Spitsbergen) are the average heterozygosity of Røst and Spitsbergen, respectively. Black dots represent empirical heterozygosity values of each modern Bjørnøya sample (N=6).

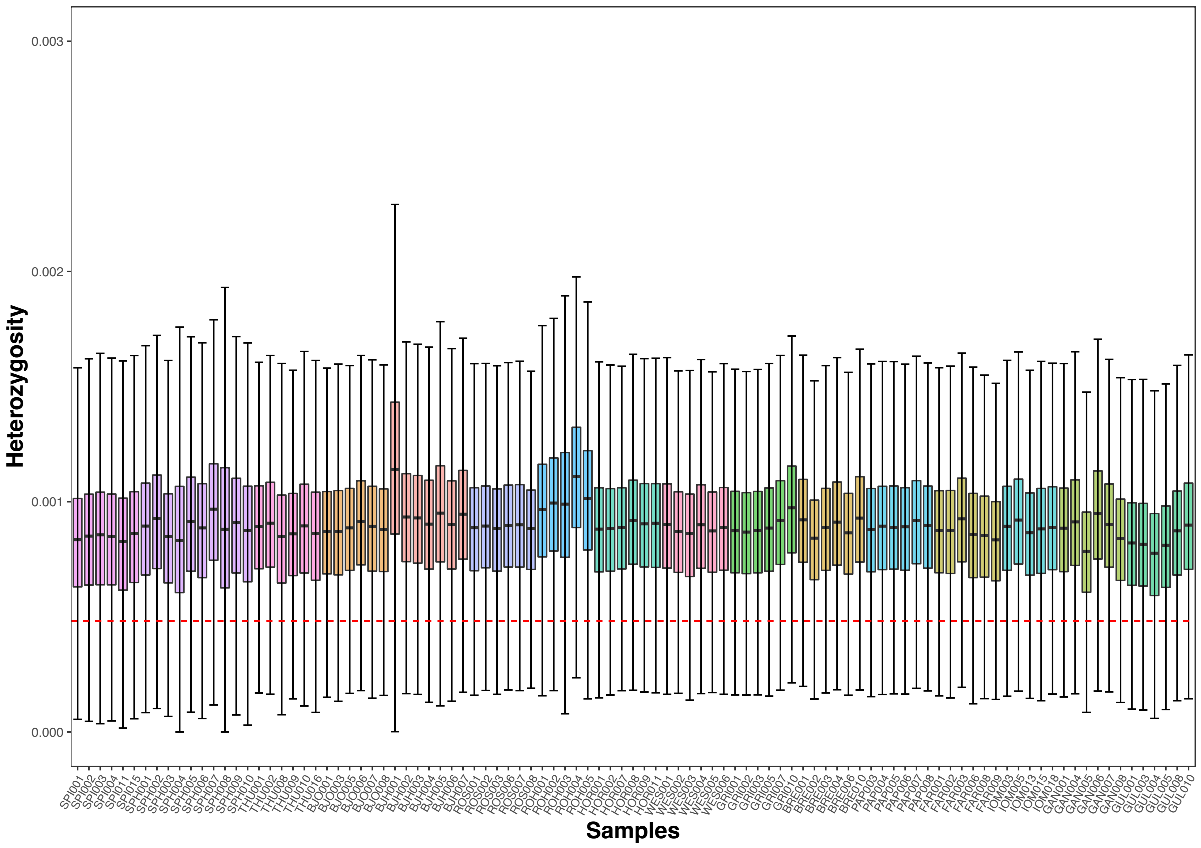

Figure S27: Local genome-wide heterozygosity compared between 98 historical and modern Atlantic puffin individuals across 13 colonies throughout the species' breeding range. The analysis was conducted with medium-coverage (5-10X) sequencing data from. Estimates of individual genome-wide heterozygosity are based on local estimates of heterozygosity in 100 kbp sliding windows with a 50 kbp shift along the 24 autosomes. Local estimates per window are based on one-dimensional Site Frequency Spectra calculated in ANGSD. The dashed red line indicates the 10% quantile of the average local heterozygosity across all samples (0.482×10^{-3}), which is used to determine the heterozygosity cutoff for high- or low-heterozygosity regions (*19*). Black lines present the median per sample. Error bars show range of values within 1.5 times the interquartile range. SPH=Spitsbergen (Historical), SPI=Spitsbergen (Modern), THU=Thule, BJO=Bjørnøya (Modern), BJH=Bjørnøya (Historical), HOR=Hornøya, ROS=Røst (Modern), ROH=Røst (Historical), WES=Vestmannaeyjar, PAP=Papey, BRE=Breiðafjörður, GRI=Grímsey, FAR=Faroe Isl., IOM=Isle of May, GAN=Gannet Isl., GUL=Gull Isl.

Table S1: Comparison of assembly statistics between the newly generated, chromosome-level assembly and the previously available Atlantic Puffin reference genome. The previous reference genome was published in Kersten et al. (*19*).

Assembly	No. of Scaffolds	No. of Scaffolds >= 1 kbp	No. of Scaffolds >= 5 kbp	No. of Scaffolds >= 10 kbp	No. of Scaffolds >= 25 kbp	No. of Scaffolds >= 50 kbp	Max. Scaffold Size (Mbp)
Kersten et al. (*19*)	15,329	15,124	5,500	2,545	1,446	898	28.83
This study	255	252	177	138	104	64	215.51

Assembly	N50 (Mbp)	L50	N75 (Mbp)	L75	GC content (%)	No. of N's per 100 kbp	Genome Lenght (Gbp)
Kersten et al. (*19*)	6.09	61	2.17	153	41.98	10,052	1.2938
This study	82.41	5	43.60	10	42.75	14	1.2150

Assembly	BUSCO				
	Complete Genes Total (%)	Single Copy Genes (%)	Duplicated Genes (%)	Fragmented Genes (%)	Missing Genes (%)
Kersten et al. (*19*)	92.00	90.91	1.10	4.68	3.32
This study	97.36	96.68	0.68	0.58	2.06

Table S2: Final assembly and annotation statistics of the chromosome-level Atlantic Puffin reference genome. CDS=Coding DNA Sequence. Transposable Elements Other = satellites, simple repeats, low complexity.

Chr	Length	#A	#C	#G	#T	#N	#CpG
Chr_01	215,510,477	63,715,434	44,241,367	44,084,502	63,450,984	18,190	4,861,998
Chr_02	165,692,682	49,119,029	33,753,928	33,728,740	49,077,401	13,584	3,757,528
Chr_03	125,303,252	36,777,377	25,650,051	25,806,712	37,062,452	6,660	2,968,612
Chr_04	82,413,236	24,562,561	16,846,850	16,687,934	24,311,380	4,511	2,038,982
Chr_05	70,650,776	20,482,553	14,856,155	14,832,114	20,477,062	2,892	1,903,990
Chr_06	52,107,066	14,556,375	11,379,360	11,484,662	14,678,633	8,036	1,703,076
Chr_07	47,495,801	13,472,567	10,244,437	10,270,924	13,502,226	5,647	1,502,512
Chr_08	43,806,797	12,690,662	9,150,066	9,204,538	12,758,468	3,063	1,231,452
Chr_09	43,604,671	12,165,449	9,642,933	9,631,579	12,161,025	3,685	1,486,888
Chr_10	41,382,176	11,927,415	8,732,640	8,770,166	11,949,576	2,379	1,197,698
Chr_11	39,203,813	10,828,060	8,834,763	8,786,243	10,750,585	4,162	1,400,254
Chr_12	35,224,132	10,156,885	7,521,268	7,461,704	10,083,182	1,093	1,081,318
Chr_13	34,399,331	9,309,108	7,898,852	7,895,325	9,290,372	5,674	1,297,878
Chr_14	14,439,572	2,703,475	4,510,313	4,480,257	2,712,035	33,492	1,213,380
Chr_15	13,134,562	3,515,538	3,128,655	3,066,605	3,423,266	498	591,428
Chr_16	13,092,466	3,360,487	3,182,294	3,172,990	3,375,499	1,196	611,588
Chr_17	9,551,467	2,514,538	2,280,264	2,270,651	2,485,914	100	456,542
Chr_18	8,612,993	2,110,836	2,198,551	2,192,754	2,110,262	590	477,350
Chr_19	8,228,053	2,077,753	2,023,760	2,039,257	2,085,595	1,688	458,290
Chr_20	7,930,570	1,863,905	2,059,914	2,088,627	1,913,812	4,312	479,868
Chr_21	7,665,519	1,817,113	2,015,890	2,002,532	1,824,822	5,162	478,630
Chr_22	6,613,858	1,526,094	1,763,062	1,784,546	1,538,164	1,992	444,792
Chr_23	6,449,071	1,596,296	1,598,760	1,626,154	1,626,278	1,583	364,828
Chr_24	3,686,483	780,877	1,073,811	1,058,107	772,489	1,199	308,930
Chr_W	28,376,909	7,859,868	6,309,183	6,279,613	7,902,411	25,834	800,352
Chr_Z	83,993,355	24,870,473	17,101,584	17,110,913	24,906,381	4,004	1,808,628
Chr_MT	19,084	5,911	5,888	2,622	4,663	-	1,004
Chr_Unplaced	6,478,410	1,455,134	1,684,528	1,807,249	1,478,776	52,723	432,948

No. of unplaced scaffolds	229
Length of unplaced scaffolds (without N padding)	6,112,139
Median length of unplaced scaffolds	9,751
Proportion of combined length of unplaced scaffolds vs. Chromosomes	2.371%

Stats Summary (Quast + Gfastats)*	**No. of contigs**	**Total contig length**	**Average contig length**	**Contig N50**	**Contig L50**	**Largest contig**	**Smallest contig**
	757	1,214,833,549	1,604,800	16,421,795	24	52,759,211	1
	**No. of scaffolds**	**Total scaffold length**	**Average scaffold length**	**Scaffold N50**	**Scaffold L50**	**Largest scaffold**	**Smallest scaffold**
	27	1,215,047,498	45,001,759	82,413,236	5	215,510,477	3,686,483
	**No. of gaps in scaffolds**	**Total gap length in scaffolds**	**Average gap length in scaffolds**	**No. of N's per 100 kbp**	**Largest gap in scaffolds**	**GC content %**	**No. of soft-masked bases**
	731	213,949	293	14	596	42.75	732,728

**BUSCO**	8,118	**Complete BUSCOs**	97.36%
	8,061*	**Complete and single-copy BUSCOs**	96.68%**
	57	**Complete and duplicated BUSCOs**	0.68%
	48	**Fragmented BUSCOs**	0.58%
	172	**Missing BUSCOs**	2.06%
	8,338	**Total BUSCO groups searched**	

		Percent (%)	Total Length (Mb)
**Transposable Elements**	**DNA**	0.41	5.04
	**LINE**	5.65	68.67
	**SINE**	0.13	1.57
	**LTR**	1.71	20.81
	**Small RNA**	0.01	0.14
	**Other**	1.38	16.82
	**Unknown**	0.68	8.23
	**Total**	9.97	121.28

**Annotated Protein Coding Genes**	**No. Of Predicted Genes**	15,523
	**Mean CDS Length (bp)**	2,316
	**Mean Exon Length (bp)**	282
	**Mean Intron Length (bp)**	4,088
	**Complete BUSCOs**	8,229 (98.7%)

**Merqury**	**QV score**	37.63
	**Error Rate**	0.017 %
	**K-mer completeness**	90.78 %

*Nuclear genome only

**One single-copy BUSCO gene is located on the unplaced scaffold(s) sequence

Table S3: Significant admixture signals among three modern Atlantic puffin colonies. A) $f3$-statistics were calculated in Treemix and comparisons that have a Z-score < -3 provide evidence of admixture between population A and B in population ”Admixed”. B) D-statistics were calculated with Dsuite and an excess of ABBA sites leads to a significant gene-flow signal between P2 and P3 indicative of admixture in the topology (P1, P2), P3), Outgroup).

A

Admixed	A	B	f3	Zscore	stdErr
Bjørnøya	Spitsbergen	Røst	-0.00251	-46.24	0.000054
Røst	Bjørnøya	Spitsbergen	0.00281	46.04	0.000061
Spitsbergen	Bjørnøya	Spitsbergen	0.00601	72.40	0.000083

B

P1	P2	P3	Dstatistic	Z-score	p-value	f4-ratio	BBAA	ABBA	BABA	Outgroup
Røst	Bjørnøya	Spitsbergen	0.02739	29.54	0	0.296795	457,715	478,964	453,429	Razorbill

Table S4: Differences in genomic differentiation (F_{ST}), nucleotide diversity (Pi), Tajima's D and genome-wide heterozygosity between three modern Atlantic puffin colonies. Statistical significance was assessed with a Dunn test combined with a Holm correction accounting for multiple testing.

Statistic	Population 1	Population 2	N (Pop1)	N (Pop2)	Dunn Test					Holm Correction
					estimate	estimate1	estimate2	statistic	p	p.adj
Fst	Røst vs. Bjørnøya	Spitsbergen vs. Bjørnøya	43756	43755	11,244.51	52,158.82	63,403.33	43.89	0.00000	0.00000
	Røst vs. Bjørnøya	Spitsbergen vs. Røst	43756	43756	29,180.98	52,158.82	81,339.80	113.90	0.00000	0.00000
	Spitsbergen vs. Bjørnøya	Spitsbergen vs. Røst	43755	43756	17,936.48	63,403.33	81,339.80	70.01	0.00000	0.00000
Pi	Spitsbergen	Bjørnøya	43758	43758	6,552.19	61,004.75	67,556.95	25.59	0.00000	0.00000
	Spitsbergen	Røst	43758	43758	7,346.05	61,004.75	68,350.80	28.69	0.00000	0.00000
	Bjørnøya	Røst	43758	43758	793.85	67,556.95	68,350.80	3.10	0.00194	0.00194
Tajima's D	Spitsbergen	Bjørnøya	43754	43755	-27,609.13	85,066.98	57,457.85	-107.77	0.00000	0.00000
	Spitsbergen	Røst	43754	43756	-30,692.10	85,066.98	54,374.88	-119.80	0.00000	0.00000
	Bjørnøya	Røst	43755	43756	-3,082.97	57,457.85	54,374.88	-12.03	0.00000	0.00000
Heterozygosity	Bjørnøya	Røst	6	6	1.67	10.67	12.33	0.57	0.56755	0.56755
	Bjørnøya	Spitsbergen	6	5	-7.67	10.67	3.00	-2.51	0.01217	0.02433
	Røst	Spitsbergen	6	5	-9.33	12.33	3.00	-3.05	0.00227	0.00681

Table S5: Average genome-wide nucleotide diversity (pi) and Tajima's D across modern and historical Atlantic puffin populations and genomic clusters. Pi and Tajima's D are based on folded one-dimensional site frequency spectra and were calculated per individual in ANGSD. SD=standard deviation.

Colony	Mean Pi (+- SD)		Mean Tajima's D (+- SD)	
Spitsbergen (Modern)	1.00	(0.086)	0.003	(8.6e-05)
Spitsbergen (Historical)	1.08	(0.084)	-0.360	(8.4e-05)
Thule (Modern)	1.02	(0.080)	-0.037	(8.0e-05)
Bjørnøya (Modern)	1.07	(0.086)	-0.240	(8.6e-05)
Bjørnøya (Historical)	1.35	(0.110)	-0.980	(1.1e-04)
Røst (Modern)	1.08	(0.090)	-0.270	(9.0e-05)
Røst (Historical)	2.06	(0.250)	-1.300	(2.5e-04)
Nor/Ice/Far (Modern)	1.12	(0.100)	-0.580	(1.0e-04)
UK (Modern)	1.07	(0.094)	-0.210	(9.4e-05)
Canada (Modern)	1.11	(0.093)	-0.340	(9.3e-05)

Table S6: Differences in nucleotide diversity (pi) between modern and historical Atlantic puffin populations and genomic clusters. Pi is based on folded one-dimensional site frequency spectra and was calculated per individual in ANGSD. Statistical significance of differences was assessed with a Dunn Test with a Holm correction to account for multiple testing.

Population 1	Population 2	N (Pop1)	N (Pop2)	Dunn Test					Holm Correction
				estimate	estimate1	estimate2	statistic	p	p.adj
Bjørnøya (Historical)	Bjørnøya	24	24	-105.04	200.38	95.33	-5.24	1.60E-07	5.90E-06
	Canada	24	24	-76.04	200.38	124.33	-3.79	1.48E-04	4.59E-03
	UK	24	24	-101.46	200.38	98.92	-5.06	4.14E-07	1.45E-05
	Nor/Ice/Far	24	24	-71.13	200.38	129.25	-3.55	3.87E-04	1.12E-02
	Røst	24	24	-98.79	200.38	101.58	-4.93	8.25E-07	2.72E-05
	Spitsbergen (Historical)	24	24	-92.00	200.38	108.38	-4.59	4.42E-06	1.42E-04
	Spitsbergen (Modern)	24	24	-144.67	200.38	55.71	-7.22	5.26E-13	2.26E-11
	Thule	24	24	-137.75	200.38	62.63	-6.87	6.28E-12	2.64E-10
Røst (Historical)	Bjørnøya	24	24	-133.17	228.50	95.33	-6.64	3.04E-11	1.25E-09
	Canada	24	24	-104.17	228.50	124.33	-5.20	2.02E-07	7.27E-06
	UK	24	24	-129.58	228.50	98.92	-6.47	1.01E-10	4.03E-09
	Nor/Ice/Far	24	24	-99.25	228.50	129.25	-4.95	7.34E-07	2.49E-05
	Røst	24	24	-126.92	228.50	101.58	-6.33	2.41E-10	9.40E-09
	Spitsbergen (Historical)	24	24	-120.13	228.50	108.38	-5.99	2.05E-09	7.79E-08
	Spitsbergen (Modern)	24	24	-172.79	228.50	55.71	-8.62	6.60E-18	2.97E-16
	Thule	24	24	-165.88	228.50	62.63	-8.28	1.27E-16	5.58E-15
Spitsbergen (Modern)	Canada	24	24	68.63	55.71	124.33	3.42	6.17E-04	1.73E-02
Spitsbergen (Modern)	Nor/Ice/Far	24	24	73.54	55.71	129.25	3.67	2.43E-04	7.29E-03
Thule	Nor/Ice/Far	24	24	66.63	62.63	129.25	3.32	8.86E-04	2.39E-02

Table S7: Recombination rate approximations for each chromosome of the Atlantic Puffin reference genome. Rates were adopted from Table 5 of Kawakami et al. (*132*) and are based on the length of puffin chromosomes.

Chromosome	Recombination Rate (cM/Mb)
1-3	1.60
4-6	2.00
7-13	1.70
14-24	1.50
Whole Genome*	1.63

*Average across all chromosomes

Table S8: Date estimation and confidence intervals for two different admixture scenarios of Atlantic puffin subspecies as determined by fastGlobetrotter. FastGlobetrotter assesses two models, A) 1 admixture pulse and B) two admixture pulses throughout time, and determines onset of admixture timings, confidence intervals, admixture proportions and model fit.

A

1-Pulse Scenario							
	Date 1	95% CI			Max. R2 fit	Proportion Spitsbergen	Proportion Røst
Generations Ago	8.37	5.36	-	12.32	0.92	0.48	0.51
Years Ago	118.85	76.11	-	174.94			

B

2-Pulse Scenario											
	Date 1	95% CI			Date 2	95% CI			Max. Score	Proportion Spitsbergen	Proportion Røst
Generations Ago	6.17	1.00	-	10.51	119.97	14.75	-	831.02	0.10	0.48	0.51
Years Ago	87.61	14.20	-	149.24	1703.57	209.45	-	11800.48			

Table S9: Assembly, refinement and selection of the chromosome-level Atlantic Puffin reference genome assembly. Presented are the statistics for correctness and completeness of various assemblies along the assembly and curation process. The best assembly was "flye_salsa" and used for all downstream analyses.

Stats After Initial Assembly

Assembler	Total Length	No. Of Scaffolds	Length (Mean)	Longest	Shortest	No. Of N's	No. Of Gaps	N50	N50n	N70	N70n	N90	N90n
Falcon	1,228,201,305	993	1,236,859	68,113,552	16,808	-	-	15,416,812	23	7,202,605	45	806,692	153
Canu	1,361,051,256	4,108	331,317	59,894,332	1,017	-	-	13,024,781	28	4,786,146	63	107,455	449
Flye	1,226,286,544	1,021	1,201,064	122,910,341	484	5,400	54	35,218,231	10	20,777,190	19	4,840,738	42

Stats Before Manual Curation

Assembly	# contigs (>= 0 bp)	# contigs (>= 1000 bp)	# contigs (>= 5000 bp)	# contigs (>= 10000 bp)	# contigs (>= 25000 bp)	# contigs (>= 50000 bp)	Total length (>= 0 bp)	Total length (>= 1000 bp)	Total length (>= 5000 bp)	Total length (>= 10000 bp)	Total length (>= 25000 bp)	Total length (>= 50000 bp)	Largest contig	Total length	GC (%)	N50	N75	L50	L75	# N's per 100 kbp
falcon_allhic	558	550	461	432	343	232	1,224,809,502	1,224,801,703	1,224,597,062	1,224,389,087	1,222,813,087	1,219,047,998	215,245,371	1,224,809,502	43	75,700,947	43,341,121	5	10	24
falcon_salsa	828	824	670	591	440	255	1,221,881,344	1,221,877,356	1,221,556,394	1,220,981,328	1,218,306,979	1,211,907,921	215,090,119	1,221,881,344	43	71,396,321	43,734,089	5	10	29
flye_allhic	771	716	500	386	257	158	1,219,382,051	1,219,342,392	1,218,763,323	1,217,988,403	1,215,961,739	1,212,592,094	214,879,663	1,219,381,054	43	77,440,010	43,270,074	5	10	15
flye_salsa	1,110	1,033	653	466	259	140	1,225,197,164	1,225,141,315	1,224,111,199	1,222,798,391	1,219,610,796	1,215,612,698	212,852,945	1,225,195,681	43	79,207,774	43,478,921	5	10	14

BUSCO Before Manual Curation

Assembly	Total	Single	Duplicated	Fragmented	Missing	% Complete
falcon_allhic	8338	8021	75	54	188	97.10%
falcon_salsa	8338	7995	74	57	212	96.77%
flye_allhic	8338	8059	50	55	174	97.25%
flye_salsa	8338	8055	60	51	172	97.33%

Mitogenome

Assembly	Name	Length	#A	#C	#G	#T	#2	#3	Ns	#CpG	#tv	#ts	#CpG-ts
falcon_allhic	FraArc_MT	19,084	5,911	5,888	2,622	4,663	-	-	-	1,004	-	-	-
falcon_salsa	FraArc_MT	19,084	5,911	5,888	2,622	4,663	-	-	-	1,004	-	-	-
flye_allhic	FraArc_MT	19,084	5,911	5,888	2,622	4,663	-	-	-	1,004	-	-	-
flye_salsa	FraArc_MT	19,084	5,911	5,888	2,622	4,663	-	-	-	1,004	-	-	-

Dedup, Blobtools + Kraken2

Assembly	# of scaffolds after deduplication	No. of unclassified Blobtools	No. of unclassified Kraken2	No. of unclassified Both	No. of scaffolds after removal
falcon_allhic	283	58	56	36	247
falcon_salsa	372	105	101	70	302
flye_allhic	375	157	135	117	258
flye_salsa	489	241	221	175	314

Asset Evaluation

	ASSET V1										ASSET V2		COMBINED					
Assembly	No. Of Regions with good support	Total Length	% of Genome Length	No. Of Regions with low support	Total Length	% of Genome Length	Larger than 100kb	Total Length	Larger than 10kb	Total Length	No. Of Regions with low support	Length	No. Of Regions with low support	Length	Larger than 100kb	Total Length	Larger than 10kb	Total Length
falcon_allhic	5,541	1,198,521,067	99.16%	5,300	9,058,895	0.75%	-	-	167	3,400,391	1,091	6,596,027	2,103	4,776,628	-	-	104	1,910,676
falcon_salsa	4,742	1,196,090,278	99.27%	4,478	7,473,315	0.62%	-	-	132	2,484,288	1,060	5,968,956	1,995	4,099,780	-	-	77	1,291,901
flye_allhic	5,274	1,198,713,659	99.10%	5,018	9,523,911	0.79%	-	-	184	3,209,885	1,163	10,038,320	3,105	6,707,701	-	-	137	2,110,466
flye_salsa	5,318	1,203,335,958	99.04%	5,036	10,128,989	0.83%	-	-	200	3,558,284	1,177	10,564,437	3,092	7,028,992	-	-	150	2,380,557

Stats "Unplaced"

Assembly	No. of Chromosomes	Length of smallest chromosome	No. of Unplaced scaffolds	Length of longest Unplaced	Length of shortest Unplaced	No. of BUSCO hits on Unplaced	No. of Unplaced with BUSCO hit	No. of Unplaced with Complete BUSCO hit	No. of Unplaced with Duplicated BUSCO hit	No. of Unplaced with Fragmented BUSCO hit
falcon_allhic	27	1,121,338	220	676,758	1,000	87	59	50	1	10
falcon_salsa	27	1,598,496	275	264,161	1,000	106	63	56	1	15
flye_allhic	28	3,330,031	230	744,036	508	59	34	33	1	4
flye_salsa	28	2,001,467	286	320,471	508	59	34	27	2	8

Stats After Manual Curation

Assembly	# contigs (>= 0 bp)	# contigs (>= 1000 bp)	# contigs (>= 5000 bp)	# contigs (>= 10000 bp)	# contigs (>= 25000 bp)	# contigs (>= 50000 bp)	Total length (>= 0 bp)	Total length (>= 1000 bp)	Total length (>= 5000 bp)	Total length (>= 10000 bp)	Total length (>= 25000 bp)	Total length (>= 50000 bp)	Largest contig	Total length	GC (%)	N50	N75	L50	L75	# N's per 100 kbp
falcon_allhic	247	247	220	217	190	136	1,208,731,155	1,208,731,155	1,208,678,980	1,208,656,305	1,208,150,502	1,206,285,084	215,238,206	1,208,731,155	43	75,394,495	43,318,268	5	10	21
falcon_salsa	302	302	252	241	207	142	1,204,826,167	1,204,826,167	1,204,738,248	1,204,651,203	1,204,039,258	1,201,916,066	215,090,119	1,204,826,167	43	71,396,321	43,705,924	5	10	28
flye_allhic	258	256	227	205	162	112	1,209,649,149	1,209,647,742	1,209,579,428	1,209,428,413	1,208,710,695	1,207,026,626	214,864,044	1,209,649,149	43	77,440,010	43,270,074	5	10	13
flye_salsa	314	311	234	189	150	95	1,214,991,498	1,214,989,111	1,214,795,144	1,214,476,345	1,213,850,322	1,211,999,111	212,852,945	1,214,991,498	43	79,207,774	43,478,921	5	10	13

BUSCO After Manual Curation

Assembly	Total	Single	Duplicated	Fragmented	Missing	% Complete
falcon_allhic	8338	8029	65	55	189	97.07%
falcon_salsa	8338	8004	65	57	212	96.77%
flye_allhic	8338	8061	48	55	174	97.25%
flye_salsa	8338	8057	58	51	172	97.33%

Table S10: Single nucleotide polymorphism datasets used for different Atlantic puffin population genomic analyses. The contemporary genomic datasets (top 8) were generated mapping modern high-coverage (>20X) sequencing data and calling SNPs with GATK v4.2.0. The bottom two SNP datasets were generated mapping modern and historical medium-coverage (5-10X) sequencing data and calculating genotype likelihoods with ANGSD v0.935.

Name	No. Of Individuals	No. Of Sites	Analyses
NonLD-pruned	18	10,031,605	GONE
NonLD-pruned/NoRelatedInd	17	9,907,905	MSMC2(Phasing), f3 stats, RFMix2, Chromopainter2
LDPruned/NoRelatedInd	17	795,275	smartPCA, Admixture
NonVariant	18	998,750,827	StairwayPlot2, MSMC2(NonVariantSites)
NonVariant/NoRelatedInd	17	1,000,327,483	FST, Tajima's D, nucelotide diversity, heterozygosity, inbreeding
NonVariant/Outgroup	19	862,480,425	Mutation rate estimation
NonLD-pruned/NoRelatedInd/Outgroup	18	8,528,609	ABBABABA, f4-ratio
IBS/Outgroup*	19	343,369,191	Mutation rate estimation
Historical/Pruned	98	723,930	PCAngsd, ngsAdmix
Historical/SFS	98	674,422,394	Tajima's D, nucelotide diversity, heterozygosity, inbreeding

*Pseudohaploid genomes were generated with identity-by-sampling (IBS) consensus sampling with ANGSD v0.935

Table S11: Phasing performance of using WhatsHap alone versus WhatsHap followed by ShapeIt4 for nuclear Atlantic puffin genomes. The table shows the statistics for a randomly selected sample of the 18 genomes sequenced at an average depth of coverage of 21.6X.

Program	No. of Variants	Heterozygous Variants	Phased	Phased (%)	Unphased	Singletons	Blocks	Variants per Block (mean)	Variants per Block (min)	Variants per Block (max)	Bp per Block (mean)	Bp per Block (min)	Bp per Block (max)
WhatsHap	9,907,905	2,995,229	2,843,152	94.92	152,017	60	366,952	7.75	2	168	16,303.82	1	86,110,088
WhatsHap & Shapeit4	9,907,905	2,995,229	2,995,229	100.00	-	-	24	124,801.21	8,317	621,423	45,659,111.50	3,672,582	215,462,845

Data S1. (Separate file)
Sample metadata including information on location, sequencing output and ENA accession number for 77 modern and 22 historical Atlantic puffin individuals.

REFERENCES AND NOTES

1. C. Parmesan, Ecological and evolutionary responses to recent climate change. *Annu. Rev. Ecol. Evol. Syst.* **37**, 637–669 (2006).

2. E. S. Poloczanska, C. J. Brown, W. J. Sydeman, W. Kiessling, D. S. Schoeman, P. J. Moore, K. Brander, J. F. Bruno, L. B. Buckley, M. T. Burrows, C. M. Duarte, B. S. Halpern, J. Holding, C. V. Kappel, M. I. O'Connor, J. M. Pandolfi, C. Parmesan, F. Schwing, S. A. Thompson, A. J. Richardson, Global imprint of climate change on marine life. *Nat. Clim. Chang.* **3**, 919–925 (2013).

3. G. T. Pecl, M. B. Araújo, J. D. Bell, J. Blanchard, T. C. Bonebrake, I.-C. Chen, T. D. Clark, R. K. Colwell, F. Danielsen, B. Evengård, L. Falconi, S. Ferrier, S. Frusher, R. A. Garcia, R. B. Griffis, A. J. Hobday, C. Janion-Scheepers, M. A. Jarzyna, S. Jennings, J. Lenoir, H. I. Linnetved, V. Y. Martin, P. C. McCormack, J. McDonald, N. J. Mitchell, T. Mustonen, J. M. Pandolfi, N. Pettorelli, E. Popova, S. A. Robinson, B. R. Scheffers, J. D. Shaw, C. J. B. Sorte, J. M. Strugnell, J. M. Sunday, M.-N. Tuanmu, A. Vergés, C. Villanueva, T. Wernberg, E. Wapstra, S. E. Williams, Biodiversity redistribution under climate change: Impacts on ecosystems and human well-being. *Science* **355**, eaai9214 (2017).

4. M. Todesco, M. A. Pascual, G. L. Owens, K. L. Ostevik, B. T. Moyers, S. Hübner, S. M. Heredia, M. A. Hahn, C. Caseys, D. G. Bock, L. H. Rieseberg, Hybridization and extinction. *Evol. Appl.* **9**, 892–908 (2016).

5. J. Ottenburghs, The genic view of hybridization in the Anthropocene. *Evol. Appl.* **14**, 2342–2360 (2021).

6. C. J. Brauer, J. Sandoval-Castillo, K. Gates, M. P. Hammer, P. J. Unmack, L. Bernatchez, L. B. Beheregaray, Natural hybridization reduces vulnerability to climate change. *Nat. Clim. Chang.* 282–289 (2023).

7. M. C. Serreze, R. G. Barry, Processes and impacts of Arctic amplification: A research synthesis. *Glob. Planet Change* **77**, 85–96 (2011).

8. B. P. Kelly, A. Whiteley, D. Tallmon, The Arctic melting pot. *Nature* **468**, 891 (2010).

9. J. P. Colella, S. L. Talbot, C. Brochmann, E. B. Taylor, E. P. Hoberg, J. A. Cook, Conservation genomics in a changing arctic. *Trends Ecol. Evol.* **35**, 149–162 (2020).

10. S. A. Taylor, E. L. Larson, R. G. Harrison, Hybrid zones: Windows on climate change. *Trends Ecol. Evol.* **30**, 398–406 (2015).

11. K. Bi, T. Linderoth, D. Vanderpool, J. M. Good, R. Nielsen, C. Moritz, Unlocking the vault: Next-generation museum population genomics. *Mol. Ecol.* **22**, 6018–6032 (2013).

12. D. Nogués-Bravo, F. Rodríguez-Sánchez, L. Orsini, E. de Boer, R. Jansson, H. Morlon, D. A. Fordham, S. T. Jackson, Cracking the code of biodiversity responses to past climate change. *Trends Ecol. Evol.* **33**, 765–776 (2018).

13. E. L. Jensen, D. M. Leigh, Using temporal genomics to understand contemporary climate change responses in wildlife. *Ecol. Evol.* **12**, e9340 (2022).

14. J. D. Pongracz, D. Paetkau, M. Branigan, E. Richardson, Recent hybridization between a polar bear and grizzly bears in the Canadian Arctic. *Arctic* **70**, 151–160 (2017).

15. M. R. Jones, L. S. Mills, P. C. Alves, C. M. Callahan, J. M. Alves, D. J. R. Lafferty, F. M. Jiggins, J. D. Jensen, J. Melo-Ferreira, J. M. Good, Adaptive introgression underlies polymorphic seasonal camouflage in snowshoe hares. *Science* **360**, 1355–1358 (2018).

16. M. Paleczny, E. Hammill, V. Karpouzi, D. Pauly, Population trend of the world's monitored seabirds, 1950-2010. *PLOS ONE* **10**, e0129342 (2015).

17. Conservation of Arctic Flora and Fauna, "State of the Arctic Marine Biodiversity Report Update: Seabirds" (Conservation of Arctic Flora and Fauna International Secretariat, 2021).

18. S. Descamps, H. Strøm, As the Arctic becomes boreal: Ongoing shifts in a high-Arctic seabird community. *Ecology* **102**, e03485 (2021).

19. O. Kersten, B. Star, D. M. Leigh, T. Anker-Nilssen, H. Strøm, J. Danielsen, S. Descamps, K. E. Erikstad, M. G. Fitzsimmons, J. Fort, E. S. Hansen, M. P. Harris, M. Irestedt, O. Kleven, M. L. Mallory, K. S. Jakobsen, S. Boessenkool, Complex population structure of the Atlantic puffin revealed by whole genome analyses. *Commun. Biol.* **4**, 922 (2021).

20. M. P. Harris, S. Wanless, *The Puffin* (T & AD Poyser, Bloomsbury Publishing, 2011).

21. A. L. Fayet, R. Freeman, T. Anker-Nilssen, A. Diamond, K. E. Erikstad, D. Fifield, M. G. Fitzsimmons, E. S. Hansen, M. P. Harris, M. Jessopp, A.-L. Kouwenberg, S. Kress, S. Mowat, C. M. Perrins, A. Petersen, I. K. Petersen, T. K. Reiertsen, G. J. Robertson, P. Shannon, I. A. Sigurðsson, A. Shoji, S. Wanless, T. Guilford, Ocean-wide drivers of migration strategies and their influence on population breeding performance in a declining seabird. *Curr. Biol.* **27**, 3871–3878.e3 (2017).

22. BirdLife International, *Fratercula arctica. The IUCN Red List of Threatened Species 2017* (BirdLife International, 2017).

23. T. Anker-Nilssen, S. A. Hanssen, B. Moe, G. H. R. Systad, R. Barrett, J. O. Bustnes, S. Christensen-Dalsgaard, N. Dehnhard, S. Descamps, K. E. Erikstad, A. Follestad, M. Langset, K. Layton-Matthews, S. H. Lorentsen, E. Lorentzen, T. K. Reiertsen, H. Strøm, "Key-site monitoring in Norway 2021, including Svalbard and Jan Mayen," (Short Report no. 1–2022, SEAPOP, 2022).

24. K. Lilliendahl, E. S. Hansen, V. Bogason, M. Sigursteinsson, M. L. Magnúsdóttir, P. M. Jónsson, H. H. Helgason, G. J. Óskarsson, P. F. Óskarsson, Ó. J. Sigurðsson, Recruitment failure of Atlantic puffins *Fratercula arctica* and sandeels Ammodytes marinus in Vestmannaeyjar Islands. *Náttúrufræðingurinn* **83**, 65–79 (2013).

25. E. S. Hansen, H. Sandvik, K. E. Erikstad, N. G. Yoccoz, T. Anker-Nilssen, J. Bader, S. Descamps, K. Hodges, M. D. S. Mesquita, T. K. Reiertsen, Ø. Varpe, Centennial relationships between ocean temperature and Atlantic puffin production reveal shifting decennial trends. *Glob. Chang. Biol.* **27**, 3753–3764 (2021).

26. K. F. Drinkwater, The regime shift of the 1920s and 1930s in the North Atlantic. *Prog. Oceanogr.* **68**, 134–151 (2006).

27. D. M. Leigh, O. Kersten, B. Star, T. Anker-Nilssen, K. Burnham, J. Johnson, J. Provencher, S. Boessenkool, Sympatry of genetically distinct Atlantic Puffins (*Fratercula arctica*) in the High Arctic. *IBIS* **165**, 1022–1030 (2023).

28. N. Nakahama, Museum specimens: An overlooked and valuable material for conservation genetics. *Ecol. Res.* **36**, 13–23 (2021).

29. P. Wandeler, P. E. A. Hoeck, L. F. Keller, Back to the future: Museum specimens in population genetics. *Trends Ecol. Evol.* **22**, 634–642 (2007).

30. D. Díez-del-Molino, F. Sánchez-Barreiro, I. Barnes, M. T. P. Gilbert, L. Dalén, Quantifying temporal genomic erosion in endangered species. *Trends Ecol. Evol.* **33**, 176–185 (2018).

31. H. Alexanderson, M. Henriksen, H. T. Ryen, J. Y. Landvik, G. Peterson, 200 ka of glacial events in NW Svalbard: An emergence cycle facies model and regional correlations. *arktos* **4**, 1–25 (2018).

32. W. R. Farnsworth, L. Allaart, Ó. Ingólfsson, H. Alexanderson, M. Forwick, R. Noormets, M. Retelle, A. Schomacker, Holocene glacial history of Svalbard: Status, perspectives and challenges. *Earth Sci. Rev.* **208**, 103249 (2020).

33. G. Formenti, A. Rhie, J. Balacco, B. Haase, J. Mountcastle, O. Fedrigo, S. Brown, M. R. Capodiferro, F. O. Al-Ajli, R. Ambrosini, P. Houde, S. Koren, K. Oliver, M. Smith, J. Skelton, E. Betteridge, J. Dolucan, C. Corton, I. Bista, J. Torrance, A. Tracey, J. Wood, M. Uliano-Silva, K. Howe, S. McCarthy, S. Winkler, W. Kwak, J. Korlach, A. Fungtammasan, D. Fordham, V. Costa, S. Mayes, M. Chiara, D. S. Horner, E. Myers, R. Durbin, A. Achilli, E. L. Braun, A. M. Phillippy, E. D. Jarvis; Vertebrate Genomes Project Consortium, Complete vertebrate mitogenomes reveal widespread repeats and gene duplications. *Genome Biol.* **22**, 120 (2021).

34. J. C. Avise, *Phylogeography: The History and Formation of Species* (Harvard Univ. Press, 2000).

35. M. Skovrind, J. A. S. Castruita, J. Haile, E. C. Treadaway, S. Gopalakrishnan, M. V. Westbury, M. P. Heide-Jørgensen, P. Szpak, E. D. Lorenzen, Hybridization between two high Arctic cetaceans confirmed by genomic analysis. *Sci. Rep.* **9**, 7729 (2019).

36. T. Yamanouchi, Early 20th century warming in the Arctic: A review. *Polar Sci.* **5**, 53–71 (2011).

37. H. Tokinaga, S.-P. Xie, H. Mukougawa, Early 20th-century Arctic warming intensified by Pacific and Atlantic multidecadal variability. *Proc. Natl. Acad. Sci. U.S.A.* **114**, 6227–6232 (2017).

38. H. Løvenskiold, "Avifauna Svalbardensis: With a discussion on the geographical distribution of the birds in Spitsbergen and adjacent islands" (Skrifter nr. 129, Norsk Polarinstitut, 1964).

39. J. C. Coulson, A review of philopatry in seabirds and comparisons with other waterbird species. *Waterbirds* **39**, 229–240 (2016).

40. G. Beaugrand, P. C. Reid, Relationships between North Atlantic salmon, plankton, and hydroclimatic change in the Northeast Atlantic. *ICES J. Mar. Sci.* **69**, 1549–1562 (2012).

41. S. Descamps, T. Anker-Nilssen, R. T. Barrett, D. B. Irons, F. Merkel, G. J. Robertson, N. G. Yoccoz, M. L. Mallory, W. A. Montevecchi, D. Boertmann, Y. Artukhin, S. Christensen-Dalsgaard, K.-E. Erikstad, H. G. Gilchrist, A. L. Labansen, S.-H. Lorentsen, A. Mosbech, B. Olsen, A. Petersen, J.-F. Rail, H. M. Renner, H. Strøm, G. H. Systad, S. I. Wilhelm, L. Zelenskaya, Circumpolar dynamics of a marine top-predator track ocean warming rates. *Glob. Chang. Biol.* **23**, 3770–3780 (2017).

42. M. P. Harris, T. Anker-Nilssen, R. H. McCleery, K. E. Erikstad, D. N. Shaw, V. Grosbois, Effect of wintering area and climate on the survival of adult Atlantic puffins *Fratercula arctica* in the eastern Atlantic. *Mar. Ecol. Prog. Ser.* **297**, 283–296 (2005).

43. E. Thanou, S. Sponza, E. J. Nelson, A. Perry, S. Wanless, F. Daunt, S. Cavers, Genetic structure in the European endemic seabird, Phalacrocorax aristotelis, shaped by a complex

interaction of historical and contemporary, physical and nonphysical drivers. *Mol. Ecol.* **26**, 2796–2811 (2017).

44. J. A. Morris-Pocock, S. A. Taylor, T. P. Birt, M. Damus, J. F. Piatt, K. I. Warheit, V. L. Friesen, Population genetic structure in Atlantic and Pacific Ocean common murres (*Uria aalge*): Natural replicate tests of post-Pleistocene evolution. *Mol. Ecol.* **17**, 4859–4873 (2008).

45. K. Wojczulanis-Jakubas, A. Kilikowska, A. M. A. Harding, D. Jakubas, N. J. Karnovsky, H. Steen, H. Strøm, J. Welcker, M. Gavrilo, J. T. Lifjeld, A. Johnsen, Weak population genetic differentiation in the most numerous Arctic seabird, the little auk. *Polar Biol.* **37**, 621–630 (2014).

46. J. E. Thomas, G. R. Carvalho, J. Haile, N. J. Rawlence, M. D. Martin, S. Y. Ho, A. Þ. Sigfússon, V. A. Jósefsson, M. Frederiksen, J. F. Linnebjerg, J. A. S. Castruita, J. Niemann, M.-H. S. Sinding, M. Sandoval-Velasco, A. E. Soares, R. Lacy, C. Barilaro, J. Best, D. Brandis, C. Cavallo, M. Elorza, K. L. Garrett, M. Groot, F. Johansson, J. T. Lifjeld, G. Nilson, D. Serjeanston, P. Sweet, E. Fuller, A. K. Hufthammer, M. Meldgaard, J. Fjeldså, B. Shapiro, M. Hofreiter, J. R. Stewart, M. T. P. Gilbert, M. Knapp, Demographic reconstruction from ancient DNA supports rapid extinction of the great auk. *eLife* **8**, e47509 (2019).

47. J. R. Stewart, A. M. Lister, I. Barnes, L. Dalén, Refugia revisited: Individualistic responses of species in space and time. *Proc. Biol. Sci.* **277**, 661–671 (2010).

48. J. Y. Landvik, E. J. Brook, L. Gualtieri, G. Raisbeck, O. Salvigsen, F. Yiou, Northwest Svalbard during the last glaciation: Ice-free areas existed. *Geology* **31**, 905–908 (2003).

49. I. Newton, *Speciation and Biogeography of Birds* (Academic Press, 2003).

50. A. Tigano, M. Damus, T. P. Birt, J. A. Morris-Pocock, Y. B. Artukhin, V. L. Friesen, The Arctic: Glacial refugium or area of secondary contact? Inference from the population genetic structure of the thick-Billed Murre (Uria lomvia), with implications for management. *J. Hered.* **106**, 238–246 (2015).

51. T. Moum, E. Arnason, Genetic diversity and population history of two related seabird species based on mitochondrial DNA control region sequences. *Mol. Ecol.* **10**, 2463–2478 (2001).

52. G. Caughley, Directions in conservation biology. *J. Anim. Ecol.* **63**, 215–244 (1994).

53. Y. S. Cho, L. Hu, H. Hou, H. Lee, J. Xu, S. Kwon, S. Oh, H.-M. Kim, S. Jho, S. Kim, Y.-A. Shin, B. C. Kim, H. Kim, C.-U. Kim, S.-J. Luo, W. E. Johnson, K.-P. Koepfli, A. Schmidt-Küntzel, J. A. Turner, L. Marker, C. Harper, S. M. Miller, W. Jacobs, L. D. Bertola, T. H. Kim, S. Lee, Q. Zhou, H.-J. Jung, X. Xu, P. Gadhvi, P. Xu, Y. Xiong, Y. Luo, S. Pan, *C. Gou*, X. Chu, J. Zhang, S. Liu, J. He, Y. Chen, L. Yang, Y. Yang, J. He, S. Liu, J. Wang, C. H. Kim, H. Kwak, J.-S. Kim, S. Hwang, J. Ko, C.-B. Kim, S. Kim, D. Bayarlkhagva, W. K. Paek, S.-J. Kim, S. J. O'Brien, J. Wang, J. Bhak, The tiger genome and comparative analysis with lion and snow leopard genomes. *Nat. Commun.* **4**, 2433 (2013).

54. J. Prado-Martinez, P. H. Sudmant, J. M. Kidd, H. Li, J. L. Kelley, B. Lorente-Galdos, K. R. Veeramah, A. E. Woerner, T. D. O'Connor, G. Santpere, A. Cagan, C. Theunert, F. Casals, H. Laayouni, K. Munch, A. Hobolth, A. E. Halager, M. Malig, J. Hernandez-Rodriguez, I. Hernando-Herraez, K. Prüfer, M. Pybus, L. Johnstone, M. Lachmann, C. Alkan, D. Twigg, N. Petit, C. Baker, F. Hormozdiari, M. Fernandez-Callejo, M. Dabad, M. L. Wilson, L. Stevison, C. Camprubí, T. Carvalho, A. Ruiz-Herrera, L. Vives, M. Mele, T. Abello, I. Kondova, R. E. Bontrop, A. Pusey, F. Lankester, J. A. Kiyang, R. A. Bergl, E. Lonsdorf, S. Myers, M. Ventura, P. Gagneux, D. Comas, H. Siegismund, J. Blanc, L. Agueda-Calpena, M. Gut, L. Fulton, S. A. Tishkoff, J. C. Mullikin, R. K. Wilson, I. G. Gut, M. K. Gonder, O. A. Ryder, B. H. Hahn, A. Navarro, J. M. Akey, J. Bertranpetit, D. Reich, T. Mailund, M. H. Schierup, C. Hvilsom, A. M. Andrés, J. D. Wall, C. D. Bustamante, M. F. Hammer, E. E. Eichler, T. Marques-Bonet, Great ape genetic diversity and population history. *Nature* **499**, 471–475 (2013).

55. M. H. Kohn, W. J. Murphy, E. A. Ostrander, R. K. Wayne, Genomics and conservation genetics. *Trends Ecol. Evol.* **21**, 629–637 (2006).

56. K. Theissinger, C. Fernandes, G. Formenti, I. Bista, P. R. Berg, C. Bleidorn, A. Bombarely, A. Crottini, G. R. Gallo, J. A. Godoy, S. Jentoft, J. Malukiewicz, A. Mouton, R. A. Oomen, S. Paez, P. J. Palsbøll, C. Pampoulie, M. J. Ruiz-López, S. Secomandi, H. Svardal, C. Theofanopoulou, J.

de Vries, A.-M. Waldvogel, G. Zhang, E. D. Jarvis, M. Bálint, C. Ciofi, R. M. Waterhouse, C. J. Mazzoni, J. Höglund; European Reference Genome Atlas Consortium, How genomics can help biodiversity conservation. *Trends Genet.* **39**, 545–559 (2023).

57. A. J. Chunco, Hybridization in a warmer world. *Ecol. Evol.* **4**, 2019–2031 (2014).

58. A. Rhie, S. A. McCarthy, O. Fedrigo, J. Damas, G. Formenti, S. Koren, M. Uliano-Silva, W. Chow, A. Fungtammasan, J. Kim, C. Lee, B. J. Ko, M. Chaisson, G. L. Gedman, L. J. Cantin, F. Thibaud-Nissen, L. Haggerty, I. Bista, M. Smith, B. Haase, J. Mountcastle, S. Winkler, S. Paez, J. Howard, S. C. Vernes, T. M. Lama, F. Grutzner, W. C. Warren, C. N. Balakrishnan, D. Burt, J. M. George, M. T. Biegler, D. Iorns, A. Digby, D. Eason, B. Robertson, T. Edwards, M. Wilkinson, G. Turner, A. Meyer, A. F. Kautt, P. Franchini, H. W. Detrich 3rd, H. Svardal, M. Wagner, G. J. P. Naylor, M. Pippel, M. Malinsky, M. Mooney, M. Simbirsky, B. T. Hannigan, T. Pesout, M. Houck, A. Misuraca, S. B. Kingan, R. Hall, Z. Kronenberg, I. Sović, C. Dunn, Z. Ning, A. Hastie, J. Lee, S. Selvaraj, R. E. Green, N. H. Putnam, I. Gut, J. Ghurye, E. Garrison, Y. Sims, J. Collins, S. Pelan, J. Torrance, A. Tracey, J. Wood, R. E. Dagnew, D. Guan, S. E. London, D. F. Clayton, C. V. Mello, S. R. Friedrich, P. V. Lovell, E. Osipova, F. O. Al-Ajli, S. Secomandi, H. Kim, C. Theofanopoulou, M. Hiller, Y. Zhou, R. S. Harris, K. D. Makova, P. Medvedev, J. Hoffman, P. Masterson, K. Clark, F. Martin, K. Howe, P. Flicek, B. P. Walenz, W. Kwak, H. Clawson, M. Diekhans, L. Nassar, B. Paten, R. H. S. Kraus, A. J. Crawford, M. T. P. Gilbert, G. Zhang, B. Venkatesh, R. W. Murphy, K.-P. Koepfli, B. Shapiro, W. E. Johnson, F. Di Palma, T. Marques-Bonet, E. C. Teeling, T. Warnow, J. M. Graves, O. A. Ryder, D. Haussler, S. J. O’Brien, J. Korlach, H. A. Lewin, K. Howe, E. W. Myers, R. Durbin, A. M. Phillippy, E. D. Jarvis, Towards complete and error-free genome assemblies of all vertebrate species. *Nature* **592**, 737–746 (2021).

59. M. Kolmogorov, J. Yuan, Y. Lin, P. A. Pevzner, Assembly of long, error-prone reads using repeat graphs. *Nat. Biotechnol.* **37**, 540–546 (2019).

60. C.-S. Chin, P. Peluso, F. J. Sedlazeck, M. Nattestad, G. T. Concepcion, A. Clum, C. Dunn, R. O’Malley, R. Figueroa-Balderas, A. Morales-Cruz, G. R. Cramer, M. Delledonne, C. Luo, J. R.

Ecker, D. Cantu, D. R. Rank, M. C. Schatz, Phased diploid genome assembly with single-molecule real-time sequencing. *Nat. Methods* **13**, 1050–1054 (2016).

61. S. Koren, B. P. Walenz, K. Berlin, J. R. Miller, N. H. Bergman, A. M. Phillippy, Canu: Scalable and accurate long-read assembly via adaptive k-mer weighting and repeat separation. *Genome Res.* **27**, 722–736 (2017).

62. P. Kerpedjiev, N. Abdennur, F. Lekschas, C. McCallum, K. Dinkla, H. Strobelt, J. M. Luber, S. B. Ouellette, A. Azhir, N. Kumar, J. Hwang, S. Lee, B. H. Alver, H. Pfister, L. A. Mirny, P. J. Park, N. Gehlenborg, HiGlass: Web-based visual exploration and analysis of genome interaction maps. *Genome Biol.* **19**, 125 (2018).

63. A. Donath, F. Jühling, M. Al-Arab, S. H. Bernhart, F. Reinhardt, P. F. Stadler, M. Middendorf, M. Bernt, Improved annotation of protein-coding genes boundaries in metazoan mitochondrial genomes. *Nucleic Acids Res.* **47**, 10543–10552 (2019).

64. C. L. C. Sætre, F. Eroukhmanoff, K. Rönkä, E. Kluen, R. Thorogood, J. Torrance, A. Tracey, W. Chow, S. Pelan, K. Howe, K. S. Jakobsen, O. K. Tørresen, A chromosome-level genome assembly of the reed warbler (*Acrocephalus scirpaceus*). *Genome Biol. Evol.* **13**, evab212 (2021).

65. M. Schubert, L. Ermini, C. Der Sarkissian, H. Jónsson, A. Ginolhac, R. Schaefer, M. D. Martin, R. Fernández, M. Kircher, M. McCue, E. Willerslev, L. Orlando, Characterization of ancient and modern genomes by SNP detection and phylogenomic and metagenomic analysis using PALEOMIX. *Nat. Protoc.* **9**, 1056–1082 (2014).

66. A. McKenna, M. Hanna, E. Banks, A. Sivachenko, K. Cibulskis, A. Kernytsky, K. Garimella, D. Altshuler, S. Gabriel, M. Daly, M. A. DePristo, The genome analysis toolkit: A MapReduce framework for analyzing next-generation DNA sequencing data. *Genome Res.* **20**, 1297–1303 (2010).

67. T. S. Korneliussen, A. Albrechtsen, R. Nielsen, ANGSD: Analysis of next generation sequencing data. *BMC Bioinformatics.* **15**, 356 (2014).

68. F. Salomonsen, *The Atlantic Alcidae: The Seasonal and Geographical Variation of the Auks Inhabiting the Atlantic Ocean and the Adjacent Waters* (Elanders boktryckeri aktiebolag, 1944), vol. 6 of Göteborgs kungl. vetenskaps- och vitterhets-samhälles Handlingar.

69. J. D. Kapp, R. E. Green, B. Shapiro, A fast and efficient single-stranded genomic library preparation method optimized for ancient DNA. *J. Hered.* **112**, 241–249 (2021).

70. N. Patterson, A. L. Price, D. Reich, Population structure and eigenanalysis. *PLOS Genet.* **2**, e190 (2006).

71. D. H. Alexander, J. Novembre, K. Lange, Fast model-based estimation of ancestry in unrelated individuals. *Genome Res.* **19**, 1655–1664 (2009).

72. J. Meisner, A. Albrechtsen, Inferring population structure and admixture proportions in low-depth NGS data. *Genetics* **210**, 719–731 (2018).

73. L. Skotte, T. S. Korneliussen, A. Albrechtsen, Estimating individual admixture proportions from next generation sequencing data. *Genetics* **195**, 693–702 (2013).

74. G. Garcia-Erill, A. Albrechtsen, Evaluation of model fit of inferred admixture proportions. *Mol. Ecol. Resour.* **20**, 936–949 (2020).

75. P. Danecek, A. Auton, G. Abecasis, C. A. Albers, E. Banks, M. A. DePristo, R. E. Handsaker, G. Lunter, G. T. Marth, S. T. Sherry, G. McVean, R. Durbin; 1000 Genomes Project Analysis Group, The variant call format and VCFtools. *Bioinformatics* **27**, 2156–2158 (2011).

76. S. M. Boca, L. Huang, N. A. Rosenberg, On the heterozygosity of an admixed population. *J. Math. Biol.* **81**, 1217–1250 (2020).

77. M. S. Rasmussen, G. Garcia-Erill, T. S. Korneliussen, C. Wiuf, A. Albrechtsen, Estimation of site frequency spectra from low-coverage sequencing data using stochastic EM reduces overfitting, runtime, and memory usage. *Genetics* **222**, iyac148 (2022).

78. D. L. Duc, A. Velluva, M. Cassatt-Johnstone, R.-A. Olsen, S. Baleka, C.-C. Lin, J. R. Lemke, J. R. Southon, A. Burdin, M.-S. Wang, S. Grunewald, W. Rosendahl, U. Joger, S. Rutschmann, T. B. Hildebrandt, G. Fritsch, J. A. Estes, J. Kelso, L. Dalén, M. Hofreiter, B. Shapiro, T. Schöneberg, Genomic basis for skin phenotype and cold adaptation in the extinct Steller's sea cow. *Sci. Adv.* **8**, eabl6496 (2022).

79. M. V. Westbury, S. Hartmann, A. Barlow, I. Wiesel, V. Leo, R. Welch, D. M. Parker, F. Sicks, A. Ludwig, L. Dalén, M. Hofreiter, Extended and continuous decline in effective population size results in low genomic diversity in the World's Rarest Hyena species, the Brown Hyena. *Mol. Biol. Evol.* **35**, 1225–1237 (2018).

80. S. Kumar, G. Stecher, M. Suleski, S. B. Hedges, TimeTree: A resource for timelines, timetrees, and divergence times. *Mol. Biol. Evol.* **34**, 1812–1819 (2017).

81. J. P. Bird, R. Martin, H. R. Akçakaya, J. Gilroy, I. J. Burfield, S. T. Garnett, A. Symes, J. Taylor, Ç. H. Şekercioğlu, S. H. M. Butchart, Generation lengths of the world's birds and their implications for extinction risk. *Conserv. Biol.* **34**, 1252–1261 (2020).

82. H. Li, R. Durbin, Inference of human population history from individual whole-genome sequences. *Nature* **475**, 493–496 (2011).

83. K. Nadachowska-Brzyska, C. Li, L. Smeds, G. Zhang, H. Ellegren, Temporal dynamics of avian populations during Pleistocene revealed by whole-genome sequences. *Curr. Biol.* **25**, 1375–1380 (2015).

84. X. Liu, Y.-X. Fu, Stairway Plot 2: Demographic history inference with folded SNP frequency spectra. *Genome Biol.* **21**, 280 (2020).

85. S. Schiffels, K. Wang, MSMC and MSMC2: The Multiple Sequentially Markovian Coalescent, in *Statistical Population Genomics*, J. Y. Dutheil, Ed. (Springer, 2020), pp. 147–166.

86. M. Patterson, T. Marschall, N. Pisanti, L. van Iersel, L. Stougie, G. W. Klau, A. Schönhuth, WhatsHap: Weighted haplotype assembly for future-generation sequencing reads. *J. Comput. Biol.* **22**, 498–509 (2015).

87. O. Delaneau, J.-F. Zagury, M. Robinson, J. Marchini, E. Dermitzakis, Integrative haplotype estimation with sub-linear complexity. bioRxiv 493403 (2018). https://doi.org/10.1101/493403.

88. E. Santiago, I. Novo, A. F. Pardiñas, M. Saura, J. Wang, A. Caballero, Recent demographic history inferred by high-resolution analysis of linkage disequilibrium. *Mol. Biol. Evol.* **37**, 3642–3653 (2020).

89. J. K. Pickrell, J. K. Pritchard, Inference of population splits and mixtures from genome-wide allele frequency data. *PLOS Genet.* **8**, e1002967 (2012).

90. M. Malinsky, M. Matschiner, H. Svardal, Dsuite - Fast D-statistics and related admixture evidence from VCF files. *Mol. Ecol. Resour.* **21**, 584–595 (2021).

91. B. K. Maples, S. Gravel, E. E. Kenny, C. D. Bustamante, RFMix: A discriminative modeling approach for rapid and robust local-ancestry inference. *Am. J. Hum. Genet.* **93**, 278–288 (2013).

92. F. Racimo, S. Sankararaman, R. Nielsen, E. Huerta-Sánchez, Evidence for archaic adaptive introgression in humans. *Nat. Rev. Genet.* **16**, 359–371 (2015).

93. D. J. Lawson, G. Hellenthal, S. Myers, D. Falush, Inference of population structure using dense haplotype data. *PLOS Genet.* **8**, e1002453 (2012).

94. P. Wangkumhang, M. Greenfield, G. Hellenthal, An efficient method to identify, date, and describe admixture events using haplotype information. *Genome Res.* **32**, 1553–1564 (2022).

95. M. Duranton, F. Bonhomme, P.-A. Gagnaire, The spatial scale of dispersal revealed by admixture tracts. *Evol. Appl.* **12**, 1743–1756 (2019).

96. H. Li, Aligning sequence reads, clone sequences and assembly contigs with BWA-MEM. arXiv:1303.3997 (2013).

97. X. Zhang, S. Zhang, Q. Zhao, R. Ming, H. Tang, Assembly of allele-aware, chromosomal-scale autopolyploid genomes based on Hi-C data. *Nat. Plants* **5**, 833–845 (2019).

98. J. Ghurye, M. Pop, Modern technologies and algorithms for scaffolding assembled genomes. *PLoS Comput. Biol.* **15**, e1006994 (2019).

99. D. Guan, S. A. McCarthy, J. Wood, K. Howe, Y. Wang, R. Durbin, Identifying and removing haplotypic duplication in primary genome assemblies. *Bioinformatics* **36**, 2896–2898 (2020).

100. N. C. Durand, J. T. Robinson, M. S. Shamim, I. Machol, J. P. Mesirov, E. S. Lander, E. L. Aiden, Juicebox provides a visualization system for Hi-C contact maps with unlimited zoom. *Cell Syst.* **3**, 99–101 (2016).

101. A. Bishara, Y. Liu, Z. Weng, D. Kashef-Haghighi, D. E. Newburger, R. West, A. Sidow, S. Batzoglou, Read clouds uncover variation in complex regions of the human genome. *Genome Res.* **25**, 1570–1580 (2015).

102. E. Garrison, G. Marth, Haplotype-based variant detection from short-read sequencing. arXiv:1207.3907 [q-bio.GN] (2012).

103. K. Howe, W. Chow, J. Collins, S. Pelan, D.-L. Pointon, Y. Sims, J. Torrance, A. Tracey, J. Wood, Significantly improving the quality of genome assemblies through curation. *Gigascience* **10**, giaa153 (2021).

104. D. E. Wood, J. Lu, B. Langmead, Improved metagenomic analysis with Kraken 2. *Genome Biol.* **20**, 257 (2019).

105. R. Challis, E. Richards, J. Rajan, G. Cochrane, M. Blaxter, BlobToolKit - Interactive quality assessment of genome assemblies. *G3 (Bethesda)* **10**, 1361–1374 (2020).

106. D. Guan, S. A. McCarthy, J. M. D. Wood, Y. Sims, W. Chow, Z. Ning, K. Howe, G. Wang, Y. Wang, R. Durbin, Genome sequence assembly evaluation using long-range sequencing data. bioRxiv 2022.05.10.491304 (2022). https://doi.org/10.1101/2022.05.10.491304.

107. M. Manni, M. R. Berkeley, M. Seppey, F. A. Simão, E. M. Zdobnov, BUSCO update: Novel and streamlined workflows along with broader and deeper phylogenetic coverage for scoring of eukaryotic, prokaryotic, and viral genomes. *Mol. Biol. Evol.* **38**, 4647–4654 (2021).

108. A. Mikheenko, A. Prjibelski, V. Saveliev, D. Antipov, A. Gurevich, Versatile genome assembly evaluation with QUAST-LG. *Bioinformatics* **34**, i142–i150 (2018).

109. M. Goel, H. Sun, W.-B. Jiao, K. Schneeberger, SyRI: Finding genomic rearrangements and local sequence differences from whole-genome assemblies. *Genome Biol.* **20**, 277 (2019).

110. F. Cabanettes, C. Klopp, D-GENIES: Dot plot large genomes in an interactive, efficient and simple way. *PeerJ* **6**, e4958 (2018).

111. A. Rhie, B. P. Walenz, S. Koren, A. M. Phillippy, Merqury: Reference-free quality, completeness, and phasing assessment for genome assemblies. *Genome Biol.* **21**, 245 (2020).

112. G. Formenti, L. Abueg, A. Brajuka, N. Brajuka, C. Gallardo-Alba, A. Giani, O. Fedrigo, E. D. Jarvis, Gfastats: Conversion, evaluation and manipulation of genome sequences using assembly graphs. *Bioinformatics* **38**, 4214–4216 (2022).

113. M. Al Arab, C. H. Z. Siederdissen, K. Tout, A. H. Sahyoun, P. F. Stadler, M. Bernt, Accurate annotation of protein-coding genes in mitochondrial genomes. *Mol. Phylogenet. Evol.* **106**, 209–216 (2017).

114. V. Peona, O. M. Palacios-Gimenez, J. Blommaert, J. Liu, T. Haryoko, K. A. Jønsson, M. Irestedt, Q. Zhou, P. Jern, A. Suh, The avian W chromosome is a refugium for endogenous retroviruses with likely effects on female-biased mutational load and genetic incompatibilities. *Philos. Trans. R. Soc. Lond. B Biol. Sci.* **376**, 20200186 (2021).

115. B. D. Ondov, T. J. Treangen, P. Melsted, A. B. Mallonee, N. H. Bergman, S. Koren, A. M. Phillippy, Mash: Fast genome and metagenome distance estimation using MinHash. *Genome Biol.* **17**, 132 (2016).

116. M. Simonsen, T. Mailund, C. N. S. Pedersen, Rapid neighbour-joining, in *Algorithms in Bioinformatics* (Springer Berlin Heidelberg, 2008), pp. 113–122.

117. J. Armstrong, G. Hickey, M. Diekhans, I. T. Fiddes, A. M. Novak, A. Deran, Q. Fang, D. Xie, S. Feng, J. Stiller, D. Genereux, J. Johnson, V. D. Marinescu, J. Alföldi, R. S. Harris, K.

Lindblad-Toh, D. Haussler, E. Karlsson, E. D. Jarvis, G. Zhang, B. Paten, Progressive Cactus is a multiple-genome aligner for the thousand-genome era. *Nature* **587**, 246–251 (2020).

118. I. T. Fiddes, J. Armstrong, M. Diekhans, S. Nachtweide, Z. N. Kronenberg, J. G. Underwood, D. Gordon, D. Earl, T. Keane, E. E. Eichler, D. Haussler, M. Stanke, B. Paten, Comparative annotation Toolkit (CAT)-simultaneous clade and personal genome annotation. *Genome Res.* **28**, 1029–1038 (2018).

119. M. Stanke, M. Diekhans, R. Baertsch, D. Haussler, Using native and syntenically mapped cDNA alignments to improve de novo gene finding. *Bioinformatics* **24**, 637–644 (2008).

120. P. Jones, D. Binns, H.-Y. Chang, M. Fraser, W. Li, C. McAnulla, H. McWilliam, J. Maslen, A. Mitchell, G. Nuka, S. Pesseat, A. F. Quinn, A. Sangrador-Vegas, M. Scheremetjew, S.-Y. Yong, R. Lopez, S. Hunter, InterProScan 5: Genome-scale protein function classification. *Bioinformatics* **30**, 1236–1240 (2014).

121. B. Buchfink, K. Reuter, H.-G. Drost, Sensitive protein alignments at tree-of-life scale using DIAMOND. *Nat. Methods* **18**, 366–368 (2021).

122. UniProt Consortium, UniProt: The universal protein knowledgebase in 2021. *Nucleic Acids Res.* **49**, D480–D489 (2021).

123. J. Dainat, D. Hereñú, E. Davis, K. Crouch, LucileSol, N. Agostinho, pascal-git, tayyrov, NBISweden/AGAT: AGAT-v0.9.2 (2022); https://zenodo.org/record/6621429.

124. S. Lindgreen, AdapterRemoval: Easy cleaning of next-generation sequencing reads. *BMC. Res. Notes* **5**, 337 (2012).

125. Broad Institute, Picard Toolkit; http://broadinstitute.github.io/picard/.

126. H. Li, B. Handsaker, A. Wysoker, T. Fennell, J. Ruan, N. Homer, G. Marth, G. Abecasis, R. Durbin; 1000 Genome Project Data Processing Subgroup, The sequence alignment/map format and SAMtools. *Bioinformatics* **25**, 2078–2079 (2009).

127. G. A. Van der Auwera, M. O. Carneiro, C. Hartl, R. Poplin, G. Del Angel, A. Levy-Moonshine, T. Jordan, K. Shakir, D. Roazen, J. Thibault, E. Banks, K. V. Garimella, D. Altshuler, S. Gabriel, M. A. DePristo, From FastQ data to high confidence variant calls: The genome analysis toolkit best practices pipeline. *Curr. Protoc. Bioinformatics* **43**, 11.10.1–11.10.33 (2013).

128. A. Manichaikul, J. C. Mychaleckyj, S. S. Rich, K. Daly, M. Sale, W.-M. Chen, Robust relationship inference in genome-wide association studies. *Bioinformatics* **26**, 2867–2873 (2010).

129. N. M. Kopelman, J. Mayzel, M. Jakobsson, N. A. Rosenberg, I. Mayrose, Clumpak: A program for identifying clustering modes and packaging population structure inferences across K. *Mol. Ecol. Resour.* **15**, 1179–1191 (2015).

130. G. Evanno, S. Regnaut, J. Goudet, Detecting the number of clusters of individuals using the software STRUCTURE: A simulation study. *Mol. Ecol.* **14**, 2611–2620 (2005).

131. A. Prasad, E. D. Lorenzen, M. V. Westbury, Evaluating the role of reference-genome phylogenetic distance on evolutionary inference. *Mol. Ecol. Resour.* **22**, 45–55 (2022).

132. T. Kawakami, L. Smeds, N. Backström, A. Husby, A. Qvarnström, C. F. Mugal, P. Olason, H. Ellegren, A high-density linkage map enables a second-generation collared flycatcher genome assembly and reveals the patterns of avian recombination rate variation and chromosomal evolution. *Mol. Ecol.* **23**, 4035–4058 (2014).

133. P. U. Clark, A. S. Dyke, J. D. Shakun, A. E. Carlson, J. Clark, B. Wohlfarth, J. X. Mitrovica, S. W. Hostetler, A. M. McCabe, The last glacial maximum. *Science* **325**, 710–714 (2009).

134. M. E. Mann, Z. Zhang, S. Rutherford, R. S. Bradley, M. K. Hughes, D. Shindell, C. Ammann, G. Faluvegi, F. Ni, Global signatures and dynamical origins of the little ice age and medieval climate anomaly. *Science* **326**, 1256–1260 (2009).

135. N. P. McKay, D. S. Kaufman, C. C. Routson, M. P. Erb, P. D. Zander, The onset and rate of Holocene Neoglacial cooling in the arctic. *Geophys. Res. Lett.* **45**, 12,487–12,496 (2018).

136. A. B. Patil, N. Vijay, Repetitive genomic regions and the inference of demographic history. *Heredity* **127**, 151–166 (2021).

137. R. S. Taylor, M. Manseau, C. F. C. Klütsch, J. L. Polfus, A. Steedman, D. Hervieux, A. Kelly, N. C. Larter, M. Gamberg, H. Schwantje, P. J. Wilson, Population dynamics of caribou shaped by glacial cycles before the last glacial maximum. *Mol. Ecol.* **30**, 6121–6143 (2021).

138. C. Pockrandt, M. Alzamel, C. S. Iliopoulos, K. Reinert, GenMap: Ultra-fast computation of genome mappability. *Bioinformatics* **36**, 3687–3692 (2020).

139. L. Deng, B. Xie, Y. Wang, X. Zhang, S. Xu, A protocol for applying a population-specific reference genome assembly to population genetics and medical studies. *STAR Protoc.* **3**, 101440 (2022).

140. M. Duranton, F. Allal, S. Valière, O. Bouchez, F. Bonhomme, P.-A. Gagnaire, The contribution of ancient admixture to reproductive isolation between European sea bass lineages. *Evol Lett.* **4**, 226–242 (2020).

141. O. G. Zedlitz, Ornithologische Notizen von der “Zeppelin-Studienfahrt” Spitzbergen Sommer 1910 (1911); https://zenodo.org/record/2361488.

142. J. E. McCormack, W. L. E. Tsai, B. C. Faircloth, Sequence capture of ultraconserved elements from bird museum specimens. *Mol. Ecol. Resour.* **16**, 1189–1203 (2016).

143. S. Feng, Q. Fang, R. Barnett, C. Li, S. Han, M. Kuhlwilm, L. Zhou, H. Pan, Y. Deng, G. Chen, A. Gamauf, F. Woog, R. Prys-Jones, T. Marques-Bonet, M. T. P. Gilbert, G. Zhang, The genomic footprints of the fall and recovery of the crested Ibis. *Curr. Biol.* **29**, 340–349.e7 (2019).

144. M. T. P. Gilbert, H.-J. Bandelt, M. Hofreiter, I. Barnes, Assessing ancient DNA studies. *Trends Ecol. Evol.* **20**, 541–544 (2005).

145. B. Llamas, G. Valverde, L. Fehren-Schmitz, L. S. Weyrich, A. Cooper, W. Haak, From the field to the laboratory: Controlling DNA contamination in human ancient DNA research in the high-throughput sequencing era. *STAR* **3**, 1–14 (2017).

146. R Core Team, R: *A Language and Environment for Statistical Computing* (R Foundation for Statistical Computing 2020); www.R-project.org/.

147. E. A. Fox, A. E. Wright, M. Fumagalli, F. G. Vieira, ngsLD: Evaluating linkage disequilibrium using genotype likelihoods. *Bioinformatics* **35**, 3855–3856 (2019).

148. S. Van Dongen, Graph clustering via a discrete uncoupling process. *SIAM J. Matrix Anal. Appl.* **30**, 121–141 (2008).

149. L. Li, C. J. Stoeckert Jr, D. S. Roos, OrthoMCL: Identification of ortholog groups for eukaryotic genomes. *Genome Res.* **13**, 2178–2189 (2003).

150. M. V. Matz, Fantastic beasts and how to sequence them: Ecological genomics for obscure model organisms. *Trends Genet.* **34**, 121–132 (2018).

151. H. B. Mann, D. R. Whitney, On a test of whether one of two random variables is stochastically larger than the other. *Ann. Math. Stat.* **18**, 50–60 (1947).
